# Enhanced Efflux Activity Facilitates Drug Tolerance in Dormant Bacterial Cells

**DOI:** 10.1016/j.molcel.2016.03.035

**Published:** 2016-04-21

**Authors:** Yingying Pu, Zhilun Zhao, Yingxing Li, Jin Zou, Qi Ma, Yanna Zhao, Yuehua Ke, Yun Zhu, Huiyi Chen, Matthew A.B. Baker, Hao Ge, Yujie Sun, Xiaoliang Sunney Xie, Fan Bai

**Affiliations:** 1 Biodynamic Optical Imaging Center (BIOPIC), School of Life Sciences, Peking University, Beijing, China, 100871; 2 Department of Chemistry and Chemical Biology, Harvard University, Cambridge, MA 02138, USA; 3 Victor Chang Cardiac Research Institute, Sydney, NSW 2010, Australia; 4 Beijing International Center for Mathematical Research, Peking University, Beijing, China, 100871

## Abstract

Natural variations in gene expression provide a mechanism for multiple phenotypes to arise in an isogenic bacterial population. In particular, a sub-group termed persisters show high tolerance to antibiotics. Previously, their formation has been attributed to cell dormancy. Here we demonstrate that bacterial persisters, under β-lactam antibiotic treatment, show less cytoplasmic drug accumulation as a result of enhanced efflux activity. Consistently, a number of multi-drug efflux genes, particularly the central component TolC, show higher expression in persisters. Time-lapse imaging and mutagenesis studies further establish a positive correlation between *tolC* expression and bacterial persistence. The key role of efflux systems, among multiple biological pathways involved in persister formation, indicates that persisters implement a positive defense against antibiotics prior to a passive defense via dormancy. Finally, efflux inhibitors and antibiotics together effectively attenuate persister formation, suggesting a combination strategy to target drug tolerance.

## Highlights

• Persisters accumulate fewer antibiotics as a direct result of increased efflux rate
• Persisters show higher expression of efflux-associated genes
• High expression of *tolC* is critical to promote persister formation
• Persisters combine active efflux and passive dormancy to survive antibiotic attack

## Graphical Abstract

**Figure:**
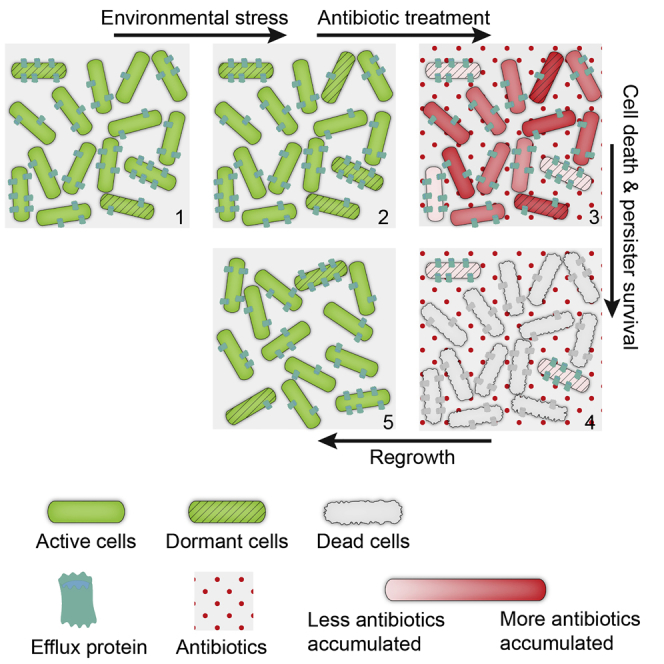

## Teaser

By using in vivo fluorescent imaging and next-generation sequencing, Pu et al. demonstrate that bacterial persisters exhibit enhanced efflux activity in addition to physiological quiescence, a double insurance strategy to ensure its survival under antibiotic attack.

## Introduction

Multi-level stochasticity arising from mRNA transcription and protein translation leads to remarkable phenotypic heterogeneity in a population of cells with an identical genome (Elowitz et al., 2002, Lidstrom and Konopka, 2010, Raj and van Oudenaarden, 2008). Such non-genetic individuality is often proposed as a ‘bet-hedging” mechanism (Beaumont et al., 2009, Losick and Desplan, 2008, Veening et al., 2008), whereby a clonal cell population maximizes its survival chance under rapidly changing environment by exploring diverse phenotypic solutions. One phenotype may gain a higher fitness under a given condition, and other phenotypes may be better adapted to certain other environmental situations. In the case of bacteria under drug treatment, while the majority of cells are quickly eradicated by bactericidal antibiotics, a very small group of phenotypic variants, termed persisters, show strong drug tolerance. This phenomenon was first reported in 1944 by Joseph Bigger when he studied the lethal effect of penicillin on *Staphylococcus aureus* (Bigger, 1944). Bacterial persisters have also been observed in other pathogenic species, and they may play a role in the recurrence of chronic infections. Their existence is believed to prolong and exacerbate the treatment of diseases, such as tuberculosis, cystic fibrosis associated lung infections, and candidiasis (Boucher, 2001, Chao and Rubin, 2010, Fauvart et al., 2011, LaFleur et al., 2006, Mulcahy et al., 2010).

To develop new drugs targeting chronic infections requires a deep understanding of the mechanisms underlying persister formation. However, the extremely low percentage of persister cells in a bacterial population and complex pathways involved in persister formation have delayed the study of this phenomenon. Previous studies associated persister formation mainly with cell dormancy (Balaban et al., 2004, Lewis, 2007, Shah et al., 2006), in which the important contribution from toxin-antitoxin (TA) loci has been highlighted (Balaban et al., 2004, Dörr et al., 2010, Keren et al., 2004, Vázquez-Laslop et al., 2006). TA locus encodes two components: a stable toxin that can interrupt essential cellular pathways and induce a dormancy-like state and a labile antitoxin that can conjugate the toxin to nullify such toxicity. TA modules are most likely activated by stress responses through the alarmone guanosine tetraphosphate (ppGpp) pathway (Maisonneuve et al., 2013). Furthermore, the SOS response induces persister formation through activating TisB overexpression (Dörr et al., 2010), a member of the toxin family as well. Screening a complete bacterial knockout library also identified a number of global regulators involved in persister formation (Hansen et al., 2008). For example, overexpression of *ygfA* downregulated overall transcription, and overexpression of *relE* led to a decreased protein synthesis rate (Maisonneuve et al., 2011), both of which assist bacterial drug tolerance through inducing a dormant state of the cell.

The leading theory explaining antibiotic tolerance of persister cells lies in the fact that even though antibiotic molecules bind their targets, their lethal effects have been disabled due to the extremely slow metabolic and proliferation rates in those dormant cells (Lewis, 2007). However, one fundamental question remains poorly addressed: do antibiotics effectively enter and accumulate in persister cells to exert their lethal effects? Antibiotic accumulation in Gram-negative bacteria is primarily influenced by two factors, membrane permeability and efflux activity. Hydrophobic antibiotics, such as aminoglycosides and macrolides, gain access into the cell through the membrane by metabolic activity-dependent diffusion, while hydrophilic antibiotics, such as β-lactam, enter the cell through porin channels to reach their targets (Delcour, 2009). Inversely, bacterial multi-drug efflux systems actively pump antibiotics out to reduce cellular drug accumulation, thus facilitating bacterial survival (Sun et al., 2014, Webber and Piddock, 2003). Therefore, stochastic and heterogeneous expression of porin or efflux proteins could lead to uneven antibiotic accumulation and consequently different drug tolerance across a bacterial population. Whether these two systems play roles in bacterial persistence thus deserves in-depth investigation.

In this study we combined in vivo fluorescent imaging and next-generation sequencing to explore the critical biological mechanisms that produce bacterial persistence. We found that enhanced efflux activity contributed strongly to persister formation and that bacterial persisters adopt a two-pronged strategy to ensure survival under antibiotic attack. Most biological processes were slowed down during dormancy, yet the efflux systems, on the contrary, displayed greater activity that further increased persister tolerance to antibiotics.

## Results

### Antibiotic Accumulates at a Lower Level in Persister Cells

**Figure 1:**
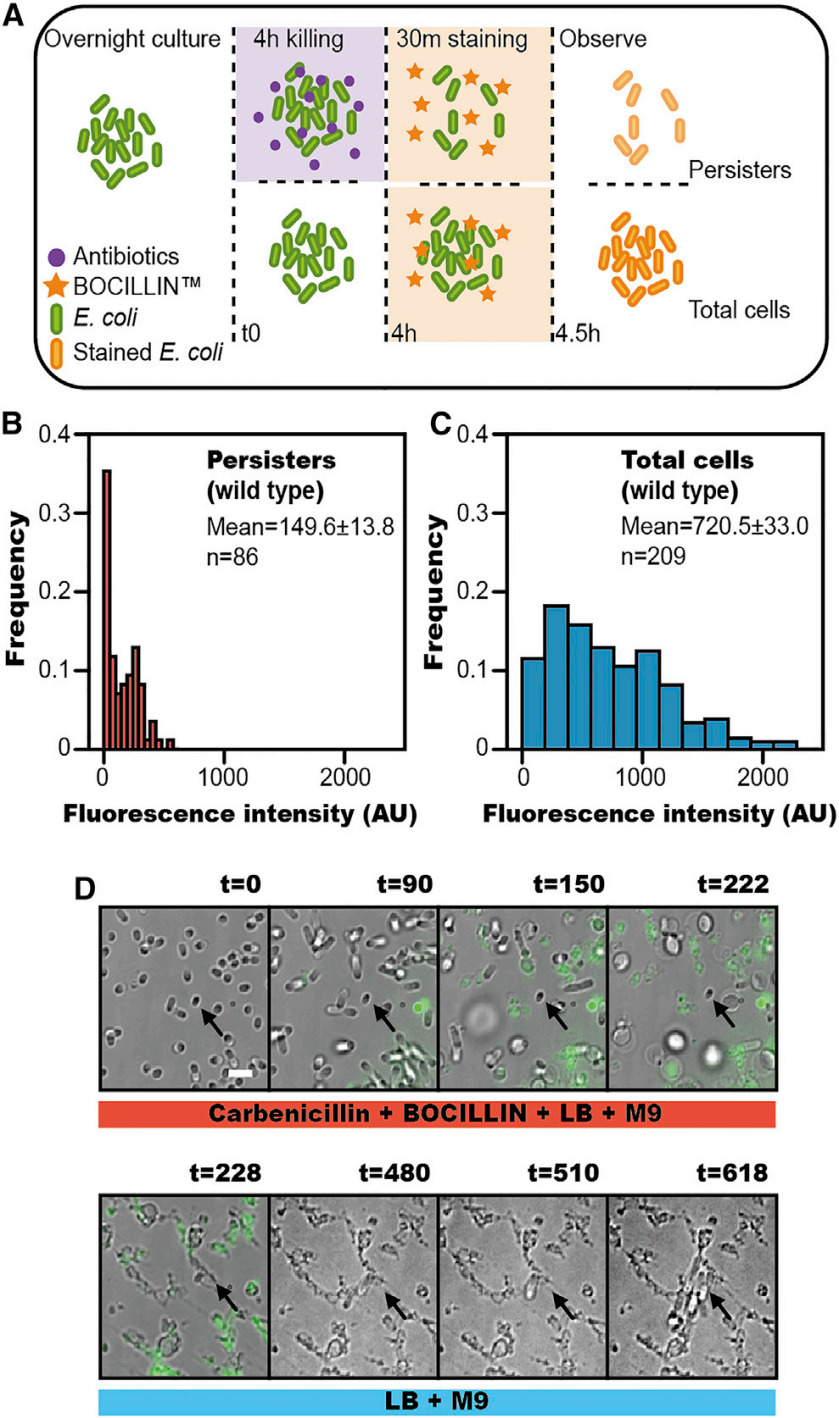
Lower Intracellular Antibiotic Accumulation in Persister Cells of Wild-Type *E. coli* (A) Experimental procedure for measuring antibiotic accumulation in bacterial cells. (B and C) Histogram of antibiotic accumulation in total cells (n = 209) and persister cells (n = 86) of wild-type *E. coli*. (D) Time-lapse microscopy showing reduced accumulation of antibiotic in persisters (from Movie S1). The first five images are merged bright field and fluorescence images, revealing that antibiotic accumulation accompanies cell death (separate images are shown in Figure S1F). The different media added during experiment is indicated below (killing medium: 90% [v/v] M9 + 10% [v/v] LB + 150 μg/ml carbenicillin + 20 μg/ml BOCILLIN + 0.15% [w/v] methylcellulose; growth medium: 90% [v/v] M9 + 10% [v/v] LB + 5% [w/v] methylcellulose) (Maisonneuve et al., 2013). The persister cell (arrow) shows low antibiotic accumulation and regrows after removal of antibiotic (Scale bar, 3 μm; t = time in min). See also Figure S1 and Movie S1.

To investigate the antibiotic accumulation in bacterial persisters, we used a fluorescent β-lactam antibiotic BOCILLIN™ FL Penicillin (BOCILLIN) to measure quantitatively single cell intracellular drug concentration. We isolated persisters after 150 μg/ml carbinicillin treatment (see Experimental Procedures). These drug tolerant cells had not genetically acquired resistance—after removing the antibiotic, they regrew a new bacterial population in which the majority of cells was still vulnerable to the same antibiotic (Figure S1A). Furthermore, according to previously established staining protocol (Orman and Brynildsen, 2013a), we combined fluorescence microscopy with colony counting assay to confirm that persister cells, not the viable but non-culturable cells (VBNCs), were the main cell type in these drug tolerant population (Figures S1B–S1E). After isolating persister cells, we incubated them for 30 min at 37°C (Figure 1A) with BOCILLIN. In parallel, the same staining process was carried out on a population of untreated cells that contained both persisters and susceptible cells. The fluorescent signal corresponding to antibiotic concentration in individual cells was then imaged and normalized by the cell size (the 2D projection area of the cell in bright field imaging). We followed previously described methods (Taniguchi et al., 2010) to remove background fluorescence and cell auto-fluorescence, and signal from dead cells (Figure S1B). We compared the fluorescent antibiotic intensity accumulated in bacterial persisters with that in total cells by analyzing ∼150 cells from each population. Fluorescent antibiotic was found to accumulate in bacterial persisters, excluding the possibility that persisters are impermeable to antibiotics. However, the average fluorescence intensity in persisters (149.6 AU/pixel, Figure 1B) was only ∼1/5 of that in total cells (720.5 AU/pixel, Figure 1C), indicating that the accumulated antibiotic concentration in persisters was substantially lower than that in total cells.

Additionally, we used single-cell time-lapse fluorescence microscopy to track antibiotic induced cell death and persister regrowth. As shown in Figures 1D and S1F and Movie S1, the cells with low antibiotic accumulation tolerated antibiotic attack, whereas the cells with high antibiotic accumulation gradually died and lysed. These persister cells exhibited dormancy during our time-lapse recording, featured by non-growth and non-division, but later resumed growth after the antibiotic was removed from the medium. Our observations revealed a negative correlation between the amount of antibiotic accumulated inside a cell and its probability of persisting. This proved that a reduction in the accumulation of antibiotic contributed directly to bacterial persistence.

### Persister Cells Exhibit Increased Efflux Pump Activity

**Figure 2:**
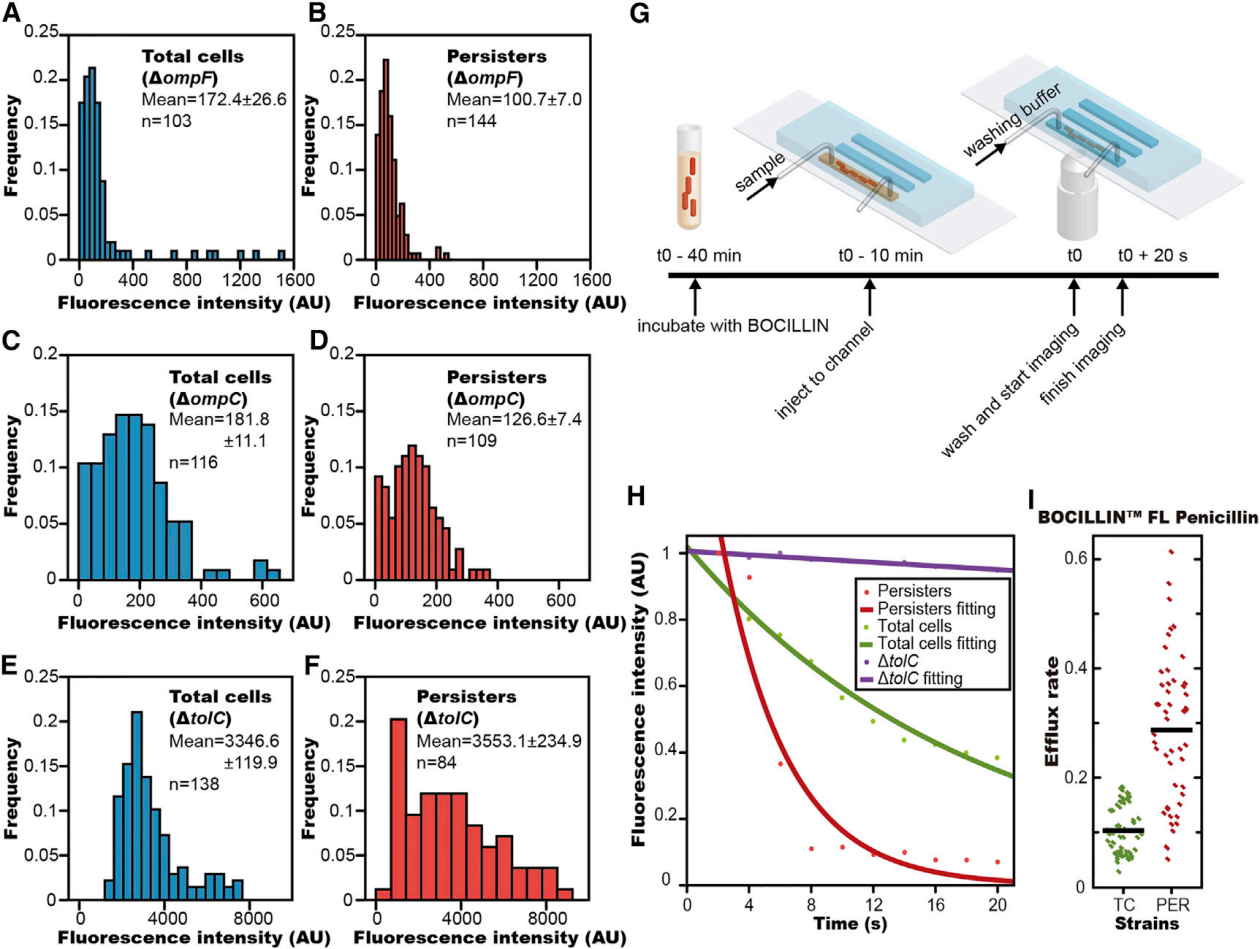
High Efflux Activity in Persister Cells (A and B) Histogram of antibiotic accumulation in total cells (n = 103) and persister cells (n = 144) of *ΔompF* strain. (C and D) Histogram of antibiotic accumulation in total cells (n = 116) and persister cells (n = 109) of *ΔompC* strain. (E and F) Histogram of antibiotic accumulation in total cells (n = 138) and persister cells (n = 84) of *ΔtolC* strain. (G) Experimental procedure for measuring efflux rate of fluorescent antibiotic in bacterial cells. (H) Intracellular fluorescent intensity decay after removing antibiotic in the medium is well fit by a single exponential function. Fluorescent intensity in a persister cell (red) decayed more rapidly than in a susceptible cell (green), while a cell of *ΔtolC* mutant (purple) showed little change in fluorescence intensity with time. (I) Statistical analysis showing that the efflux rate of antibiotic in persister cells is significantly higher than that in total cells of wild-type *E. coli* (TC, total cells; PER, persisters) (p < 0.0001). See also Figure S2 and Movie S2.

Antibiotic accumulation in persister cells can be lowered by decreasing the membrane permeability, increasing the efflux rate, or a combination of both. To examine exactly how antibiotic levels were lowered in persisters, we constructed two porin knockout mutants (*ΔompF* and *ΔompC*) and an efflux knockout mutant (*ΔtolC*). Using our earlier method, we compared the effect of these knockouts on each subpopulation of persisters as well as the total cell population. OmpF and OmpC are the major porin channel proteins that facilitate penetration of β-lactam antibiotics through the outer membrane (Jaffe et al., 1982, Ziervogel and Roux, 2013) and TolC is the central component of a number of bacterial efflux pumps, responsible for exporting cellular β-lactam antibiotics. First, for porin genes, we observed that the average fluorescence intensity of the total cell population in *ΔompF* mutant was 172.4 AU/pixel (Figure 2A), and in the *ΔompC* mutant the intensity was 181.8 AU/pixel (Figure 2C). This was a 4-fold decrease in intensity in comparison with the wild-type total cell population (720.5 AU/pixel, Figure 1C). This indicated that disabling porin led to lower antibiotic accumulation in all cells. In the subpopulation of persisters, the average fluorescence intensity of the mutants was roughly a further two-thirds lower. The *ΔompF* mutant in the persister cells was 100.7 AU/pixel (Figure 2B), and the *ΔompC* mutant in persister cells was 126.6 AU/pixel (Figure 2D). This indicated that additional factors were contributing to lowering the antibiotic accumulation in persister cells.

On the other hand, for efflux genes, the average fluorescence intensity in *ΔtolC* mutant was 3,346.6 AU/pixel (Figure 2E), a massive increase compared to the value of the wild-type (720.5 AU/pixel, Figure 1C), indicating that disabling efflux lead to extremely high antibiotic accumulation. Surprisingly, the average fluorescence intensity of persisters in *ΔtolC* mutant was 3,553.1 AU/pixel (Figure 2F), almost equal to the value in the total cells of *ΔtolC* mutant. This indicated that efflux plays a critical role in reducing cellular antibiotic concentration in persister cells of wild-type. Combined, these results suggest both decreased membrane permeability and increased efflux activity contribute to the reduction of antibiotic accumulation in wild-type persisters, but that the latter may play a more critical role.

To determine quantitatively the different efflux rates between wild-type persister and total cells, we measured the fluorescence decay of individual cells incubated in microfluidic channels (Figure S2A) by time-lapse imaging, after a sudden removal of BOCILLIN in the medium (Figure 2G; Movie S2). The fluorescence intensity in each cell decayed very quickly in the initial 20 s and then plateaued (Figure 2H). We fit the initial 20 s decay of each cell to a single exponential function *f*(*x*) = *a^∗^exp*(−*b*^∗^*t*), where *a* represented the initial intensity and *b* denoted the decay rate. The decay rate had contributions from both the efflux rate and the photobleaching rate of BOCILLIN. As a negative control, we showed that the fluorescence decay in the *ΔtolC* mutant, where efflux had been deactivated, was very slow (Figure 2H). Furthermore, when treating with efflux inhibitor, the fluorescence stabilizes in the persisters (Figure S2B). These results demonstrated that photobleaching and diffusion made only a minor contribution to the intensity decay and that the fast decay observed in wild-type cells arose primarily from the efflux. Also, if we recorded for a longer time, the fluorescence of all cell types reached a plateau close to cell auto-fluorescence, excluding the possibility that BOCILLIN was covalently incorporated into the peptidoglycan of cells. We evaluated ∼50 cells for each cell type, and the mean time constant (b) for decay was 0.32/s in persisters but 0.095/s in normal cells (Figure 2I), indicating that the efflux rate in persisters was approximately 3 times (p value < 0.0001) larger than that in total cells. This result strongly suggests that the lower intracellular antibiotic accumulation observed in bacterial persisters is dependent on their increased efflux activities.

### Multi-Drug Efflux Genes Exhibit Significantly Higher Expression Levels in Persister Cells

**Figure 3:**
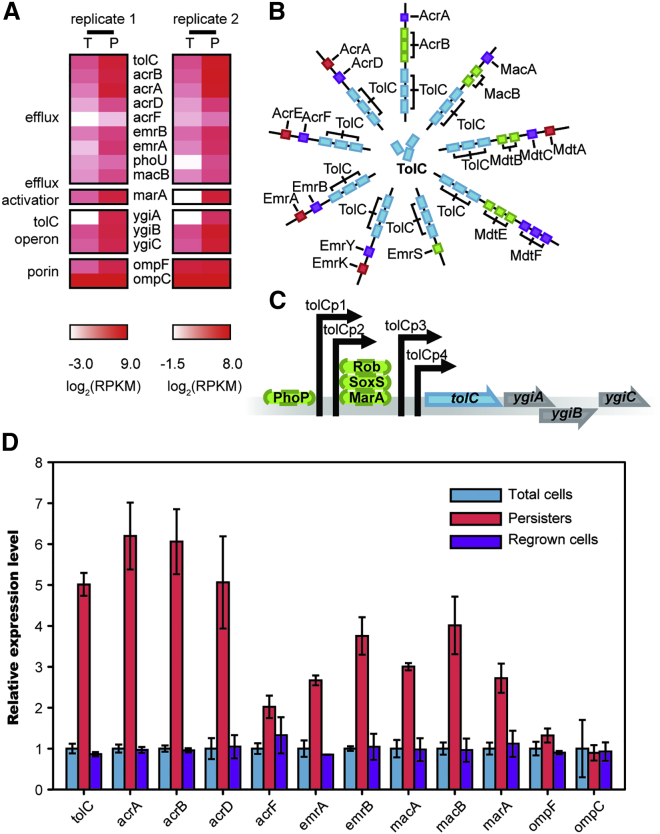
Efflux Gene Expression in Total and Persister Cells of Wild-Type *E. coli* Measured by RNA-seq and qPCR (A) Heatmap showing relative transcript abundance of persistence related genes in total cells and persister cells. Scale below the heatmap indicates log2-normalized transcript abundance relative to the mean expression level (T, total cells; P, persister cells). See also Table S1. (B) Scheme of the TolC related efflux system complexes, the number of cylinders indicating the copy number of the protein in each efflux complex. (C) Scheme of the *tolC* operon. (D) Relative gene expression level in total cells, persister cells, and regrown cells of wild-type *E. coli* measured by qPCR. The bars indicate mean of at least three independent experiments; error bar indicates SD.

Based on the above results, we hypothesized that less antibiotic accumulated in persister cells due to lower expression of porin genes and/or higher expression of efflux genes. Therefore, we performed genome-wide gene expression profiling to compare persisters and total cells using RNA-seq. Two biological replicates of persisters and total cells were prepared to assure reproducibility, and these RNA-seq results are summarized in Table S1. A large number of multi-drug efflux-associated genes showed significantly higher expression (>3-fold, p value < 0.0001) in persisters, including *tolC*, *acrA*, *acrB*, *acrD*, *acrF*, *emrA*, *emrB*, *macA*, and *macB* (Figure 3A). TolC is a common channel protein of both major and minor efflux systems, enabling interaction with many translocase complexes (Figure 3B). Deletion of the *tolC* gene alone in *E. coli* largely abolishes efflux activity (Figures 2E and 2F). Further analysis of sequencing data showed that the expression level of *marA*, one important transcriptional activator of the *tolC* operon, exhibited a far higher expression level in persisters (>10-fold, p value < 0.0001), whereas other genes *ygiA*, *ygiB*, and *ygiC*, from the *tolC* operon (Figure 3C), showed similar expression levels to *tolC*. In contrast, the porin genes *ompF* and *ompC* showed only slightly higher transcriptional levels in persisters, indicating more porin channels on the membrane of those cells. Together these results demonstrated that higher efflux rate, rather than lower membrane permeability, was responsible for reduced antibiotic accumulation in persister cells. qRT-PCR (Figure 3D) agreed with sequencing results and confirmed that these efflux genes were upregulated in persister cells, whereas β-lactam influx porin genes *ompF* and *ompC* had a similar expression level or were only slightly upregulated in persister cells. Both transcriptome sequencing and qRT-PCR results revealed that *tolC* expression in persisters was at least 8-fold greater than that in total cells, making it a potential biomarker to differentiate persisters from normal cells. Whole-genome sequencing (Table S2) and qRT-PCR (Figure 3D) of persister and regrown populations confirmed that enhanced expression of efflux genes is transient and reversible, featuring a non-genetic factor that assists bacterial persistence.

### High Expression Level of *tolC* Directly Assists Bacterial Persistence

**Figure 4:**
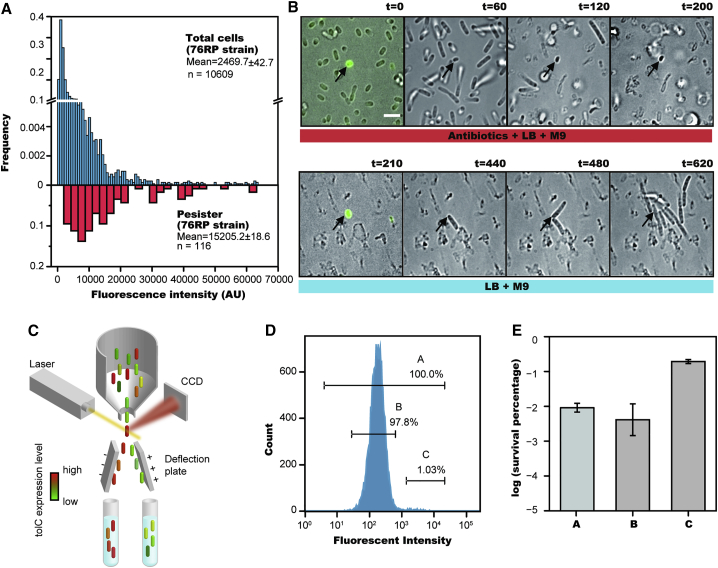
The Dependence between Persister Formation Frequency and TolC Expression Level (A) TolC expression level in total cells of TC tag-TolC strain measured by Tetracysteine-based protein detection (FlAsH imaging, upper panel); TolC expression level in persister cells that survived antibiotic treatment (lower panel). (B) Time-lapse microscopy showing that persister cells express a high level of TolC (from Movie S3). The first and fifth images are the merged bright field and fluorescent images (separate bright field and fluorescent images are shown in Figure S3D). The different media added during the experiment are indicated below (killing medium: 90% [v/v] M9 + 10% [v/v] LB + 150 μg/ml carbenicillin + 0.15% [w/v] methylcellulose; growth medium: 90% [v/v] M9 + 10% [v/v] LB + 5% [w/v] methylcellulose). The persister cell (arrow), shows a significantly higher expression of TolC, and regrows after removal of antibiotic (from Movie S3). (Scale bar, 3 μm; t = time in min). (C) Experimental procedure for sorting cells by expression level of persistence related genes. (D) Distribution of fluorescence intensity indicating TolC expression levels of 76RP strain. Stationary-phase cells were sorted into three groups: group A containing the total cells (100.0%), group B including the majority of total cells except for those with highest fluorescence intensity (97.8%), and group C containing a sub-population with highest fluorescence intensity (1.0%). (E) Bar plot representing cell survival rate (percentage log scale) after 4 hr carbenicillin treatment of three groups sorted by flow cytometer, revealing that the survival rate significantly increases in group C. The bars indicate mean of at least three independent experiments; error bar indicates SD. See also Figure S3 and Movie S3.

We next used single-cell fluorescence imaging to measure the dependence of bacterial persistence on expression level of the central efflux component TolC. First, we monitored the natural fluctuation in concentration of TolC among the cell population. Since trimers of TolC form barrel-like channels in functioning efflux pumps on the outer membrane (Du et al., 2014), conventional labeling of TolC by fluorescent proteins has been difficult. To solve this, we inserted a six amino acid tetracysteine tag into the linker structure of chromosomally encoded *tolC* (TC tag-TolC strain, 76RP). This had little impact on either TolC expression level or function (Figures S3A–S3C). In this way, the tagged TolC protein was fluorescently labeled by tetracysteine-based protein detection (FlAsH labeling) (Griffin et al., 1998). As shown in Figure 4A (upper panel), the fluorescence intensity of TolC-FlAsH across a population of total cells was distributed in a broad gamma distribution with a long tail at high fluorescence, indicating a noticeable heterogeneous expression of *tolC* among different cells.

We monitored the antibiotic killing process under a microscope using time-lapse imaging at controlled temperature. After 4 hr treatment with high concentration (150 μg/ml) carbenicillin, the majority of the cells had died and lysed, leaving only a few surviving persisters. By tracking the spatial coordinates of these persister cells, we found their original positions in the cell population and calculated their TolC-FlAsH fluorescent intensity prior to the antibiotic attack. We found persisters emerged mostly from the long high-intensity tail of the TolC-FIAsH fluorescence distribution (81.0% of persister cells arose from 8.5% of total cell population with highest *tolC* expression) (Figure 4A, lower panel).

Typical examples capturing the dynamic process of persister formation are shown in Figures 4B and S3D and Movie S3. In a single field of view, many cells were susceptible to carbenicillin and lysed gradually. They were featured by low TolC-FIAsH intensity. In contrast, persisters occurred rarely and showed high intensity in TolC-FIAsH fluorescence. We confirmed again that the persister cells neither grew nor divided during our time-lapse recording, consistent with cell dormancy. We then tested whether these cells could resurrect when antibiotics were removed from the growth medium. As shown in Figure 4B and Movie S3, a short delay after antibiotic removal, the persisters, indicated by high fluorescence intensity of TolC-FlAsH, began to elongate, divide, and produce new microcolonies.

Having observed that all persisters exhibit higher *tolC* expression, we sought to examine whether all cells displaying high *tolC* expression were more likely to form persisters. We utilized FACS to group cells according to their TolC level, quantified by FIAsH staining in the 76RP strain (Figure 4C). Both staining and cell sorting did not affect persister formation frequency (Figure S3E). The antibiotic sensitivity assay was subsequently performed on each group of cells to evaluate the frequency of persister formation. As presented in Figures 4D and 4E, the highly fluorescent subpopulation (1%, group C) expressing TC tag-TolC showed 18-fold enrichment in the number of persister cells in comparison with that of the total population (100%, group A), and 24-fold enrichment in comparison with the remaining dimmer population (97.8%, group B). Altogether, this positive correlation between *tolC* expression level and probability of persister formation suggested that high expression level of *tolC* is critical to promote persister formation.

### Bacterial Persistence Negatively Correlates with Intracellular Antibiotic Accumulation and Positively Correlates to Efflux Activity

**Figure 5:**
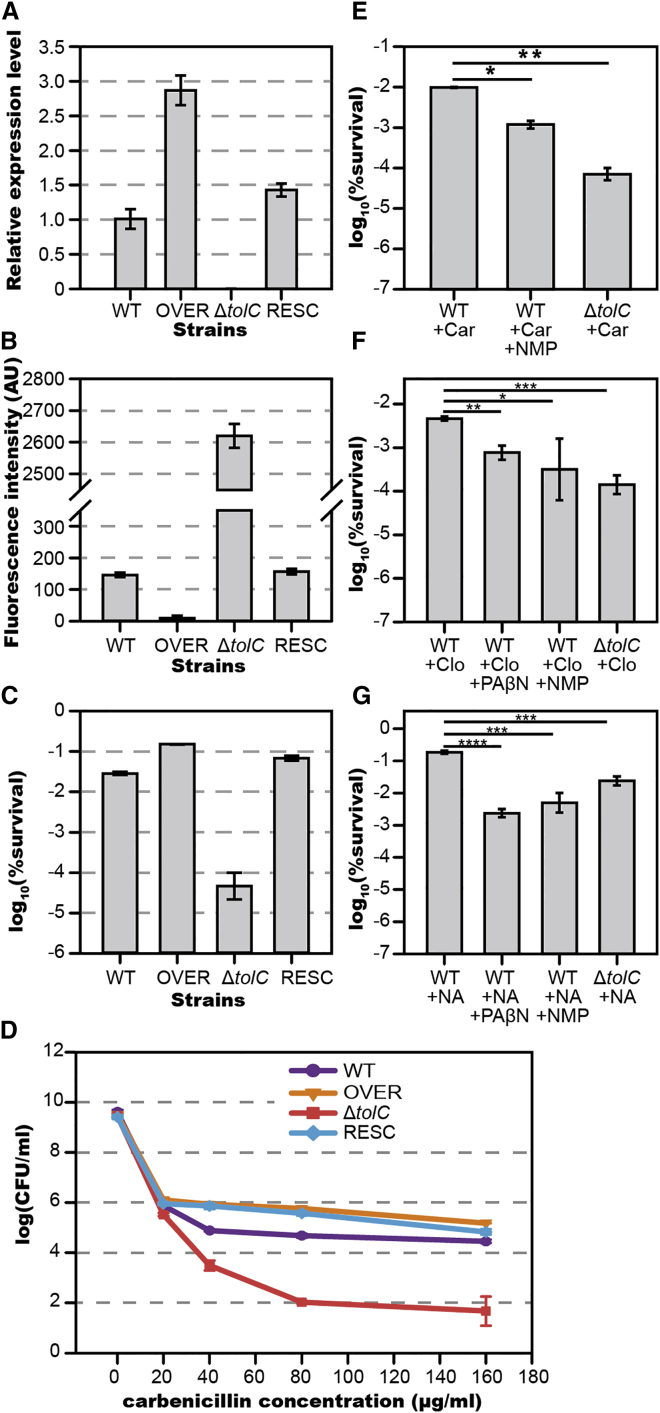
Persister Formation Frequency Positively Correlates with Efflux Gene Expression Level and Negatively Correlates with Intracellular Antibiotic Accumulation. Antibiotics Lethal Effects Were Enhanced by Addition of Efflux Inhibitors (A) Relative *tolC* expression level of the four strains measured by qPCR. WT (wild-type strain BW25113), OVER (*tolC* overexpression strain, by pBAD::*tolC* in BW25113, induced by 10^-5%^ arabinose), *ΔtolC* (*tolC* knockout strain, Key::JW5503), and RESC (*tolC* rescued strain, by pBAD::*tolC* in *ΔtolC* strain, induced by 10^-6%^ arabinose). (B) Relative antibiotic accumulation in the four strains determined by fluorescence microscopy. (C) Persister formation frequency of the four strains determined by antibiotic susceptibility measurement. The correlation coefficiency between intracellular antibiotic accumulation level and *tolC* expression level is −0.776; between intracellular antibiotic accumulation level and probability of persistence is −0.989; between *tolC* expression level and probability of persistence is 0.848. (D–G) (D) Persister formation frequency of the four strains under antibiotic treatment of different concentrations. Antibiotics lethal effects were enhanced by efflux inhibitors; carbenicillin (E), cloxacillin (F), and nalidixic acid (G). Abbreviations: Car, carbenicillin. NA, nalidixic acid. Clo, cloxacillin. PAβN, Phenylalanine arginyl β-naphthylamide. NMP, 1-(1-Naphthylmethyl) piperazine. The bars indicate mean of at least three independent experiments; error bar indicates SD (^∗^p value < 0.1; ^∗∗^p value < 0.01; ^∗∗∗^p value < 0.001; ^∗∗∗∗^p value < 0.0001). See also Figure S4.

We quantified the correlation between efflux gene expression; intracellular antibiotic accumulation; and persister formation frequency using wild-type *E. coli*, *ΔtolC*, *tolC* overexpression, and *tolC* rescue strains. First, we measured the *tolC* gene expression by qRT-PCR. In the overexpression strain, *tolC* concentration was about 3-fold of that in the wild-type strain; no *tolC* expression was detected in the *ΔtolC* strain; and in the rescue strain, *tolC* expression returned to a similar level as that in the wild-type strain (Figure 5A). We measured the antibiotic accumulation in these strains with BOCILLIN using the above method. Fluorescent antibiotic accumulation was negatively correlated with *tolC* expression, with significantly higher intensities (∼2,600 AU/pixel) in the *ΔtolC* strain and lower intensities (∼10 AU/pixel) in the overexpression strain. In the rescued strain, antibiotic accumulation was at a similar level as that in the wild-type strain (Figure 5B). We then used an antibiotic sensitivity assay to measure the frequency of persister formation. The initial concentration of these strains were all set to be ∼5 × 10^7^ cells/ml before antibiotic treatment. While wild-type *E. coli* produced ∼3 × 10^4^ cells/ml antibiotic-tolerant persisters, the *ΔtolC* mutant produced only ∼50 cells/ml (Figures 5C and 5D). The overexpression strain produced ∼1.6 × 10^5^ cells/ml persister cells, roughly 5-fold the number produced by the wild-type strain (Figures 5C and 5D). The rescued strain recovered the low-persister frequency in the *ΔtolC* strain (Figures 5C and 5D). Thus, the *tolC* expression level was negatively correlated to the intracellular antibiotic accumulation, and positively correlated to persister formation.

### Efflux Pump Inhibitors Combined with Antibiotics to Eradicate Persisters

Since persister formation frequency was dramatically reduced in efflux knockout strains (Figures S4A and S4B), we tested a therapeutic strategy that combined antibiotics with efflux pump inhibitors (EPIs). Phenylalanine arginyl β-naphthylamide (PAβN) and 1-(1-Naphthylmethyl) piperazine (NMP) are well studied inhibitors that can effectively block TolC-composed efflux pumps through competitive substrate export. When treating wild-type *E. coli* with carbenicillin, the addition of PAβN had little effect, but the addition of NMP greatly improved lethality. The persister number was reduced to less than 20% of the number surviving when carbenicillin was applied alone (Figure 5E). We further tested if such potentiating effect was observable with other antibiotics. Starting again from an initial concentration of 5 × 10^7^ cells/ml, the persister fraction in cells treated with cloxacillin (β-lactam) was ∼5 × 10^4^ cells/ml. The addition of PAβN and NMP reduced this number to ∼8 × 10^3^ cells/ml and ∼3 × 10^3^ cells/ml (Figure 5F), about 17% and 12% of the number that survived in the absence of inhibitors, respectively. The same effect was also observed in bacteria treated with nalidixic acid (quinolone), which produced ∼2 × 10^6^ cells/ml persisters. The addition of PAβN and NMP reduced this number to ∼2.5 × 10^4^ cells/ml and ∼5 × 10^4^ cells/ml (Figure 5G), about 0.5% and 3% of the number surviving without inhibitors, respectively. Those EPIs alone did not influence cell growth (Figure S4C). These results show that the combining antibiotics with EPIs offers therapeutic promise in the fight against bacterial persisters.

### Active Efflux Plays a Primary Role in the Drug Tolerance of Persister Cells

**Figure 6:**
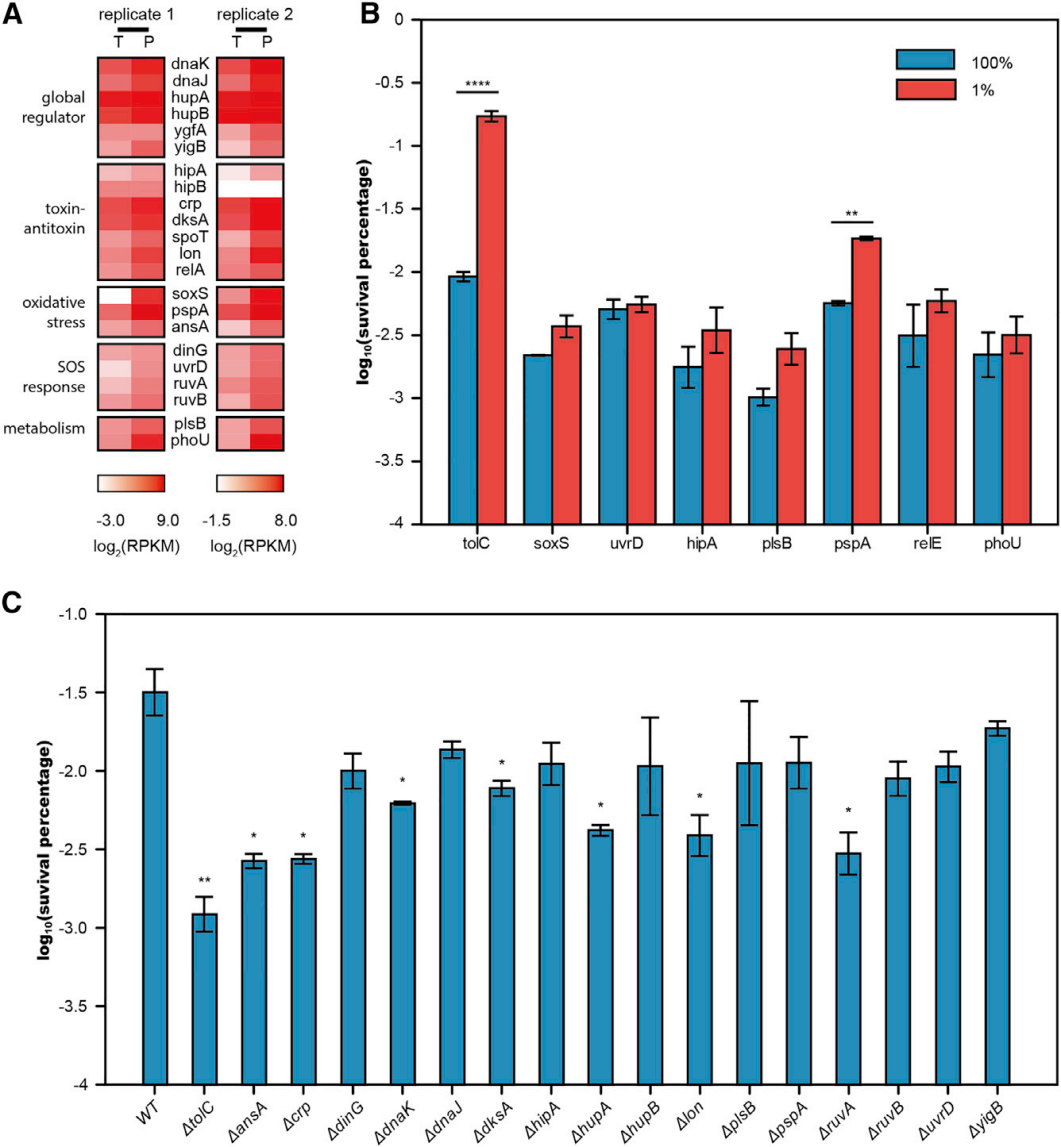
System-wide Comparison of the Contribution of Persistence Genes to Bacterial Drug Tolerance (A) Heatmap showing relative transcript abundance of persistence related genes in total cells and persister cells. Scale below the heatmap indicates log2-normalized transcript abundance relative to the mean expression level (T, total cells; P, persister cells). See also Table S1. (B) Bar plot representing cell survival rate (percentage log scale) after 4 hr carbenicillin treatment of two groups sorted by flow cytometer from fluorescently labeled *tolC*, *soxS*, *uvrD*, *hipA*, *plsB*, *pspA*, *relE*, and *phoU* strains, respectively. (C) Bar plot representing cell survival rate (in logarithm scale) after 4 hr carbenicillin treatment of knockout strains *ΔtolC*, *ΔansA*, *Δcrp*, *ΔdinG*, *ΔdnaK*, *ΔdnaJ*, *ΔdksA*, *ΔhipA*, *ΔhupA*, *ΔhupB*, *Δlon*, *ΔplsB*, *ΔpspA*, *ΔruvA*, *ΔruvB*, *ΔuvrD*, and *ΔyigB*. The bars indicate mean of at least three independent experiments; error bar indicates SD (^∗^p value < 0.1; ^∗∗^p value < 0.01; ^∗∗∗^p value < 0.001; ^∗∗∗∗^p value < 0.0001).

Further analysis of our transcriptome sequencing data identified several other groups of genes that showed differential expression between persister and total cells (Figure 6A; Table S1), implying multiple pathways that can be involved in persister formation. Global regulators that mediated downstream pathways leading to cell dormancy, including *dnaJ*, *dnaK*, *hupA*, *hupB*, *ygfA*, and *yigB* (Hansen et al., 2008), presented higher expression level in persister cells than in total cells. Toxin genes and their regulators from the TA modules *hipA*, *crp*, *dksA*, *spoT*, *lon*, and *relA* displayed upregulation in persister cells, whereas antitoxin gene *hipB* showed no changes between the two cell types (Balaban et al., 2004, Amato et al., 2013, Hansen et al., 2008, Maisonneuve et al., 2011, Maisonneuve et al., 2013). Oxidative stress response related genes, including *soxS* (Wu et al., 2012), *pspA* (Vega et al., 2012), and *ansA*, were also upregulated in persisters, as well SOS response genes *dinG*, *uvrD*, *ruvA*, and *ruvB* (Theodore et al., 2013). Finally, slow metabolism-related genes *plsB* (Spoering et al., 2006) and *phoU* (Li and Zhang 2007) were also highly expressed in persisters. The higher expression of these genes promotes a cell’s entry into the dormant state. However, unexpectedly, efflux pumps were actively working in those dormant cells, conferring upon them an increased capacity to tolerate antibiotic attack.

In order to rank the individual genes contributing to persistence, we constructed two libraries: a fluorescent protein EGFP fusion library and an ordinary knockout library covering reported genes associated with persistence. For the EGFP fusion library, we again used FACS to isolate cells with high fluorescence intensity (1%) and then performed the antibiotic sensitivity assay on these cells and compared that with the assay on the total population (100%). The results revealed that cells with higher expression levels of *tolC* showed the largest increase in bacterial persister enrichment (18.6-fold, p value = 0.00008) (Figure 6B). On the other hand, higher expression of other persistence related genes had less impact on persister enrichment (Figure 6B), of which *pspA* was the only gene that caused a significant increase in persister formation (3.2-fold, p value = 0.007). For the knockout library, we performed the antibiotic sensitivity assay to determine the effect of each knockout on bacterial survival rate. The strain carrying a deletion of *tolC* suffered the greatest reduction (29-fold) in persister formation following treatment with carbenicillin. Deletion of other individual persistence-related genes caused a lower reduction in persister formation (Figure 6C), including *ΔansA* (14-fold), *Δcrp* (14-fold), *ΔdnaK* (6-fold), *ΔdksA* (5-fold), *ΔhipA* (3-fold), *ΔhupA* (9-fold), *Δlon* (9-fold), and *ΔruvA* (11-fold). According to previous research (Maisonneuve et al., 2011), single deletion of *lon* led to a significant reduction in persister formation (similar to our results here), and the effect was comparable to that caused by deletion of *10 TA* genes together. Of note, our results show that deletion of *tolC* results in a 29-fold reduction in persister formation, a three-times further decrease when compared to that caused by deletion of *lon*.

The above results suggest that multiple mechanisms can lead to persistence, and while the contribution of each mechanism varies, the efflux system holds the most significant role in persister formation, at least in our experimental conditions. Our observations highlight the precise control behind persister formation. Bacterial persisters manage to combine two seemingly contradictory mechanisms to survive antibiotic attack: they slow down most physiological processes during dormancy and at the same time activate their efflux systems to remove intracellular antibiotics.

## Discussion

Gene expression is intrinsically stochastic. The variation in synthesis and breakdown of molecules in biochemical reactions that initiate and regulate transcription and translation gives rise to the heterogeneity in amount of protein expressed in a population of cells (Huang, 2009, Elowitz et al., 2002, Raj and van Oudenaarden, 2008). This was recently demonstrated by a high-throughput system-wide analysis (Taniguchi et al., 2010). In our experiments, we have observed that populations of bacteria have a heterogeneous distribution of efflux pumps due to stochastic gene expression. The subpopulation with the higher expression of efflux genes is able to pump more antibiotics outside the cell to reduce intracellular antibiotic accumulation and is thus more likely to persist. Our observation is consistent with a previous study (Allison et al., 2011a) in which the authors introduced metabolites to induce antibiotic uptake, eradicating bacterial persisters and implying that a lower antibiotic concentration inside the cell is key to being persistent.

### Pre-existing versus Induced Expression of *tolC* in Persister Cells

Whether this overexpression of efflux genes preempts drug treatment or is induced by antibiotic attack as a protective response in persisters remains inconclusive. Figure 4A shows that surviving persister cells belong to cells with higher fluorescent intensity of TolC-FIAsH, indicating such upregulation of efflux genes in persister cells may preexist. However, we cannot rule out the possibility that some of this higher expression was induced by drug treatment. Recent reports have found that indole signaling contributes to persister formation, possibly via upregulation of the efflux pumps through activation of the two-component systems BaeS-BaeR or CpxA-CpxR and the PspAB and OxyR pathways as a result of stress response (Vega et al., 2012, Hirakawa et al., 2005). Additionally, at a population level, indole molecules produced by highly resistant cells have been shown to provide protection to other more susceptible cells by activating their drug efflux pumps and oxidative-stress protective mechanisms (Lee et al., 2010). This facilitated the survival of the whole population. We believe this population effect may play a role in persister formation; however, the exact ratio between pre-existing and drug induced persisters deserves further investigation.

### Lack of Cooperation between Porin Genes and Efflux Genes

There are two ways to reduce intracellular antibiotic concentration: to lessen the membrane permeability by expressing fewer porin channels and to enhance efflux pump expression and activity. In our study, we proved that the expression of efflux genes was upregulated in persister cells. However, unexpectedly, we found that the porin gene *ompF*, which is critical for β-lactam antibiotic transportation, was also slightly upregulated in persister cells. Such increased expression of porin genes would amplify antibiotic entry and therefore lead to cell death, attenuating the chance of persisting. We do not understand the mechanism underlying this upregulation of *ompF* in persister cells, yet this further highlights the significant role of efflux pumps in bacterial persistence. It is possible that TolC protein might exert its effect on *ompF* promoter area or the terminal end of *ompF* gene to regulate gene expression (Misra and Reeves, 1987). Therefore, the higher expression of *ompF* in persiser cells might be a side-effect caused by upregulation of *tolC*, as previous work has shown that downregulation of *tolC* led to a reduction in *ompF* expression (Morona and Reeves, 1982).

### Use of Efflux Inhibitors in Eradicating Bacterial Persisters

To effectively treat chronic infectious diseases, specific anti-persistence therapies are required to eradicate persisters or to prevent their formation. Several genes that contribute to persister formation are current targets for drug design, including *hipBA*, *tisAB*, *phoU*, *edpA*, and *plsB etc*. (Fauvart et al., 2011).

EPIs can be combined with conventional antibiotics to reduce efflux pump-associated intrinsic resistance or acquired multidrug resistance (Zechini and Versace, 2009). For example, broad-spectrum EPI PAβN enhanced the lethality of levofloxacin against drug-resistant *Pseudomonas aeruginosa* (Lomovskaya et al., 2001), and another pump inhibitor-verapamil reduced macrophage-induced drug tolerance in *Mycobacterium marinum* (Adams et al., 2011). In our current study, we have demonstrated that EPIs work synergistically with different antibiotics to reduce persister formation. This further emphasized the need to match specific EPIs with antibiotics to treat persistent bacterial infections.

### Latent but Active: Enhanced Efflux Activity Facilitates Drug Tolerance in Dormant Bacterial Cells

Previously, cell dormancy was considered as the leading mechanism resulting in bacterial persistence (Balaban et al., 2004, Rotem et al., 2010). In these cells, antibiotics failed to eradicate bacterial cells due to dormant downstream pathways, though antibiotics still bound to their molecular targets. It has long been proposed that multiple mechanisms contribute to bacterial persistence (Lewis, 2010, Allison et al., 2011b), and previous studies have identified many dormant pathways and genes related to persistence. These include global regulators (Hansen et al., 2008) that lead to slower cell growth, toxin genes in TA modules (Balaban et al., 2004, Maisonneuve et al., 2011) that induce a dormant state, oxidative stress response pathways (Vega et al., 2012, Wu et al., 2012), SOS response pathways (Theodore et al., 2013), and metabolism pathways (Spoering et al., 2006, Li and Zhang, 2007). However, dormancy alone cannot explain persistence (Orman and Brynildsen, 2013b). We used transcriptome sequencing to identify upregulation of an additional group of efflux genes that contribute to persister formation and further established that cells with upregulated efflux genes showed elevated persistence while efflux knockout mutants showed attenuated persistence. Together, this suggests efflux pumps play a critical role in bacterial drug tolerance.

As demonstrated by our results, in addition to a “passive defense” via dormancy, bacterial persisters employ an “active defense” to pump antibiotics out and reduce intracellular drug concentration through enhanced efflux activity. Our findings indicate that while most biological processes in persisters are slowed down, the efflux systems, on the contrary, become more active and effectively promote antibiotic persistence. It is surprising to see that active efflux and dormancy coexist in many persister cells since dormancy seems to exclude active efflux. How bacterial cells control precisely which pathways to shutdown and which to activate, and whether dormancy and active efflux are intrinsically co-regulated, remains unknown. Our observation that persisters combine two contradictory mechanisms to survive antibiotic attack highlights the advanced network regulation endowed by natural selection, and also the challenges we face in eradicating these drug tolerant cells.

## Experimental Procedures

### Bacterial Strains and Plasmid Construction

Wild-type strain BW25113 and its *tolC* knockout strain (JW5503) were gifts from Yale Genetic Stock Center. The detailed information for the construction of *tolC* overexpression strain (Figure S5A), *tolC* rescued strain, 76RP strain (Figure S5B), and other strains are provided in Supplemental Experimental Procedures. Primers used are listed in Table S3.

### Antibiotic Killing and Persister Isolation

Frozen stocks of *E. coli* strains were diluted by 1:1,000 into LB medium and cultured overnight. The overnight culture was diluted in fresh LB to appropriate concentration (OD_600_ = 0.2), which was then split into two flasks. To one flask carbenicillin (150 μg/ml, Sigma) was added at a desired concentration to yield persisters. To the other flask the same volume of sterile Milli-Q (which was the solvent of carbenicillin solution) was added to yield total cells. The cultures were returned to the 37°C shaker for 4 hr. Then the cells were collected by centrifuging at 4000 × *g* for 5 min and washed with M9 minimal medium with 150 μg/ml carbenicillin three times to isolate unlysed persister cells and total cells. To confirm that the persisters we analyzed were from the second killing phase, the biphasic killing curves of each strain were shown in Figure S6.

### Fluorescent Microscopy

All the imaging work was performed on an inverted microscope (Nikon Eclipse *Ti*). The illumination was provided by different solid state lasers (Coherent), at 488nm for BOCILLIN and FlAsH, and 532nm for propidium iodide, respectively. The fluorescent signal of cells was collected by an EMCCD camera (Photometrics Evolve 512). The appropriate filter sets were selected for each fluorophore according to its spectrum. Image analysis was done by ImageJ software (Fiji). Detailed information is described in Supplemental Experimental Procedures.

### Flow Cytometry Analysis and Cell Sorting

All samples were measured on a BD FACSAria III flow cytometer. FIAsH labeling 76RP strain or chromosomal *geneX*-*egfp* translational fusion strain in the stationary phase was resuspended in sterile 1XPBS, which was also used as sheath fluid in flow cytometer. Microorganisms were identified by FSC (forward scatter) and SSC (side scatter) parameters. Cells were sorted into three groups based on their fluorescence intensity (488-nm excitation with 530/30-nm-band-pass filter) using a 70 μm nozzle. Approximately 100,000 cells were collected in each group. The results were analyzed by FlowJo V10 software (Treestar, Inc.).

### Antibiotic Sensitivity Assay

The overnight cultures of *E. coli* strains were diluted by 1:20 into fresh LB with antibiotic carbenicillin (final concentration of 0 μg/ml, 20 μg/ml, 40 μg/ml, 80 μg/ml, and 160 μg/ml, respectively). Then the culture was returned to the 37°C shaker for another 4 hr. Samples were withdrawn and appropriately diluted in LB medium and spotted on an LB agar plate for overnight culture at 37°C. Colony counting was performed the next day. For the inhibitor assay, PAβN (sigma) or NMP (sigma) at a final concentration of 100 μM was added into medium at the same time points with antibiotics.

## Author Contributions

Y.P., Z.Z., and Y.L. designed the study; carried out experiments; and analyzed the data; J.Z., Y. Zhao, and Y. Zhu performed mutagenesis experiments; Q.M., H.G., Y.S., Y.K., and M.A.B.B. analyzed the sequencing data and performed related bioinformatics analyses; F.B. and X.S.X. designed and supervised the study; Y.P., Z.Z., Y.L., and F.B. wrote the manuscript with contributions from H.C. and M.A.B.B.

## Accession Numbers

Sequencing data have been deposited at the NCBI sequence read archive under accession number SRA: SRP072433 (http://www.ncbi.nlm.nih.gov/sra/SRP072433).

## Supplemental Information

Document S1. Figures S1–S6 and Supplemental Experimental Procedures

Table S1. RNA-Seq and Differential Expression Profile Analysis

Table S2. Whole-Genome Sequencing of Persister and Untreated Total Cells

Table S3. Primer Pairs Used in this Study

Movie S1. Time-Lapse Microscopy Showing the Persister Cells Accumulating Less Cellular Antibiotics

Movie S2. Bacteria Efflux Rate Measurement by Single-Cell Level Time-Lapse Microscopy

Movie S3. Time-Lapse Microscopy Showing that Persister Cells Expressing a High Level of TolC Protein

Document S2. Article plus Supplemental Information
