# Supplementary material for: Enhanced Efflux Activity Facilitates Drug Tolerance in Dormant Bacterial Cells
Source: Mol Cell. 2016 Apr 21;62(2):284–94. doi: 10.1016/j.molcel.2016.03.035 (PMC4850422; doi:10.1016/j.molcel.2016.03.035)
Supplement: Document S1. Figures S1–S6 and Supplemental Experimental Procedures [file mmc1.pdf]

Molecular Cell, Volume *62*

Supplemental Information

Enhanced Efflux Activity Facilitates

Drug Tolerance in Dormant Bacterial Cells

Yingying Pu, Zhilun Zhao, Yingxing Li, Jin Zou, Qi Ma, Yanna Zhao, Yuehua Ke, Yun Zhu, Huiyi Chen, Matthew A.B. Baker, Hao Ge, Yujie Sun, Xiaoliang Sunney Xie, and Fan Bai

Supplemental Figures and Legends

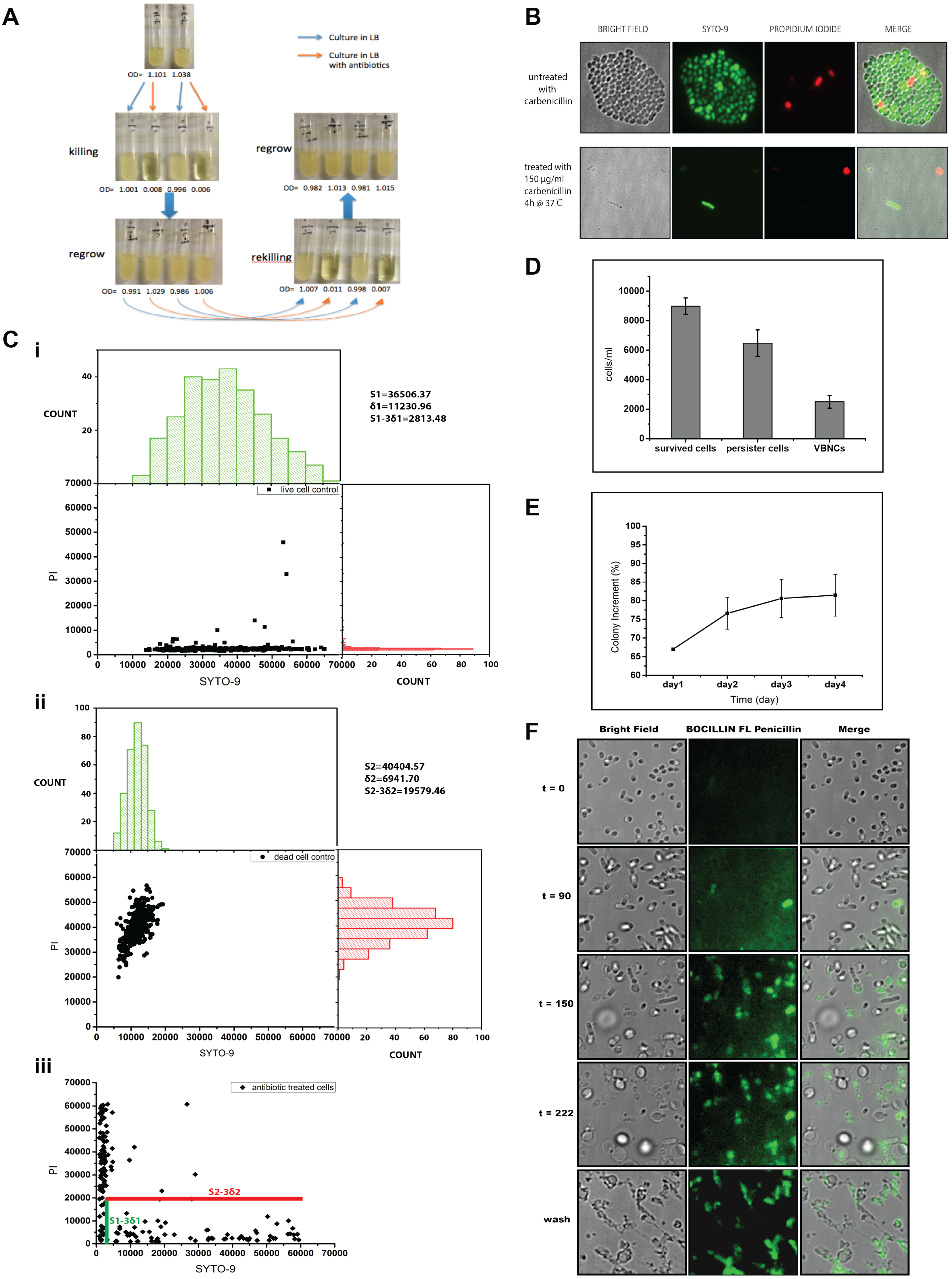

Figure S1. Related to Figure 1

(A) Drug tolerance and regrowth assay. In order to verify that what we have studied is persistence rather than resistance, we tested the drug tolerance and regrowth properties of the cells. The overnight culture of BW25113 strain was diluted 1:20 into fresh LB, which was then split into two tubes. For one tube 150 μg/ml carbenicillin (Sigma) was added and the other without antibiotic was set as control, and the culture was returned to the 37°C shaker for 4 hours to get drug tolerant cells. These cells were collected by centrifugation and re-suspended in fresh LB to resume growth. The killing and regrowth procedures were repeated again on the regrown cultures to ensure that persistence was transient and reversible. Two biological replicates were tested in this assay. **(B-D)** Persister cells are the main cell type in the survival population after carbenicillin treatment. Firstly we obtained the biphasic killing curves to make sure the survival cells we isolated were from the second killing phase (Figure S6). Then the remaining cells were collected with gentle centrifugation (4000 g, 3min). To quantify the number of live cells in the remaining population, cells were washed three times with staining buffer and stained with LIVE/DEAD BacLight Bacterial Viability Kits (Invitrogen). The washing and staining processes were all done in the presence of the same concentration of antibiotics used in the killing process to ensure the persistent nature of these cells. After staining, the remaining cells were divided equally into two tubes. One tube was used for plating and colony-counting assay to enumerate the number of persister cells on the next day. **(B)** The other tube was examined under a microscope to enumerate the total number of survived cells after antibiotic treatment. For this purpose, we evenly smeared a certain volume of cells on a 14mm*24mm rectangular gel pad (4% w/v of low melting agar) and waited for semi-dryness. Then the gel pad was covered by a coverslip and observed under a microscope. We randomly counted the number of cells in 20 image fields (0.493 mm * 0.493 mm/image field) with an automatic program. A live cell was defined as SYTO-9 positive and propidium iodide negative with intact cell shape. **(C)** Total survival cells quantification process. According to a previous protocol (Orman and Brynildsen, 2013), we used SYTO-9/PI staining to identify live cells from the remaining population after carbenicillin treatment. i, Red(PI) vs. Green (SYTO-9) fluorescence distribution of the live cell control (exponentially growing cells stained with SYTO-9 and PI). Fluorescence distribution of SYTO-9 of the live cell control was fitted with a Gaussian distribution (mean: S1; standard deviation: δ1); ii, Red (PI) vs. Green (SYTO-9) fluorescence distribution of the dead cell control (70% ethanol treated cells stained with SYTO-9 and PI). Fluorescence distribution of PI of the dead cell control was fitted with a Gaussian distribution (mean: S2; standard deviation: δ2); iii, Identification of total survival cells in the remaining population after four hours of 150ug/ml carbenicillin treatment. A live cell is defined as SYTO-9 signal above (S1- 3δ1, green line), PI signal below (S2- 3δ2, red line) and with intact cell shape. The population below (S1- 3δ1, green line) and below (S2- 3δ2, red line) is assumed to be cell capsids without nucleic acid. **(D)** By the method described above survived cells were counted as ~ 9000 cells/ml. Meanwhile, persister cells counted from colony counting assay equaled ~ 6000 cells/ml. If we assume the total survival cells consist of persister cells and viable but non-culturable cells (VBNCs), then the number of VBNCs can be estimated as ~3000 cells/ml. **(E)** The number of persisters with respect to incubation time, suggesting that VBNCs can switch to persister cells under suitable growth conditions. **(F)** Separate bright field and fluorescent images of the merged images shown in Figure 1D and Movie S1. From t = 0 to t = 222 is the killing process with a combination of 150 μg/ml carbenicillin and 20 μg/ml BOCILLIN FL Penicillin. The fresh medium without antibiotic was injected at t = 228 to allow persister cells to regrow (t = time in min). The bars indicate mean of at least three independent experiments; error bar indicates SD.

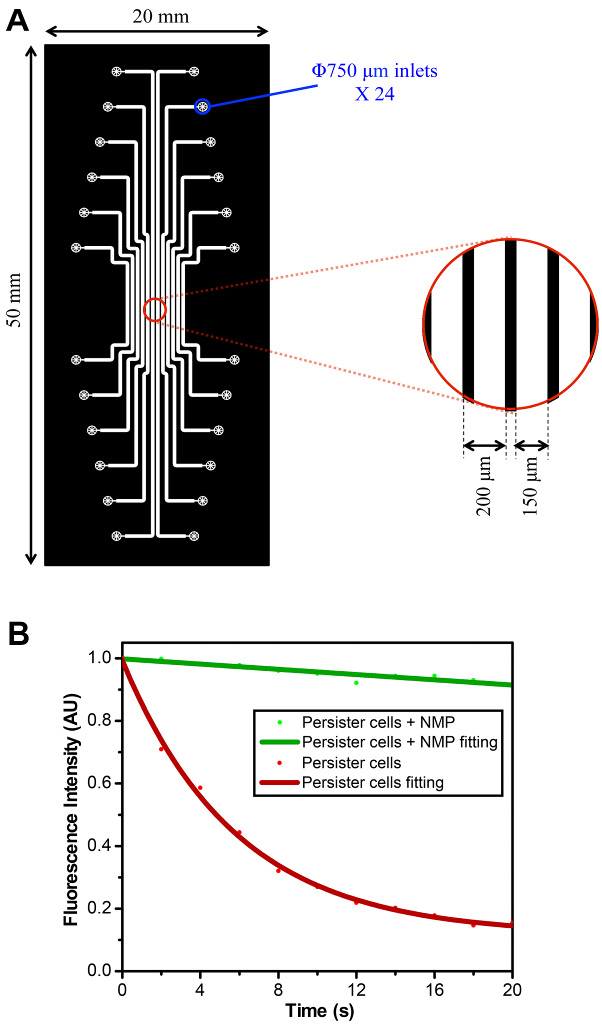

Figure S2. Related to Figure 2

(A) Microfluidic device design. 12 independent microfluidic channels were fabricated as an integrated pattern within a 20mm × 50mm area. The circles at the ends of channels are the inlet/outlet of the channel and holes with Φ0.75mm are punched through PDMS replicates at the same locations. The width of channel is 150 µm and the height is approximately 20 µm. **(B)** Intracellular fluorescent intensity decay after removing antibiotic in the medium is well fit by a single exponential function. The fast decay rate of fluorescent intensity in a persister cell (red) was inhibited by 1-(1-Naphthylmethyl) piperazine (NMP, 100µM) (green). Our results demonstrate that when treating with efflux inhibitor, the fluorescence stabilizes in the persisters, indicating that diffusion was not responsible for the loss of fluorescence in that sample.

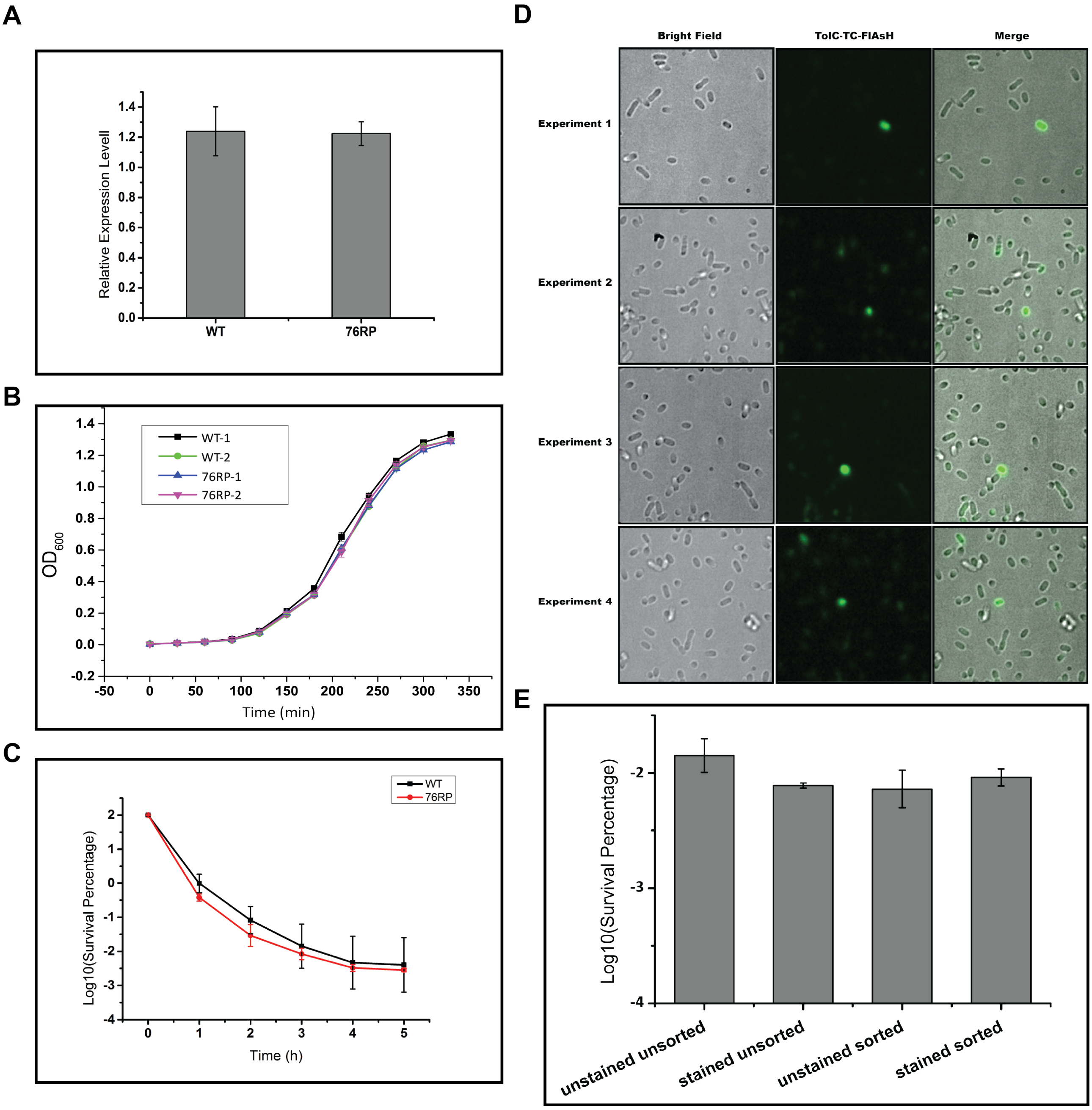

Figure S3. Related to Figure 4

(A-C) Comparison of 76RP strain and wild type strain in **(A)** *tolC* expression level measured by RT-qPCR. **(B)** growth curves. **(C)** killing curves under antibiotic treatment. **(D)** Separate bright field and fluorescent images of the merged images shown in Figure 4B and Movie S3. **(E)** Survival rates of stationary-phase cells of 76RP strain after carbenicillin treatment are not affected either by FlAsH staining or cell sorting. The bars indicate mean of at least three independent experiments; error bar indicates SD.

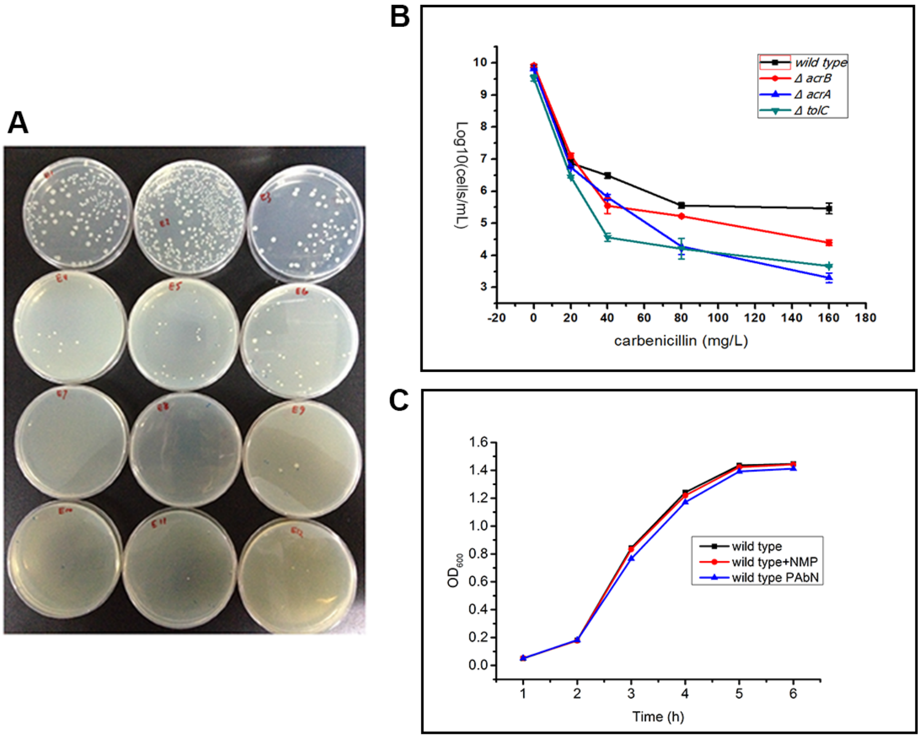

Figure S4. Related to Figure 5

(A-B) Correlation between bacterial persister formation frequency and gene expression of efflux pump systems. **(A)** The antibiotic susceptible measurement of *E. coli* wild type, *ΔacrA, ΔacrB* and *ΔtolC* strains under 160μg/ml carbenicillin treatment. The collected persister cells from each strain were diluted in the same volume of fresh LB and plated on LB agar for overnight culture. The colony counting was performed the next day. Three biological replicates were prepared for each strain. **(B)** Persister formation frequency of the four strains under antibiotic treatment of gradient concentrations. **(C)** Growth rate of wild type strain with or without efflux inhibitors NMP (100μM) and PAβN (100μM). Our results demonstrate that the pump inhibitors alone do not influence cell growth. The bars indicate mean of at least three independent experiments; error bar indicates SD.

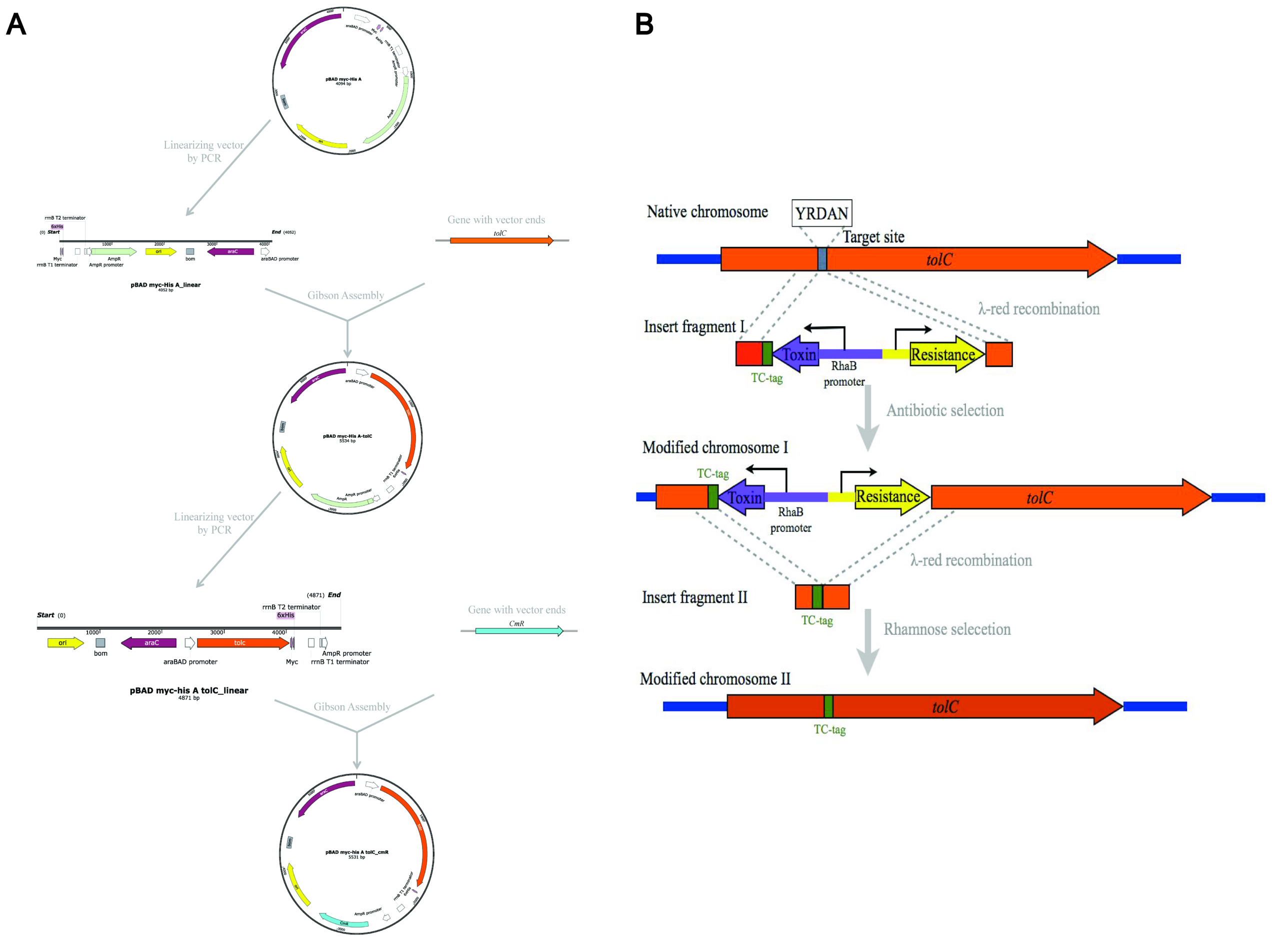

Figure S5. Construction of Strains Used in our Study, Related to Experimental Procedure

(A) Scheme of pBAD::myc-his_*tolC*_CmR construction. The PCR product of *tolC* with vector overlapping sequence was amplified from BW25113 and inserted into pBAD vector by *in vitro* Gibson Assembly method. The ampicillin resistant gene was replaced with chloramphenicol resistance gene by the same method. **(B)** Scheme of construction of TC tagged *tolC* 76RP strain. 76th~81th amino acids (YRDAN) of TolC protein, part of the linker between TolC S1 and S2 α-helixes, were firstly replaced with TC-Toxin-CmR cassette by λ-red recombination and then selected with chloramphenicol. The Toxin-CmR sequence was knocked out by another round of λ-red recombination with fusion fragment *tolC*-TC and selected with Rhamnose that would activate toxin expression.

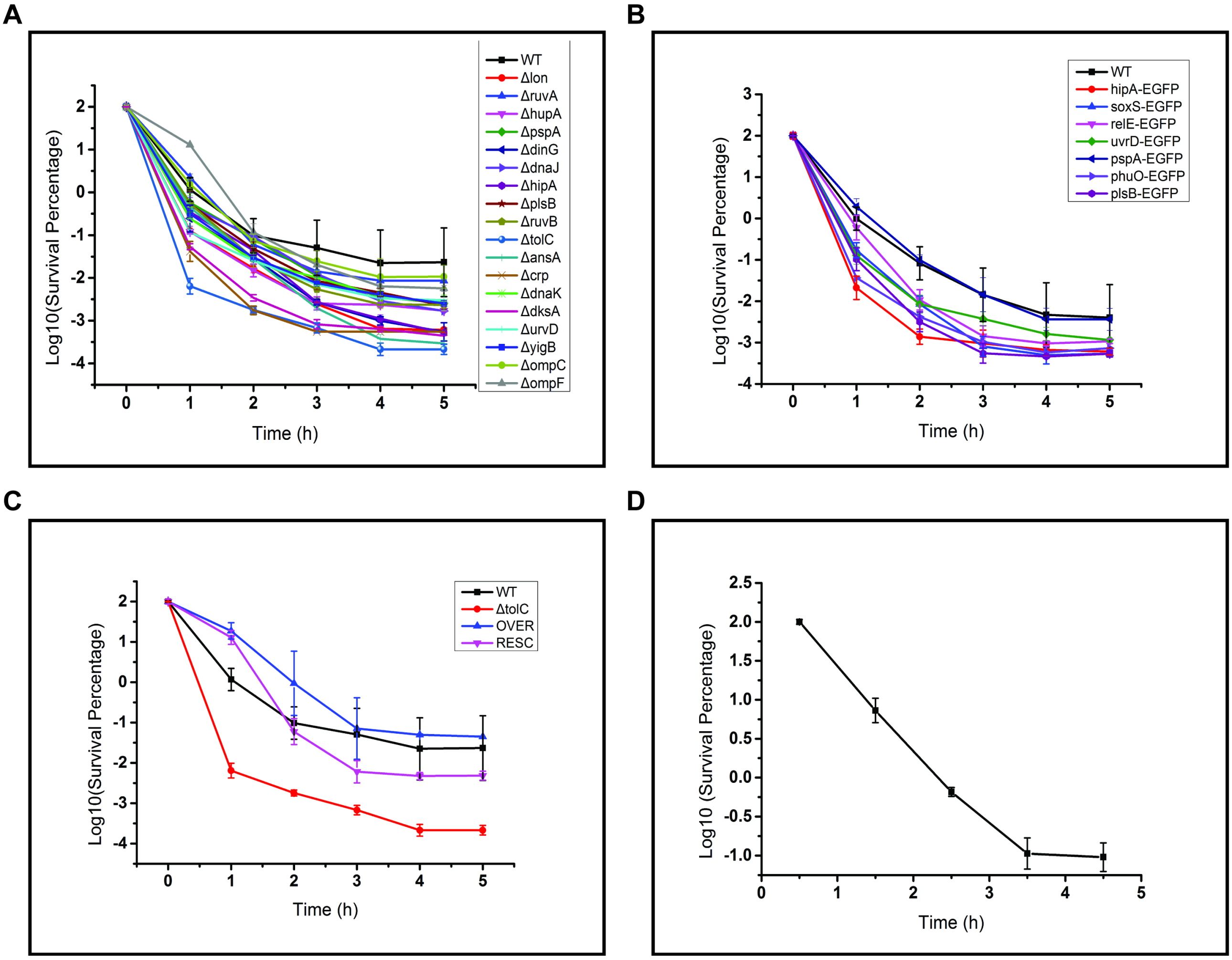

Figure S6. Biphasic Killing Curves of Strains Used in our Study, Related to Experimental Procedure
(A) Biphasic killing curves of all the knockout strains used in Figure 6C, confirming that the persisters we analyzed were from the second killing phase. (To note, *ΔompC* and *ΔompF* strains have lower survival rate than wild-type, although they show less antibiotic accumulation. This might because deletion of *ompC* or *ompF* influences other pathways that are critical to bacterial survival. Since the expression levels of *ompC* and *ompF* were not significantly different between persisters and total cells, their role in persister formation is not the main focus of this work.) **(B)** Biphasic killing curves of all the EGFP labeled strains used in Figure 6B, confirming that the persisters we analyzed were from the second killing phase. **(C)** Biphasic killing curves of *tolC* knockout strain, *tolC* overexpression strain and *tolC* rescued strain used in Figure 5A, 5B, 5C and 5D, confirming that the persisters we analyzed were from the second killing phase. **(D)** Biphasic killing curves of cells in the temperature control chamber on the microscope used in our time-lapse movies. This confirmed that the persisters we analyzed were from the second killing phase before we removed antibiotic and watched their re-growth. The carbenicillin concentration was 150μg/mL in determining the surviving curves. The bars indicate mean of at least three independent experiments; error bar indicates SD.

Supplemental Movies and Legends

Movie S1. Time-lapse Microscopy Showing the Persister Cells Accumulating Less Cellular Antibiotics, Related to Figure 1.
Total cells from stationary phase of BW25113 were treated with 150 µg/ml of carbenicillin and 20 µg/ml BOCILLIN in medium (90% (v/v) M9 + 10% (v/v) LB medium + 0.15% (w/v) methylcellulose) for 222 min. The bright field and fluorescent images were acquired every 6 min automatically. Then fresh medium (90% (v/v) M9 + 10% (v/v) LB medium + 5% (w/v) methylcellulose) was injected to remove antibiotic at t = 228 min, which would allow survived cells to resume growth. Then the bright field images were acquired every 6 min automatically. This whole process was performed at 37°C.

Movie S2. Bacteria Efflux Rate Measurement by Single-cell Level Time-lapse Microscopy, Related to Figure 2.
Untreated total cells and persisters from wild type *E. coli* stained with BOCILLIN were immobilized in a poly-L-lysine coated PDMS microfluidic device. The imaging was begun as soon as the antibiotic containing medium was washed away and replaced by fresh M9 minimal medium (t = 0s) and the imaging process lasted for 2 min. Imaging conditions: exposure time = 50ms; interval = 2s; light intensity (λ = 488nm, I = 20mW/cm^2). Propidium iodide was used in order to distinguish dead cells from persisters.

Movie S3. Time-lapse Microscopy Showing that Persister Cells Expressing a High Level of TolC Protein, Related to Figure 4.
Total cells from stationary phase of 76RP stained with FlAsH were treated with 150 µg/ml of carbenicillin in medium (90% (v/v) M9 + 10% (v/v) LB medium + 0.15% (w/v) methylcellulose) for 210 min. Then fresh medium (90% (v/v) M9 + 10% (v/v) LB medium + 5% (w/v) methylcellulose) was injected to remove antibiotics at t = 210 min, which would allow survived cells to resume growth. The first image is the merge of bright field and fluorescent channels showing the level of TolC expression at t = 0 min. Then the bright field images were acquired every 5 min automatically. The latent time before regrowth varies from cell to cell. The whole process was performed at 37°C. Propidium iodide was used in order to distinguish dead cells from live cells.

Supplemental Tables and Legends

Table S1. RNA-seq and Differential Expression Profile Analysis, Related to Figure 3 and 6.
We compared transcriptome profiles of persister and total cells of wild type *E.coli* to determine changes in gene expression. Overnight culture was diluted 1:15 into 200ml LB or M9 medium and split into two flasks. One flask was then treated with 150 μg/ml carbencillin to get persister cells and the other was set as untreated control to get total cells. After 4 hours culture at 37°C, RNA was isolated and processed to transcriptome sequencing. The resulting sequencing data were further normalized to get gene expression RPKM value which allow the analysis of changes in gene expression between persister and total cells. We chose significantly changed genes by selecting those with log2 (persister/total) >1 and p value < 0.005. Two data sets were merged to obtain a common set of genes up- or down-regulated in two independent biological replicates. As showed in Table S1, 298 genes were up-regulated and 33 genes were down-regulated in their expression in persisters in both experiments.

Table S2. Whole Genome Sequencing of Persister and Untreated Total Cells, Related to Figure 3.
Genetic mutations in upstream regulators could also result in increased expression of efflux-associated genes, which has been well documented in bacterial antibiotic resistance (Poole, 2002; Wiercinska et al., 2015; Wren et al., 2015). In order to further confirm that there were no mutations occurring on efflux genes and their regulatory pathways, we performed whole genome sequencing on both total cells and persisters. As shown in this table, no mutation was found in efflux genes or in the related regulatory pathway in all three biological replicates of persisters. Moreover, no mutation was common to all three repeats, indicating mutations between persisters and total cells are random.

Table S3. Primer Pairs Used in this Study, Related to Experimental Procedure.

Supplemental Experimental Procedures

Bacterial strains and plasmid construction

pBAD::*tolC* plasmid was transformed by electroporation into BW25113 and JW5503 to generate the *tolC* overexpression strain and rescue strain, respectively. The *tolC* PCR product was amplified from BW25113 and inserted into pBAD/Myc-His A vector at Nco I and Hind III sites by the *in vitro* Gibson Assembly method. The ampicillin resistant gene was replaced with chloramphenicol (CAM) resistance cassette by the same method (Figure S5A). Luria-Bertani (LB) broth and LB agar media were used for culturing.

76RP strain was obtained by replacing TolC protein 76th~80th amino acids (YRDAN) that are part of the linker between TolC S1 and S2 α-helix with TC-Tag (CCPGCC) (Figure S5B). TolC expression level (Figure S3A), growth curve (Figure S3B) and killing curve (Figure S3C) showed the replacement did not compromise the functionality of TolC protein. Strains containing chromosomal *geneX-egfp* translational fusion or single *geneX* knockout mutants were constructed by λRed-mediated gene replacement (Datsenko and Wanner, 2000).

Fluorescent Microscopy

For intracellular fluorescent antibiotic accumulation measurement, the LB-cultured total and persister cells were collected, washed three times with M9 minimal medium and incubated with fluorescent antibiotic BOCILLIN for 30 min at 37°C with gentle shaking. Additional experiments proved that the staining time between 30~60 min would not significantly affect the result. The cells were then used directly for epifluorescence microscopy or total internal reflection fluorescence microscopy at the interface between the coverslip and the M9 minimal growth medium containing methyl-cellulose and BOCILLIN. For efflux rate measurement, we used PDMS microfluidics coated with poly-L-lysine for imaging. After being injected into channels, the cells were incubated for about 10 minutes at room temperature for stable resting. We started time-lapse epifluorescence imaging as soon as the medium containing antibiotics was washed away by new M9 minimal medium. For FlAsH staining, after being harvested and washed as described above, the bacteria were re-suspended in FlAsH-EDT_2 (Invitrogen) diluted to 20μM into M9 minimal, and were incubated at RT for 90 min. Then the bacteria were washed twice with BAL wash buffer supplied by Invitrogen. Propidium iodide from Invitrogen LIVE/DEAD staining kit was then used to re-suspend the bacteria in order to distinguish dead cells from surviving cells. The camera exposure time and gain was adjusted to prevent over-saturated image acquisition.

Image processing

Image analysis was done by ImageJ software (Fiji). Cell contours were constructed from bright field images. The background image and cell auto-fluorescence was subtracted from the fluorescent image. The integrated fluorescence intensity of the entire cell area was measured for each cell and normalized by cell area. The extracted data from time-lapse images was analyzed in Matlab (R2011a, Mathworks) using custom data processing script. For efflux rate assay, the cell intensity traces were fitted by single exponential function; the parameters were statistically analyzed and verified by *Student's t*-test.

Microfabrication

The microfluidic pattern (Figure S2A) was designed on AutoCAD 2004 (Autodesk Inc.) and output into a photomask film using a commercial photoploting service with a resolution of 20,000dpi. Photolithography was used to produce pattern on test-grade silicon wafer (University wafer). The pattern was developed by exposing UV-light to the wafer which was coated with a UV-curable exposy (SU8-2025, Micro-Chem) of 25 μm thick. Softlithography was used to produce microfluidic platform. Poly-dimethylsiloxane (PDMS), a low-cast, optically transparent silicon elastomer, was molded on the fabricated wafer by curing at 80°C for 20 minutes. Holes of Φ750 μm were punched through the inlet/outlet positions of the replicated PDMS sheet. The PDMS sheet was bonded to a coverslip (0.17 mm thick, 48 x 60 mm, FISHER) by treatment with an oxygen plasma cleaner.

RNA isolation and RNA-seq

The cells were lysed by lysozyme (400μg/ml, Ready-Lyse™ Lysozyme Solution, Epicenter) with addition of RNase inhibitor (Invitrogen) for 10min at room temperature. Total RNA was then extracted by using RNeasy Mini Kit (Qiagen). Ribosomal RNA was depleted by RiboMinus Transcriptome Isolation Kit (Invitrogen) to get mRNA that was then cleaned up using RNeasy Mini Spin columns (Qiagen). The resulting transcriptome RNA was used for RNA-seq library construction using NEBNext mRNA Library Prep Reagent set for Illumina (NEB) according to manufacturer's protocol. Briefly, mRNA was fragmented to desired length and reverse transcripted into first strand cDNA. The single strand cDNA was used for double strand DNA synthesis followed by end repair, dA-tailing, adaptor ligation and PCR amplification. Before sequencing, the library was examined by length determination and quantitative PCR certification. These constructed libraries were then sequenced by Illunima Hiseq 2000 platform by paired-end chemistry.

DNA isolation and genome sequencing

The cells were lysed by lysozyme (400μg/ml, Ready-Lyse™ Lysozyme Solution, Epicenter) for 15 min at room temperature. DNA was then extracted by using DNeasy Blood & Tissue Kit (Qiagen). The genome sequencing library was constructed by using NEBNext DNA Library Prep Master Mix for Illumina (NEB, E6040). Briefly, genome DNA was fragmented into desired length, followed by end repair, dA-tailing, adaptor ligation and PCR amplification. Before sequencing, the library was examined by length determination and quantitative PCR certification. The constructed DNA libraries were then sequenced by Illunima Hiseq 2000 platform by paired-end chemistry.

Sequencing data analysis

DNA-seq reads were aligned to *Escherichia coli* reference NC_012971.2 (http://www.ncbi.nlm.nih.gov/assembly/GCF_000022665.1/) using the Burrows–Wheeler Aligner. Sorting of aligned reads, duplicate removal and detection of variations were done with Samtools 0.1.18 . The functional effect of variants was annotated with SNPEFF 3.0 . SNVs/INDELs were called for variations that presented in persister cells but not in the matched total cells. Differential gene expression analysis of RNA-seq was done with TopHat 2.0.12 and Cufflinks 2.2.1.

Reverse transcription and Quantitative PCR

The total RNA was extracted as described above. Reverse transcription was performed to yield cDNA by using SuperScript® III Reverse Transcriptase (Invitrogen) with random primers following standard protocol. Target gene expression level was measured by quantitative PCR with *mreB* as reference gene by using Brilliant II SYBR Green QPCR Master Kit (Agilent Technologies) on 7500 Real Time PCR System (Applied Biosystem).

Supplemental References

Datsenko, K.A., and Wanner, B.L. (2000). One-step inactivation of chromosomal genes in Escherichia coli K-12 using PCR products. Proc. Natl. Acad. Sci. USA *97*, 6640–6645.

Poole, K. (2002). Outer membranes and efflux: the path to multidrug resistance in Gram-negative bacteria. Current pharmaceutical biotechnology 3, 77-98.

Wiercinska, O., Chojecka, A., Kanclerski, K., Rohm-Rodowald, E., and Jakimiak, B. (2015). Significance of efflux pumps in multidrug resistance of Gram-negative bacteria. Medycyna doswiadczalna i mikrobiologia 67, 55-62.

Wren, M.S., Ganguli, K., Paridington, P.E., Dimitrijevic, M., McMahon, B.H., Schweizer, H., and Gupta, G. (2015). 58 Multi-drug resistance efflux pumps in bacteria: how they work? Journal of biomolecular structure & dynamics 33 Suppl 1, 39.
