# Supplementary figures and images for: Enhanced Efflux Activity Facilitates Drug Tolerance in Dormant Bacterial Cells
Source: Mol Cell. 2016 Apr 21;62(2):284–94. doi: 10.1016/j.molcel.2016.03.035 (PMC4850422; doi:10.1016/j.molcel.2016.03.035)

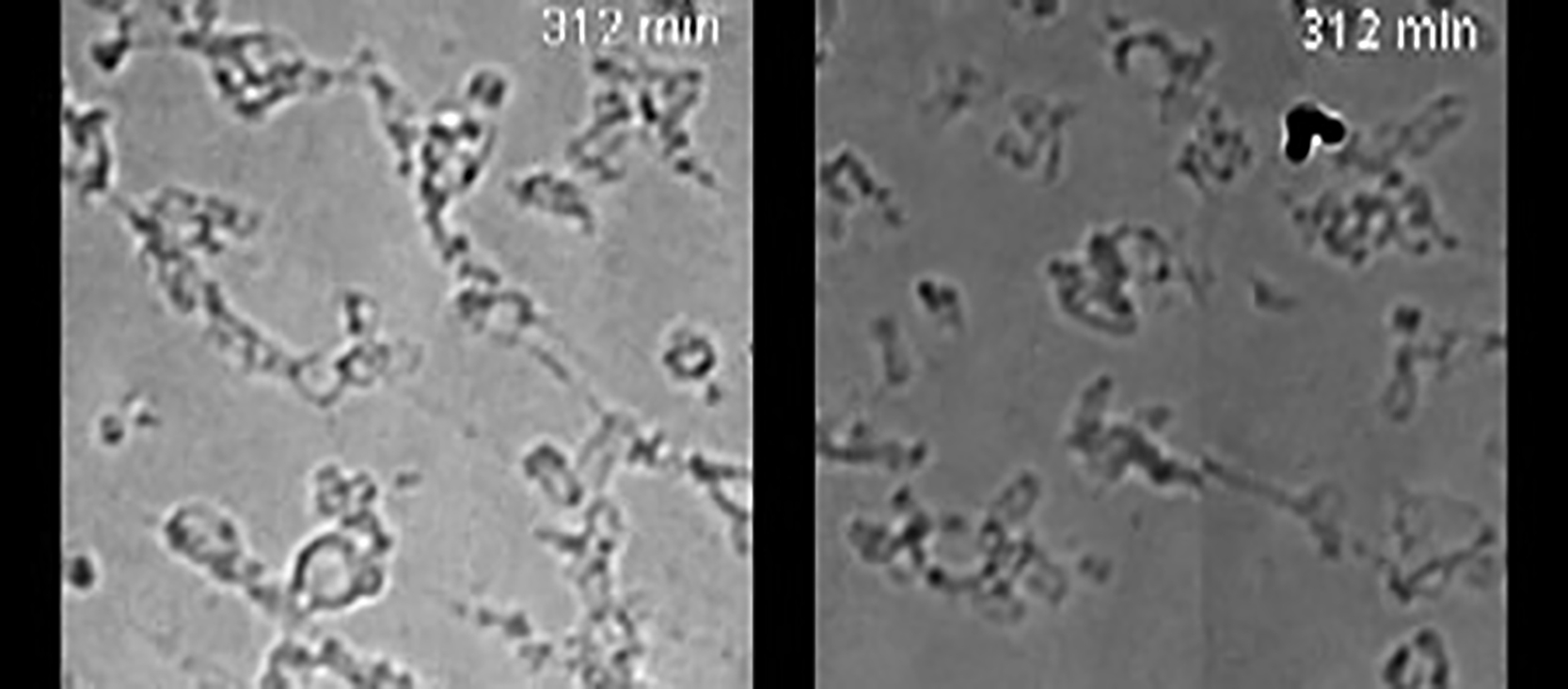

Supplement: Movie S1. Time-Lapse Microscopy Showing the Persister Cells Accumulating Less Cellular Antibiotics [file mmc5.jpg]

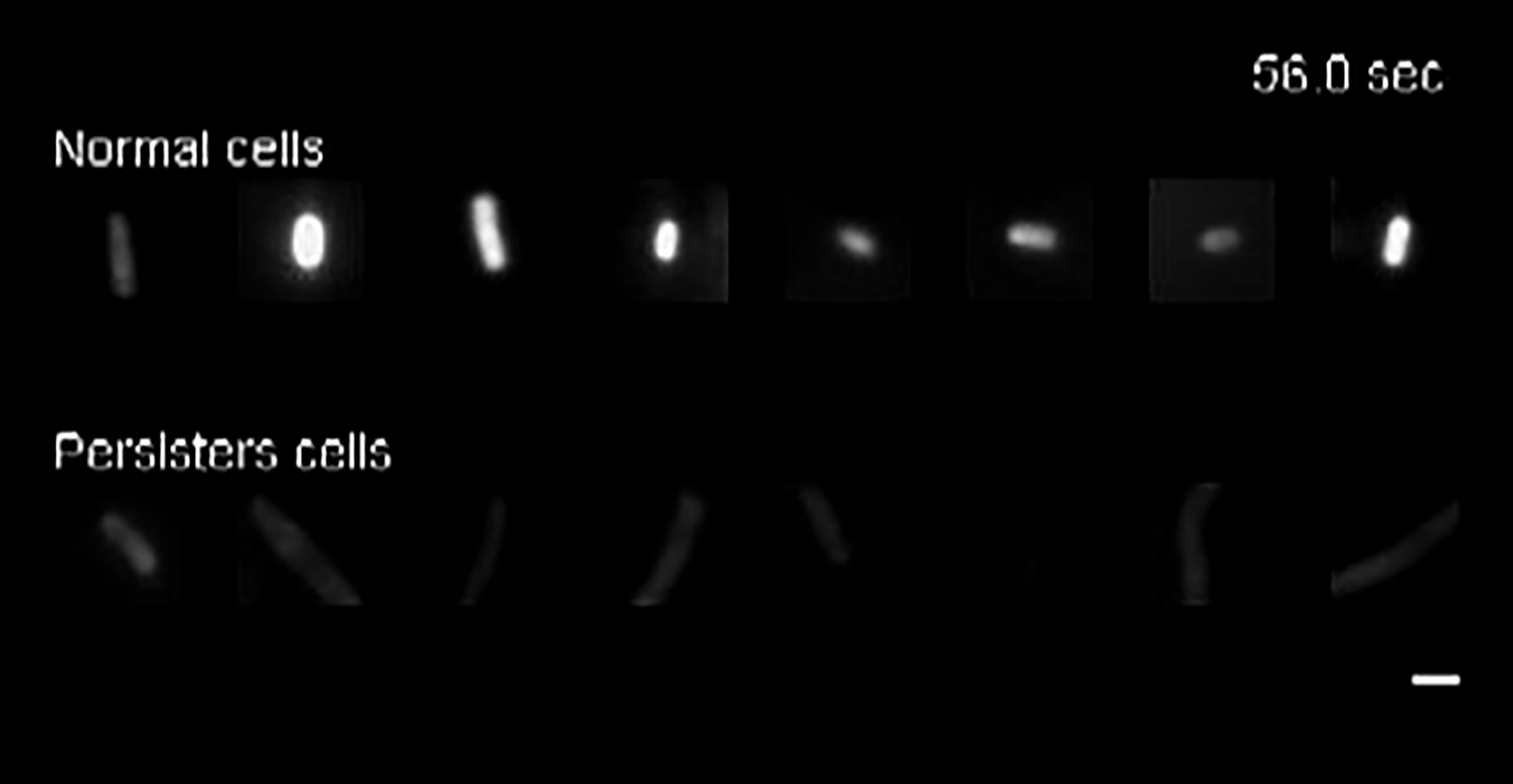

Supplement: Movie S2. Bacteria Efflux Rate Measurement by Single-Cell Level Time-Lapse Microscopy [file mmc6.jpg]

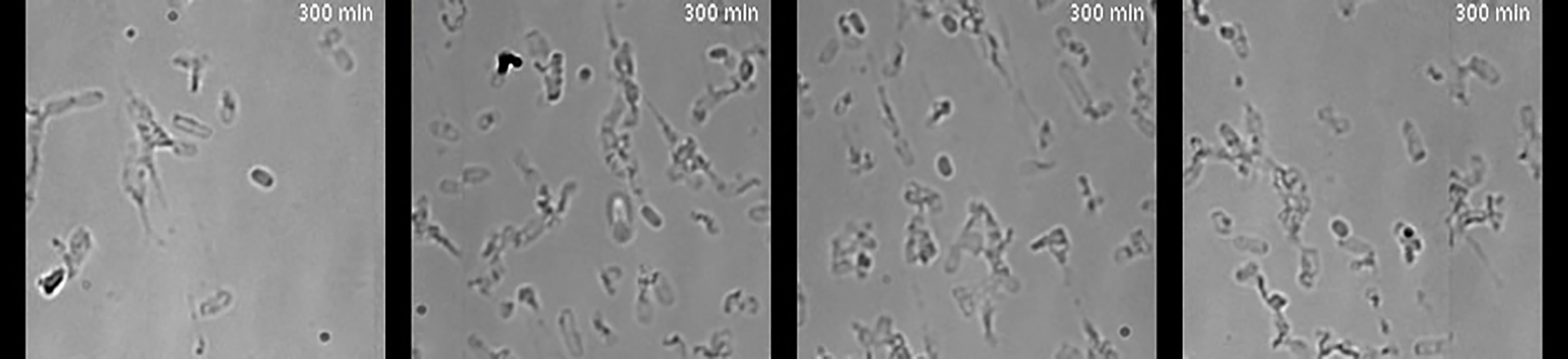

Supplement: Movie S3. Time-Lapse Microscopy Showing that Persister Cells Expressing a High Level of TolC Protein [file mmc7.jpg]
