# Supplementary material for: Enhanced Efflux Activity Facilitates Drug Tolerance in Dormant Bacterial Cells
Source: Mol Cell. 2016 Apr 21;62(2):284–94. doi: 10.1016/j.molcel.2016.03.035 (PMC4850422; doi:10.1016/j.molcel.2016.03.035)
Supplement: Document S2. Article plus Supplemental Information [file mmc8.pdf]

Molecular Cell

Article

Enhanced Efflux Activity Facilitates Drug Tolerance in Dormant Bacterial Cells

Graphical Abstract

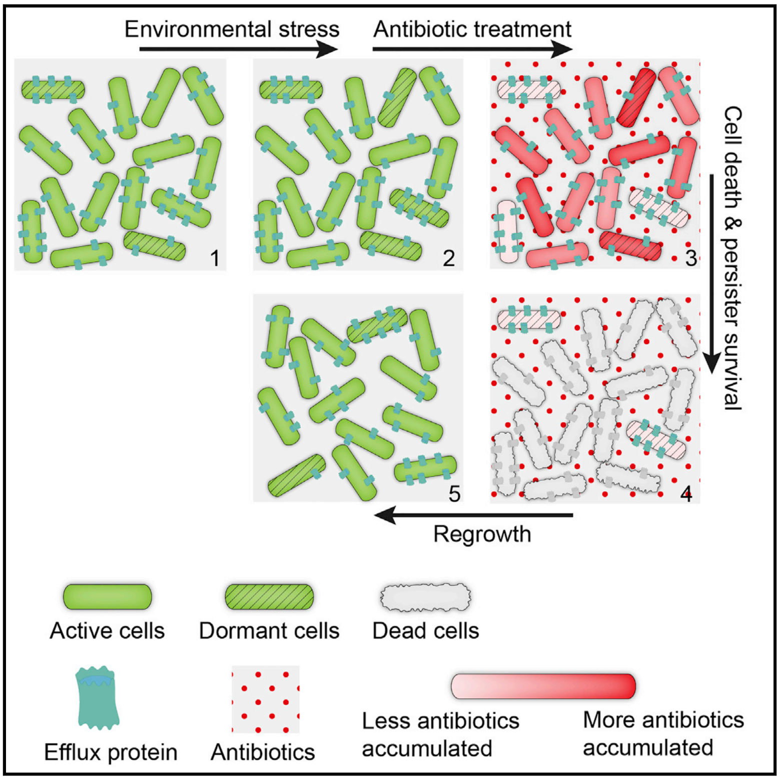

Authors

Yingying Pu, Zhilun Zhao, Yingxing Li, ..., Yujie Sun, Xiaoliang Sunney Xie, Fan Bai

Correspondence

xie@chemistry.harvard.edu (X.S.X.), fbai@pku.edu.cn (F.B.)

In Brief

By using in vivo fluorescent imaging and next-generation sequencing, Pu et al. demonstrate that bacterial persisters exhibit enhanced efflux activity in addition to physiological quiescence, a double insurance strategy to ensure its survival under antibiotic attack.

Highlights

- Persisters accumulate fewer antibiotics as a direct result of increased efflux rate
- Persisters show higher expression of efflux-associated genes
- High expression of *tolC* is critical to promote persister formation
- Persisters combine active efflux and passive dormancy to survive antibiotic attack

Pu et al., 2016, Molecular Cell *62*, 284–294
April 21, 2016 ©2016 The Authors
http://dx.doi.org/10.1016/j.molcel.2016.03.035

Enhanced Efflux Activity Facilitates Drug Tolerance in Dormant Bacterial Cells

Yingying Pu,[1,5] Zhilun Zhao,[1,5] Yingxing Li,[1,5] Jin Zou,[1] Qi Ma,[1] Yanna Zhao,[1] Yuehua Ke,[1] Yun Zhu,[1] Huiyi Chen,[2] Matthew A.B. Baker,[3] Hao Ge,[1,4] Yujie Sun,[1] Xiaoliang Sunney Xie,[1,2,*] and Fan Bai[1,*]
[1]Biodynamic Optical Imaging Center (BIOPIC), School of Life Sciences, Peking University, Beijing, China, 100871
[2]Department of Chemistry and Chemical Biology, Harvard University, Cambridge, MA 02138, USA
[3]Victor Chang Cardiac Research Institute, Sydney, NSW 2010, Australia
[4]Beijing International Center for Mathematical Research, Peking University, Beijing, China, 100871
[5]Co-first author
*Correspondence: xie@chemistry.harvard.edu (X.S.X.), fbai@pku.edu.cn (F.B.)
http://dx.doi.org/10.1016/j.molcel.2016.03.035

SUMMARY

Natural variations in gene expression provide a mechanism for multiple phenotypes to arise in an isogenic bacterial population. In particular, a subgroup termed persisters show high tolerance to antibiotics. Previously, their formation has been attributed to cell dormancy. Here we demonstrate that bacterial persisters, under β-lactam antibiotic treatment, show less cytoplasmic drug accumulation as a result of enhanced efflux activity. Consistently, a number of multi-drug efflux genes, particularly the central component TolC, show higher expression in persisters. Time-lapse imaging and mutagenesis studies further establish a positive correlation between *tolC* expression and bacterial persistence. The key role of efflux systems, among multiple biological pathways involved in persister formation, indicates that persisters implement a positive defense against antibiotics prior to a passive defense via dormancy. Finally, efflux inhibitors and antibiotics together effectively attenuate persister formation, suggesting a combination strategy to target drug tolerance.

INTRODUCTION

Multi-level stochasticity arising from mRNA transcription and protein translation leads to remarkable phenotypic heterogeneity in a population of cells with an identical genome (Elowitz et al., 2002; Lidstrom and Konopka, 2010; Raj and van Oudenaarden, 2008). Such non-genetic individuality is often proposed as a ‘bet-hedging'' mechanism (Beaumont et al., 2009; Losick and Desplan, 2008; Veening et al., 2008), whereby a clonal cell population maximizes its survival chance under rapidly changing environment by exploring diverse phenotypic solutions. One phenotype may gain a higher fitness under a given condition, and other phenotypes may be better adapted to certain other environmental situations. In the case of bacteria under drug treatment, while the majority of cells are quickly eradicated by bactericidal antibiotics, a very small group of phenotypic variants, termed persisters, show strong drug tolerance. This phenomenon was first reported in 1944 by Joseph Bigger when he studied the lethal effect of penicillin on *Staphylococcus aureus* (Bigger, 1944). Bacterial persisters have also been observed in other pathogenic species, and they may play a role in the recurrence of chronic infections. Their existence is believed to prolong and exacerbate the treatment of diseases, such as tuberculosis, cystic fibrosis associated lung infections, and candidiasis (Boucher, 2001; Chao and Rubin, 2010; Fauvart et al., 2011; LaFleur et al., 2006; Mulcahy et al., 2010).

To develop new drugs targeting chronic infections requires a deep understanding of the mechanisms underlying persister formation. However, the extremely low percentage of persister cells in a bacterial population and complex pathways involved in persister formation have delayed the study of this phenomenon. Previous studies associated persister formation mainly with cell dormancy (Balaban et al., 2004; Lewis, 2007; Shah et al., 2006), in which the important contribution from toxin-antitoxin (TA) loci has been highlighted (Balaban et al., 2004; Dörr et al., 2010; Keren et al., 2004; Vázquez-Laslop et al., 2006). TA locus encodes two components: a stable toxin that can interrupt essential cellular pathways and induce a dormancy-like state and a labile antitoxin that can conjugate the toxin to nullify such toxicity. TA modules are most likely activated by stress responses through the alarmone guanosine tetraphosphate (ppGpp) pathway (Maisonneuve et al., 2013). Furthermore, the SOS response induces persister formation through activating TisB overexpression (Dörr et al., 2010), a member of the toxin family as well. Screening a complete bacterial knockout library also identified a number of global regulators involved in persister formation (Hansen et al., 2008). For example, overexpression of *ygfA* downregulated overall transcription, and overexpression of *relE* led to a decreased protein synthesis rate (Maisonneuve et al., 2011), both of which assist bacterial drug tolerance through inducing a dormant state of the cell.

The leading theory explaining antibiotic tolerance of persister cells lies in the fact that even though antibiotic molecules bind their targets, their lethal effects have been disabled due to the extremely slow metabolic and proliferation rates in those

This is an open access article under the CC BY license (http://creativecommons.org/licenses/by/4.0/).

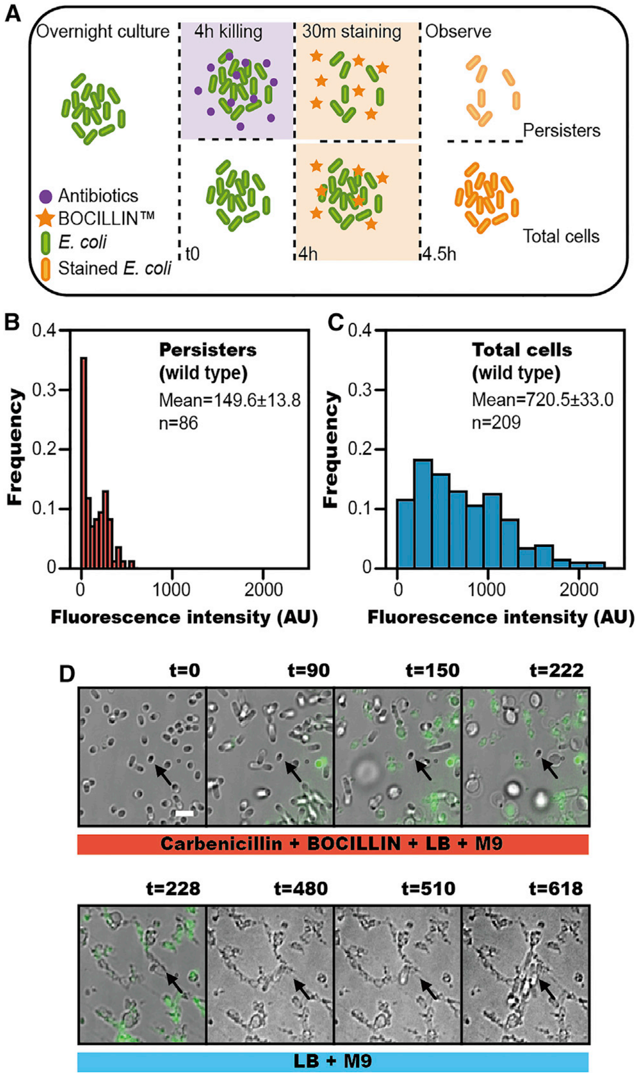

Figure 1. Lower Intracellular Antibiotic Accumulation in Persister Cells of Wild-Type *E. coli*

(A) Experimental procedure for measuring antibiotic accumulation in bacterial cells.

(B and C) Histogram of antibiotic accumulation in total cells (n = 209) and persister cells (n = 86) of wild-type *E. coli*.

(D) Time-lapse microscopy showing reduced accumulation of antibiotic in persisters (from Movie S1). The first five images are merged bright field and fluorescence images, revealing that antibiotic accumulation accompanies cell death (separate images are shown in Figure S1F). The different media added during experiment is indicated below (killing medium: 90% [v/v] M9 + 10% [v/v] LB + 150 μg/ml carbenicillin + 20 μg/ml BOCILLIN + 0.15% [w/v] methylcellulose; growth medium: 90% [v/v] M9 + 10% [v/v] LB + 5% [w/v] methylcellulose) (Maisonneuve et al., 2013). The persister cell (arrow) shows low antibiotic accumulation and regrows after removal of antibiotic (Scale bar, 3 μm; t = time in min).

See also Figure S1 and Movie S1.

dormant cells (Lewis, 2007). However, one fundamental question remains poorly addressed: do antibiotics effectively enter and accumulate in persister cells to exert their lethal effects? Antibiotic accumulation in Gram-negative bacteria is primarily influenced by two factors, membrane permeability and efflux activity. Hydrophobic antibiotics, such as aminoglycosides and macrolides, gain access into the cell through the membrane by metabolic activity-dependent diffusion, while hydrophilic antibiotics, such as β-lactam, enter the cell through porin channels to reach their targets (Delcour, 2009). Inversely, bacterial multi-drug efflux systems actively pump antibiotics out to reduce cellular drug accumulation, thus facilitating bacterial survival (Sun et al., 2014; Webber and Piddock, 2003). Therefore, stochastic and heterogeneous expression of porin or efflux proteins could lead to uneven antibiotic accumulation and consequently different drug tolerance across a bacterial population. Whether these two systems play roles in bacterial persistence thus deserves in-depth investigation.

In this study we combined in vivo fluorescent imaging and next-generation sequencing to explore the critical biological mechanisms that produce bacterial persistence. We found that enhanced efflux activity contributed strongly to persister formation and that bacterial persisters adopt a two-pronged strategy to ensure survival under antibiotic attack. Most biological processes were slowed down during dormancy, yet the efflux systems, on the contrary, displayed greater activity that further increased persister tolerance to antibiotics.

RESULTS

Antibiotic Accumulates at a Lower Level in Persister Cells

To investigate the antibiotic accumulation in bacterial persisters, we used a fluorescent β-lactam antibiotic BOCILLIN^{TM} FL Penicillin (BOCILLIN) to measure quantitatively single cell intracellular drug concentration. We isolated persisters after 150 μg/ml carbinicillin treatment (see Experimental Procedures). These drug tolerant cells had not genetically acquired resistance—after removing the antibiotic, they regrew a new bacterial population in which the majority of cells was still vulnerable to the same antibiotic (Figure S1A). Furthermore, according to previously established staining protocol (Orman and Brynildsen, 2013a), we combined fluorescence microscopy with colony counting assay to confirm that persister cells, not the viable but non-culturable cells (VBNCs), were the main cell type in these drug tolerant population (Figures S1B–S1E). After isolating persister cells, we incubated them for 30 min at 37°C (Figure 1A) with BOCILLIN. In parallel, the same staining process was carried out on a population of untreated cells that contained both persisters and susceptible cells. The fluorescent signal corresponding to antibiotic concentration in individual cells was then imaged and normalized by the cell size (the 2D projection area of the cell in bright field imaging). We followed previously described methods (Taniguchi et al., 2010) to remove background fluorescence and cell auto-fluorescence, and signal from dead cells (Figure S1B). We compared the fluorescent antibiotic intensity accumulated in bacterial persisters with that in total cells by analyzing ~150 cells from each population. Fluorescent antibiotic was found to accumulate in bacterial persisters, excluding the possibility that persisters are impermeable to antibiotics. However, the average fluorescence intensity in persisters (149.6 AU/pixel,

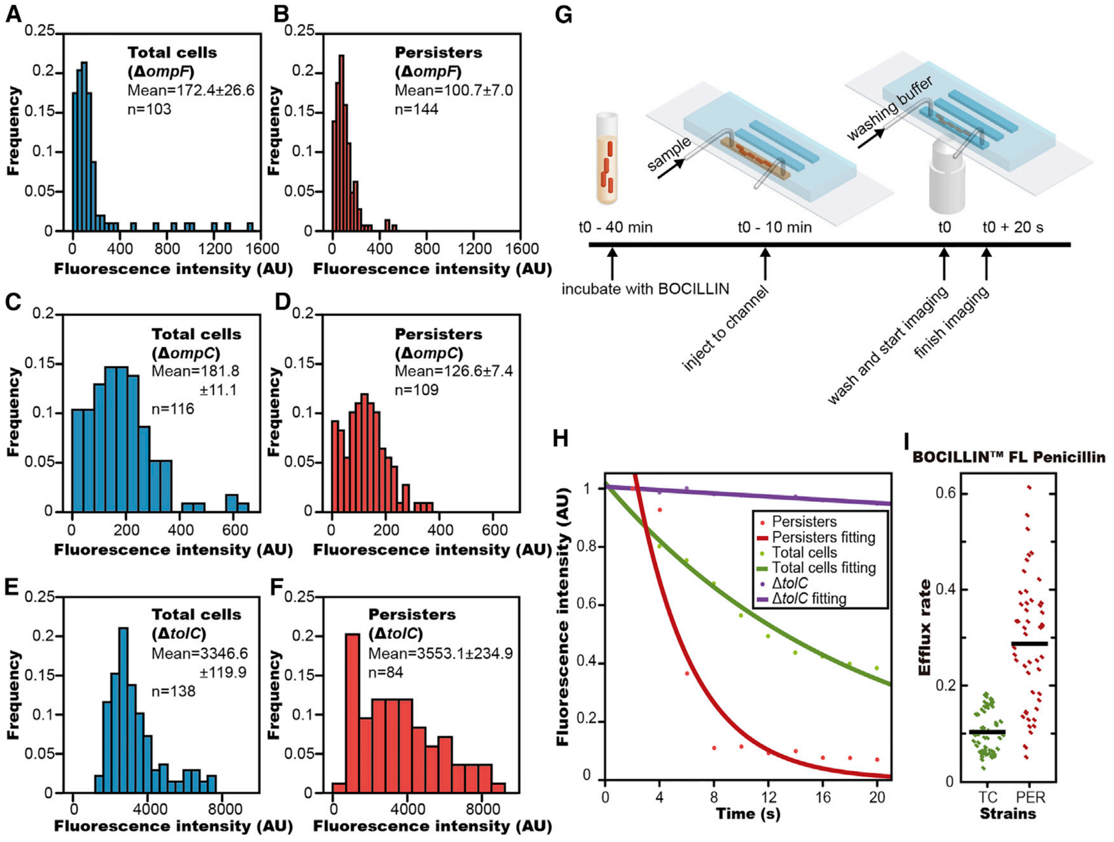

Figure 2. High Efflux Activity in Persister Cells

(A and B) Histogram of antibiotic accumulation in total cells (n = 103) and persister cells (n = 144) of *ΔompF* strain.

(C and D) Histogram of antibiotic accumulation in total cells (n = 116) and persister cells (n = 109) of *ΔompC* strain.

(E and F) Histogram of antibiotic accumulation in total cells (n = 138) and persister cells (n = 84) of *ΔtolC* strain.

(G) Experimental procedure for measuring efflux rate of fluorescent antibiotic in bacterial cells.

(H) Intracellular fluorescent intensity decay after removing antibiotic in the medium is well fit by a single exponential function. Fluorescent intensity in a persister cell (red) decayed more rapidly than in a susceptible cell (green), while a cell of *ΔtolC* mutant (purple) showed little change in fluorescence intensity with time.

(I) Statistical analysis showing that the efflux rate of antibiotic in persister cells is significantly higher than that in total cells of wild-type *E. coli* (TC, total cells; PER, persisters) ($p < 0.0001$).

See also Figure S2 and Movie S2.

Figure 1B) was only ~1/5 of that in total cells (720.5 AU/pixel, Figure 1C), indicating that the accumulated antibiotic concentration in persisters was substantially lower than that in total cells.

Additionally, we used single-cell time-lapse fluorescence microscopy to track antibiotic induced cell death and persister regrowth. As shown in Figures 1D and S1F and Movie S1, the cells with low antibiotic accumulation tolerated antibiotic attack, whereas the cells with high antibiotic accumulation gradually died and lysed. These persister cells exhibited dormancy during our time-lapse recording, featured by non-growth and non-division, but later resumed growth after the antibiotic was removed from the medium. Our observations revealed a negative correlation between the amount of antibiotic accumulated inside a cell and its probability of persisting. This proved that a reduction in the accumulation of antibiotic contributed directly to bacterial persistence.

Persister Cells Exhibit Increased Efflux Pump Activity

Antibiotic accumulation in persister cells can be lowered by decreasing the membrane permeability, increasing the efflux rate, or a combination of both. To examine exactly how antibiotic levels were lowered in persisters, we constructed two porin knockout mutants (*ΔompF* and *ΔompC*) and an efflux knockout mutant (*ΔtolC*). Using our earlier method, we compared the effect of these knockouts on each subpopulation of persisters as well as the total cell population. OmpF and OmpC are the major porin channel proteins that facilitate penetration of β-lactam antibiotics through the outer membrane (Jaffe et al., 1982; Ziervogel and Roux, 2013) and TolC is the central component of a number of bacterial efflux pumps, responsible for exporting cellular β-lactam antibiotics. First, for porin genes, we observed that the average fluorescence intensity of the total cell population in *ΔompF* mutant was 172.4 AU/pixel (Figure 2A), and in the *ΔompC* mutant the intensity was 181.8 AU/pixel (Figure 2C).

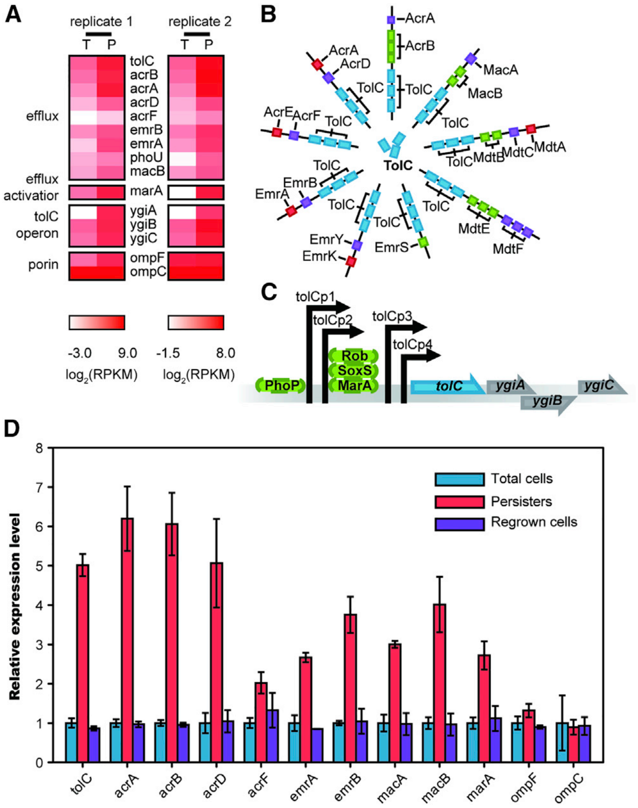

Figure 3. Efflux Gene Expression in Total and Persister Cells of Wild-Type *E. coli* Measured by RNA-seq and qPCR

(A) Heatmap showing relative transcript abundance of persistence related genes in total cells and persister cells. Scale below the heatmap indicates log2-normalized transcript abundance relative to the mean expression level (T, total cells; P, persister cells). See also Table S1.

(B) Scheme of the TolC related efflux system complexes, the number of cylinders indicating the copy number of the protein in each efflux complex.

(C) Scheme of the *tolC* operon.

(D) Relative gene expression level in total cells, persister cells, and regrown cells of wild-type *E. coli* measured by qPCR. The bars indicate mean of at least three independent experiments; error bar indicates SD.

This was a 4-fold decrease in intensity in comparison with the wild-type total cell population (720.5 AU/pixel, Figure 1C). This indicated that disabling porin led to lower antibiotic accumulation in all cells. In the subpopulation of persisters, the average fluorescence intensity of the mutants was roughly a further two-thirds lower. The *ΔompF* mutant in the persister cells was 100.7 AU/pixel (Figure 2B), and the *ΔompC* mutant in persister cells was 126.6 AU/pixel (Figure 2D). This indicated that additional factors were contributing to lowering the antibiotic accumulation in persister cells.

On the other hand, for efflux genes, the average fluorescence intensity in *ΔtolC* mutant was 3,346.6 AU/pixel (Figure 2E), a massive increase compared to the value of the wild-type (720.5 AU/pixel, Figure 1C), indicating that disabling efflux lead to extremely high antibiotic accumulation. Surprisingly, the average fluorescence intensity of persisters in *ΔtolC* mutant was 3,553.1 AU/pixel (Figure 2F), almost equal to the value in the total cells of *ΔtolC* mutant. This indicated that efflux plays a critical role in reducing cellular antibiotic concentration in persister cells of wild-type. Combined, these results suggest both decreased membrane permeability and increased efflux activity contribute to the reduction of antibiotic accumulation in wild-type persisters, but that the latter may play a more critical role.

To determine quantitatively the different efflux rates between wild-type persister and total cells, we measured the fluorescence decay of individual cells incubated in microfluidic channels (Figure S2A) by time-lapse imaging, after a sudden removal of BOCILLIN in the medium (Figure 2G; Movie S2). The fluorescence intensity in each cell decayed very quickly in the initial 20 s and then plateaued (Figure 2H). We fit the initial 20 s decay of each cell to a single exponential function $f(x) = a*exp(-b*t)$, where a represented the initial intensity and b denoted the decay rate. The decay rate had contributions from both the efflux rate and the photobleaching rate of BOCILLIN. As a negative control, we showed that the fluorescence decay in the *ΔtolC* mutant, where efflux had been deactivated, was very slow (Figure 2H). Furthermore, when treating with efflux inhibitor, the fluorescence stabilizes in the persisters (Figure S2B). These results demonstrated that photobleaching and diffusion made only a minor contribution to the intensity decay and that the fast decay observed in wild-type cells arose primarily from the efflux. Also, if we recorded for a longer time, the fluorescence of all cell types reached a plateau close to cell auto-fluorescence, excluding the possibility that BOCILLIN was covalently incorporated into the peptidoglycan of cells. We evaluated ~50 cells for each cell type, and the mean time constant (b) for decay was 0.32/s in persisters but 0.095/s in normal cells (Figure 2I), indicating that the efflux rate in persisters was approximately 3 times (p value < 0.0001) larger than that in total cells. This result strongly suggests that the lower intracellular antibiotic accumulation observed in bacterial persisters is dependent on their increased efflux activities.

Multi-Drug Efflux Genes Exhibit Significantly Higher Expression Levels in Persister Cells

Based on the above results, we hypothesized that less antibiotic accumulated in persister cells due to lower expression of porin genes and/or higher expression of efflux genes. Therefore, we performed genome-wide gene expression profiling to compare persisters and total cells using RNA-seq. Two biological replicates of persisters and total cells were prepared to assure reproducibility, and these RNA-seq results are summarized in Table S1. A large number of multi-drug efflux-associated genes showed significantly higher expression (>3-fold, p value < 0.0001) in persisters, including *tolC*, *acrA*, *acrB*, *acrD*, *acrF*, *emrA*, *emrB*, *macA*, and *macB* (Figure 3A). TolC is a common channel protein of both major and minor efflux systems, enabling interaction with many translocase complexes (Figure 3B). Deletion of the *tolC* gene alone in *E. coli* largely abolishes efflux activity (Figures 2E and 2F). Further analysis of sequencing data showed that the expression level of *marA*, one important transcriptional activator of the *tolC* operon, exhibited a far higher expression level in persisters (>10-fold, p value < 0.0001), whereas other genes *ygiA*, *ygiB*, and *ygiC*, from the *tolC* operon (Figure 3C), showed similar expression levels to *tolC*. In contrast,

the porin genes *ompF* and *ompC* showed only slightly higher transcriptional levels in persisters, indicating more porin channels on the membrane of those cells. Together these results demonstrated that higher efflux rate, rather than lower membrane permeability, was responsible for reduced antibiotic accumulation in persister cells. qRT-PCR (Figure 3D) agreed with sequencing results and confirmed that these efflux genes were upregulated in persister cells, whereas β-lactam influx porin genes *ompF* and *ompC* had a similar expression level or were only slightly upregulated in persister cells. Both transcriptome sequencing and qRT-PCR results revealed that *tolC* expression in persisters was at least 8-fold greater than that in total cells, making it a potential biomarker to differentiate persisters from normal cells. Whole-genome sequencing (Table S2) and qRT-PCR (Figure 3D) of persister and regrown populations confirmed that enhanced expression of efflux genes is transient and reversible, featuring a non-genetic factor that assists bacterial persistence.

High Expression Level of *tolC* Directly Assists Bacterial Persistence

We next used single-cell fluorescence imaging to measure the dependence of bacterial persistence on expression level of the central efflux component TolC. First, we monitored the natural fluctuation in concentration of TolC among the cell population. Since trimers of TolC form barrel-like channels in functioning efflux pumps on the outer membrane (Du et al., 2014), conventional labeling of TolC by fluorescent proteins has been difficult. To solve this, we inserted a six amino acid tetracysteine tag into the linker structure of chromosomally encoded *tolC* (TC tag-TolC strain, 76RP). This had little impact on either TolC expression level or function (Figures S3A–S3C). In this way, the tagged TolC protein was fluorescently labeled by tetracysteine-based protein detection (FlAsH labeling) (Griffin et al., 1998). As shown in Figure 4A (upper panel), the fluorescence intensity of TolC-FlAsH across a population of total cells was distributed in a broad gamma distribution with a long tail at high fluorescence, indicating a noticeable heterogeneous expression of *tolC* among different cells.

We monitored the antibiotic killing process under a microscope using time-lapse imaging at controlled temperature. After 4 hr treatment with high concentration (150 μg/ml) carbenicillin, the majority of the cells had died and lysed, leaving only a few surviving persisters. By tracking the spatial coordinates of these persister cells, we found their original positions in the cell population and calculated their TolC-FlAsH fluorescent intensity prior to the antibiotic attack. We found persisters emerged mostly from the long high-intensity tail of the TolC-FlAsH fluorescence distribution (81.0% of persister cells arose from 8.5% of total cell population with highest *tolC* expression) (Figure 4A, lower panel).

Typical examples capturing the dynamic process of persister formation are shown in Figures 4B and S3D and Movie S3. In a single field of view, many cells were susceptible to carbenicillin and lysed gradually. They were featured by low TolC-FlAsH intensity. In contrast, persisters occurred rarely and showed high intensity in TolC-FlAsH fluorescence. We confirmed again that the persister cells neither grew nor divided during our time-lapse recording, consistent with cell dormancy. We then tested whether these cells could resurrect when antibiotics were removed from the growth medium. As shown in Figure 4B and Movie S3, a short delay after antibiotic removal, the persisters, indicated by high fluorescence intensity of TolC-FlAsH, began to elongate, divide, and produce new microcolonies.

Having observed that all persisters exhibit higher *tolC* expression, we sought to examine whether all cells displaying high *tolC* expression were more likely to form persisters. We utilized FACS to group cells according to their TolC level, quantified by FlAsH staining in the 76RP strain (Figure 4C). Both staining and cell sorting did not affect persister formation frequency (Figure S3E). The antibiotic sensitivity assay was subsequently performed on each group of cells to evaluate the frequency of persister formation. As presented in Figures 4D and 4E, the highly fluorescent subpopulation (1%, group C) expressing TC tag-TolC showed 18-fold enrichment in the number of persister cells in comparison with that of the total population (100%, group A), and 24-fold enrichment in comparison with the remaining dimmer population (97.8%, group B). Altogether, this positive correlation between *tolC* expression level and probability of persister formation suggested that high expression level of *tolC* is critical to promote persister formation.

Bacterial Persistence Negatively Correlates with Intracellular Antibiotic Accumulation and Positively Correlates to Efflux Activity

We quantified the correlation between efflux gene expression; intracellular antibiotic accumulation; and persister formation frequency using wild-type *E. coli*, *ΔtolC*, *tolC* overexpression, and *tolC* rescue strains. First, we measured the *tolC* gene expression by qRT-PCR. In the overexpression strain, *tolC* concentration was about 3-fold of that in the wild-type strain; no *tolC* expression was detected in the *ΔtolC* strain; and in the rescue strain, *tolC* expression returned to a similar level as that in the wild-type strain (Figure 5A). We measured the antibiotic accumulation in these strains with BOCILLIN using the above method. Fluorescent antibiotic accumulation was negatively correlated with *tolC* expression, with significantly higher intensities (~2,600 AU/pixel) in the *ΔtolC* strain and lower intensities (~10 AU/pixel) in the overexpression strain. In the rescued strain, antibiotic accumulation was at a similar level as that in the wild-type strain (Figure 5B). We then used an antibiotic sensitivity assay to measure the frequency of persister formation. The initial concentration of these strains were all set to be $\sim 5 \times 10^7$ cells/ml before antibiotic treatment. While wild-type *E. coli* produced $\sim 3 \times 10^4$ cells/ml antibiotic-tolerant persisters, the *ΔtolC* mutant produced only ~50 cells/ml (Figures 5C and 5D). The overexpression strain produced $\sim 1.6 \times 10^5$ cells/ml persister cells, roughly 5-fold the number produced by the wild-type strain (Figures 5C and 5D). The rescued strain recovered the low-persister frequency in the *ΔtolC* strain (Figures 5C and 5D). Thus, the *tolC* expression level was negatively correlated to the intracellular antibiotic accumulation, and positively correlated to persister formation.

Efflux Pump Inhibitors Combined with Antibiotics to Eradicate Persisters

Since persister formation frequency was dramatically reduced in efflux knockout strains (Figures S4A and S4B), we tested a

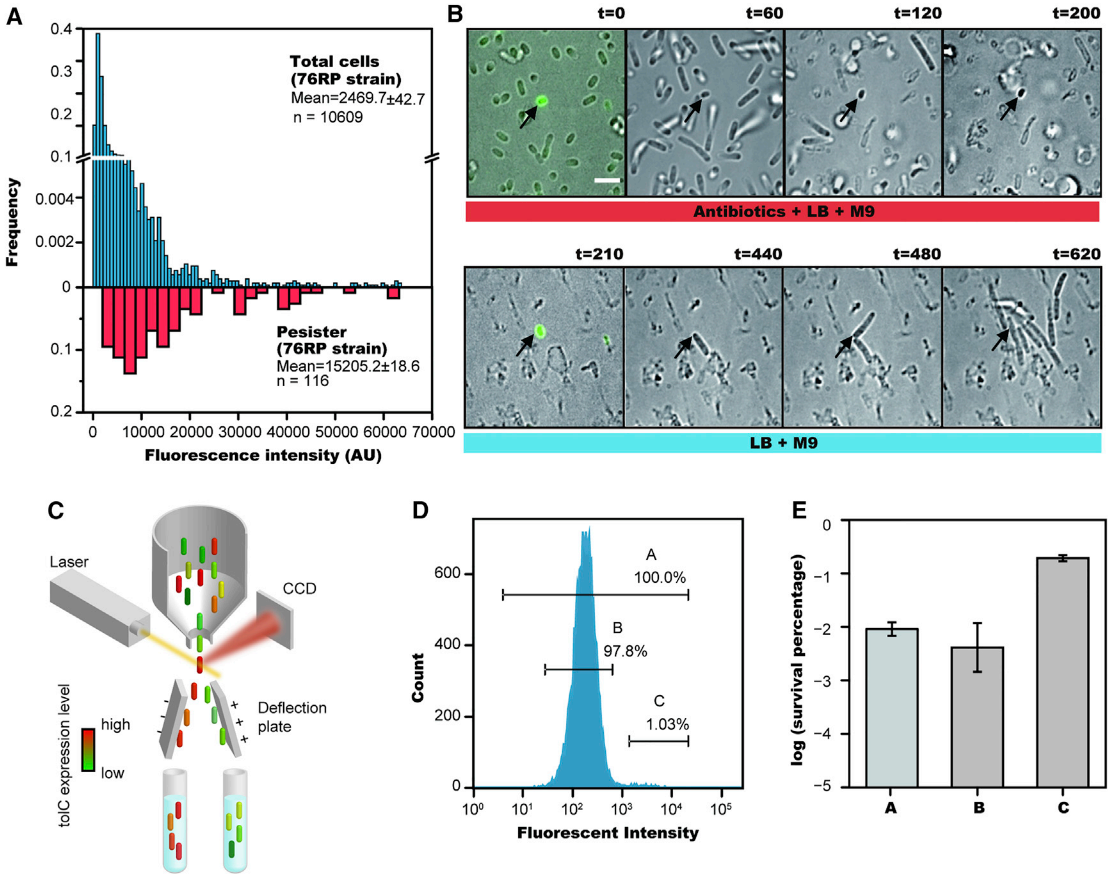

Figure 4. The Dependence between Persister Formation Frequency and TolC Expression Level

(A) TolC expression level in total cells of TC tag-TolC strain measured by Tetracysteine-based protein detection (FlAsH imaging, upper panel); TolC expression level in persister cells that survived antibiotic treatment (lower panel).

(B) Time-lapse microscopy showing that persister cells express a high level of TolC (from Movie S3). The first and fifth images are the merged bright field and fluorescent images (separate bright field and fluorescent images are shown in Figure S3D). The different media added during the experiment are indicated below (killing medium: 90% [v/v] M9 + 10% [v/v] LB + 150 μg/ml carbenicillin + 0.15% [w/v] methylcellulose; growth medium: 90% [v/v] M9 + 10% [v/v] LB + 5% [w/v] methylcellulose). The persister cell (arrow), shows a significantly higher expression of TolC, and regrows after removal of antibiotic (from Movie S3). (Scale bar, 3 μm; t = time in min).

(C) Experimental procedure for sorting cells by expression level of persistence related genes.

(D) Distribution of fluorescence intensity indicating TolC expression levels of 76RP strain. Stationary-phase cells were sorted into three groups: group A containing the total cells (100.0%), group B including the majority of total cells except for those with highest fluorescence intensity (97.8%), and group C containing a sub-population with highest fluorescence intensity (1.0%).

(E) Bar plot representing cell survival rate (percentage log scale) after 4 hr carbenicillin treatment of three groups sorted by flow cytometer, revealing that the survival rate significantly increases in group C. The bars indicate mean of at least three independent experiments; error bar indicates SD.

See also Figure S3 and Movie S3.

therapeutic strategy that combined antibiotics with efflux pump inhibitors (EPIs). Phenylalanine arginyl β-naphthylamide (PAβN) and 1-(1-Naphthylmethyl) piperazine (NMP) are well studied inhibitors that can effectively block TolC-composed efflux pumps through competitive substrate export. When treating wild-type *E. coli* with carbenicillin, the addition of PAβN had little effect, but the addition of NMP greatly improved lethality. The persister number was reduced to less than 20% of the number surviving when carbenicillin was applied alone (Figure 5E). We further tested if such potentiating effect was observable with other antibiotics. Starting again from an initial concentration of 5×10^7 cells/ml, the persister fraction in cells treated with cloxacillin (β-lactam) was $\sim 5 \times 10^4$ cells/ml. The addition of PAβN and NMP reduced this number to $\sim 8 \times 10^3$ cells/ml and $\sim 3 \times 10^3$ cells/ml (Figure 5F), about 17% and 12% of the number that survived in the absence of inhibitors, respectively. The same effect was also observed in bacteria treated with nalidixic acid (quinolone), which produced $\sim 2 \times 10^6$ cells/ml persisters. The addition of PAβN and NMP reduced this number to $\sim 2.5 \times 10^4$ cells/ml and $\sim 5 \times 10^4$ cells/ml (Figure 5G), about 0.5% and 3% of the number surviving without inhibitors, respectively. Those EPIs alone did not influence cell growth (Figure S4C). These results show that the combining antibiotics with EPIs offers therapeutic promise in the fight against bacterial persisters.

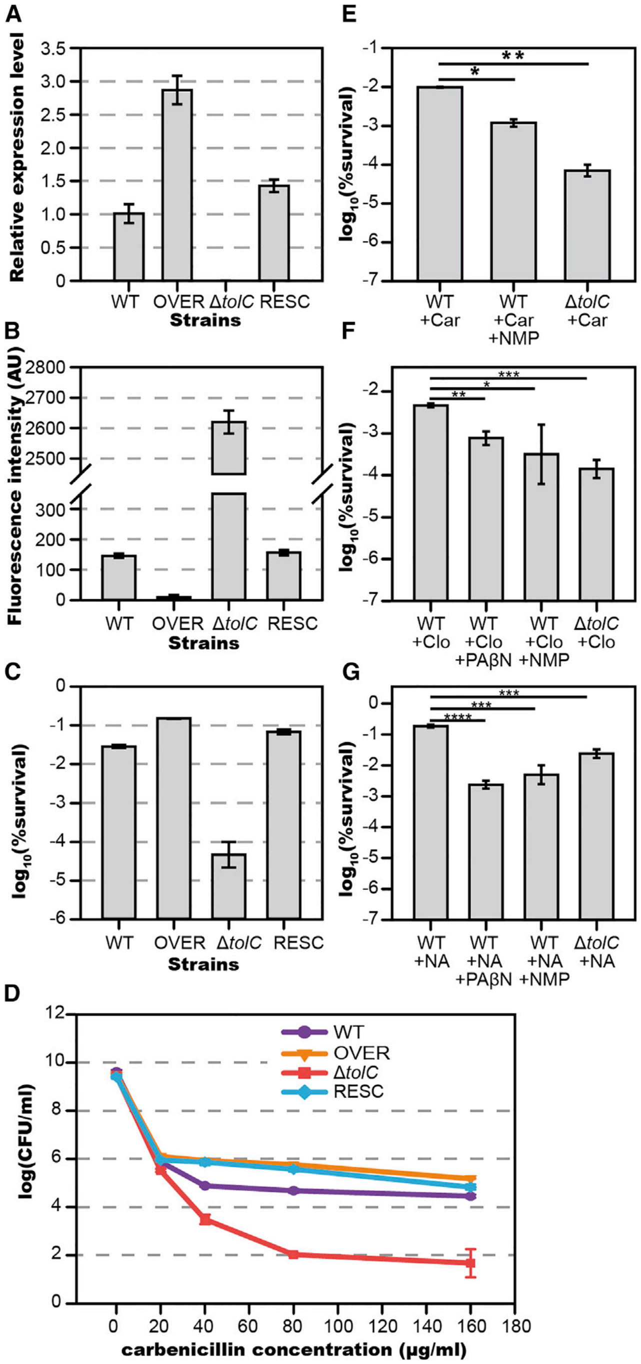

Figure 5. Persister Formation Frequency Positively Correlates with Efflux Gene Expression Level and Negatively Correlates with Intracellular Antibiotic Accumulation. Antibiotics Lethal Effects Were Enhanced by Addition of Efflux Inhibitors

(A) Relative *tolC* expression level of the four strains measured by qPCR. WT (wild-type strain BW25113), OVER (*tolC* overexpression strain, by pBAD::*tolC* in BW25113, induced by $10^{-5\%}$ arabinose), *ΔtolC* (*tolC* knockout strain, Key::JW5503), and RESC (*tolC* rescued strain, by pBAD::*tolC* in *ΔtolC* strain, induced by $10^{-6\%}$ arabinose).

(B) Relative antibiotic accumulation in the four strains determined by fluorescence microscopy.

(C) Persister formation frequency of the four strains determined by antibiotic susceptibility measurement. The correlation coefficiency between intracellular antibiotic accumulation level and *tolC* expression level is −0.776; between intracellular antibiotic accumulation level and probability of persistence is −0.989; between *tolC* expression level and probability of persistence is 0.848.

(D–G) (D) Persister formation frequency of the four strains under antibiotic treatment of different concentrations. Antibiotics lethal effects were enhanced by efflux inhibitors; carbenicillin (E), cloxacillin (F), and nalidixic acid (G). Abbreviations: Car, carbenicillin. NA, nalidixic acid. Clo, cloxacillin. PAβN, Phenylalanine arginyl β-naphthylamide. NMP, 1-(1-Naphthylmethyl) piperazine. The bars indicate mean of at least three independent experiments; error bar indicates SD (*p value < 0.1; **p value < 0.01; ***p value < 0.001; ****p value < 0.0001).

See also Figure S4.

Active Efflux Plays a Primary Role in the Drug Tolerance of Persister Cells

Further analysis of our transcriptome sequencing data identified several other groups of genes that showed differential expression between persister and total cells (Figure 6A; Table S1), implying multiple pathways that can be involved in persister formation. Global regulators that mediated downstream pathways leading to cell dormancy, including *dnaJ*, *dnaK*, *hupA*, *hupB*, *ygfA*, and *yigB* (Hansen et al., 2008), presented higher expression level in persister cells than in total cells. Toxin genes and their regulators from the TA modules *hipA*, *crp*, *dksA*, *spoT*, *lon*, and *relA* displayed upregulation in persister cells, whereas antitoxin gene *hipB* showed no changes between the two cell types (Balaban et al., 2004; Amato et al., 2013; Hansen et al., 2008; Maisonneuve et al., 2011, 2013). Oxidative stress response related genes, including *soxS* (Wu et al., 2012), *pspA* (Vega et al., 2012), and *ansA*, were also upregulated in persisters, as well SOS response genes *dinG*, *uvrD*, *ruvA*, and *ruvB* (Theodore et al., 2013). Finally, slow metabolism-related genes *plsB* (Spoering et al., 2006) and *phoU* (Li and Zhang 2007) were also highly expressed in persisters. The higher expression of these genes promotes a cell's entry into the dormant state. However, unexpectedly, efflux pumps were actively working in those dormant cells, conferring upon them an increased capacity to tolerate antibiotic attack.

In order to rank the individual genes contributing to persistence, we constructed two libraries: a fluorescent protein EGFP fusion library and an ordinary knockout library covering reported genes associated with persistence. For the EGFP fusion library, we again used FACS to isolate cells with high fluorescence intensity (1%) and then performed the antibiotic sensitivity assay on these cells and compared that with the assay on the total population (100%). The results revealed that cells with higher expression levels of *tolC* showed the largest increase in bacterial persister enrichment (18.6-fold, p value = 0.00008) (Figure 6B). On the other hand, higher expression of other persistence related genes had less impact on persister enrichment (Figure 6B), of which *pspA* was the only gene that caused a significant increase in persister formation (3.2-fold, p value = 0.007). For the knockout library, we performed the antibiotic sensitivity assay to determine the effect of each knockout on bacterial survival rate. The strain carrying a deletion of *tolC* suffered the greatest reduction (29-fold) in persister formation following treatment with carbenicillin. Deletion of other individual persistence-related

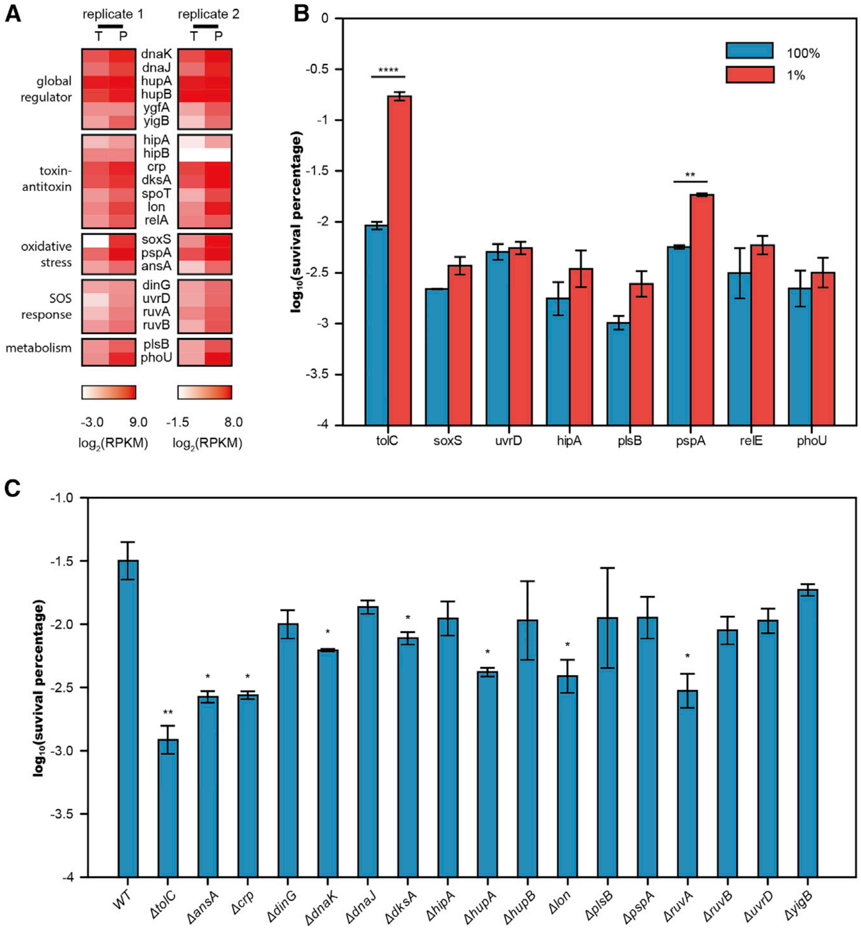

Figure 6. System-wide Comparison of the Contribution of Persistence Genes to Bacterial Drug Tolerance

(A) Heatmap showing relative transcript abundance of persistence related genes in total cells and persister cells. Scale below the heatmap indicates log2-normalized transcript abundance relative to the mean expression level (T, total cells; P, persister cells). See also Table S1.

(B) Bar plot representing cell survival rate (percentage log scale) after 4 hr carbenicillin treatment of two groups sorted by flow cytometer from fluorescently labeled *tolC*, *soxS*, *uvrD*, *hipA*, *plsB*, *pspA*, *relE*, and *phoU* strains, respectively.

(C) Bar plot representing cell survival rate (in logarithm scale) after 4 hr carbenicillin treatment of knockout strains *ΔtolC*, *ΔansA*, *Δcrp*, *ΔdinG*, *ΔdnaK*, *ΔdnaJ*, *ΔdksA*, *ΔhipA*, *ΔhupA*, *ΔhupB*, *Δlon*, *ΔplsB*, *ΔpspA*, *ΔruvA*, *ΔruvB*, *ΔuvrD*, and *ΔyigB*. The bars indicate mean of at least three independent experiments; error bar indicates SD (*p value < 0.1; **p value < 0.01; ***p value < 0.001; ****p value < 0.0001).

genes caused a lower reduction in persister formation (Figure 6C), including *ΔansA* (14-fold), *Δcrp* (14-fold), *ΔdnaK* (6-fold), *ΔdksA* (5-fold), *ΔhipA* (3-fold), *ΔhupA* (9-fold), *Δlon* (9-fold), and *ΔruvA* (11-fold). According to previous research (Maisonneuve et al., 2011), single deletion of *lon* led to a significant reduction in persister formation (similar to our results here), and the effect was comparable to that caused by deletion of *10 TA* genes together. Of note, our results show that deletion of *tolC* results in a 29-fold reduction in persister formation, a three-times further decrease when compared to that caused by deletion of *lon*.

The above results suggest that multiple mechanisms can lead to persistence, and while the contribution of each mechanism varies, the efflux system holds the most significant role in persister formation, at least in our experimental conditions. Our observations highlight the precise control behind persister formation. Bacterial persisters manage to combine two seemingly contradictory mechanisms to survive antibiotic attack: they slow down most physiological processes during dormancy and at the same time activate their efflux systems to remove intracellular antibiotics.

DISCUSSION

Gene expression is intrinsically stochastic. The variation in synthesis and breakdown of molecules in biochemical reactions that initiate and regulate transcription and translation gives rise to the heterogeneity in amount of protein expressed in a population of cells (Huang, 2009; Elowitz et al., 2002; Raj and van Oudenaarden, 2008). This was recently demonstrated by a high-throughput system-wide analysis (Taniguchi et al., 2010). In our experiments, we have observed that populations of bacteria have a heterogeneous distribution of efflux pumps due to stochastic gene expression. The subpopulation with the higher expression of efflux genes is able to pump more antibiotics outside the cell to reduce intracellular antibiotic accumulation and is thus more likely to persist. Our observation is consistent with a previous study (Allison et al., 2011a) in which the authors introduced metabolites to induce antibiotic uptake, eradicating bacterial persisters and implying that a lower antibiotic concentration inside the cell is key to being persistent.

Pre-existing versus Induced Expression of *tolC* in Persister Cells

Whether this overexpression of efflux genes preempts drug treatment or is induced by antibiotic attack as a protective response in persisters remains inconclusive. Figure 4A shows that surviving persister cells belong to cells with higher fluorescent intensity of TolC-FlAsH, indicating such upregulation of efflux genes in persister cells may preexist. However, we cannot rule out the possibility that some of this higher expression was induced by drug treatment. Recent reports have found that indole signaling contributes to persister formation, possibly via upregulation of the efflux pumps through activation of the two-component systems BaeS-BaeR or CpxA-CpxR and the PspAB and OxyR pathways as a result of stress response (Vega et al., 2012; Hirakawa et al., 2005). Additionally, at a population level,

indole molecules produced by highly resistant cells have been shown to provide protection to other more susceptible cells by activating their drug efflux pumps and oxidative-stress protective mechanisms (Lee et al., 2010). This facilitated the survival of the whole population. We believe this population effect may play a role in persister formation; however, the exact ratio between pre-existing and drug induced persisters deserves further investigation.

Lack of Cooperation between Porin Genes and Efflux Genes

There are two ways to reduce intracellular antibiotic concentration: to lessen the membrane permeability by expressing fewer porin channels and to enhance efflux pump expression and activity. In our study, we proved that the expression of efflux genes was upregulated in persister cells. However, unexpectedly, we found that the porin gene *ompF*, which is critical for β-lactam antibiotic transportation, was also slightly upregulated in persister cells. Such increased expression of porin genes would amplify antibiotic entry and therefore lead to cell death, attenuating the chance of persisting. We do not understand the mechanism underlying this upregulation of *ompF* in persister cells, yet this further highlights the significant role of efflux pumps in bacterial persistence. It is possible that TolC protein might exert its effect on *ompF* promoter area or the terminal end of *ompF* gene to regulate gene expression (Misra and Reeves, 1987). Therefore, the higher expression of *ompF* in persiser cells might be a side-effect caused by upregulation of *tolC*, as previous work has shown that downregulation of *tolC* led to a reduction in *ompF* expression (Morona and Reeves, 1982).

Use of Efflux Inhibitors in Eradicating Bacterial Persisters

To effectively treat chronic infectious diseases, specific anti-persistence therapies are required to eradicate persisters or to prevent their formation. Several genes that contribute to persister formation are current targets for drug design, including *hipBA*, *tisAB*, *phoU*, *edpA*, and *plsB etc*. (Fauvart et al., 2011).

EPIs can be combined with conventional antibiotics to reduce efflux pump-associated intrinsic resistance or acquired multidrug resistance (Zechini and Versace, 2009). For example, broad-spectrum EPI PAβN enhanced the lethality of levofloxacin against drug-resistant *Pseudomonas aeruginosa* (Lomovskaya et al., 2001), and another pump inhibitor-verapamil reduced macrophage-induced drug tolerance in *Mycobacterium marinum* (Adams et al., 2011). In our current study, we have demonstrated that EPIs work synergistically with different antibiotics to reduce persister formation. This further emphasized the need to match specific EPIs with antibiotics to treat persistent bacterial infections.

Latent but Active: Enhanced Efflux Activity Facilitates Drug Tolerance in Dormant Bacterial Cells

Previously, cell dormancy was considered as the leading mechanism resulting in bacterial persistence (Balaban et al., 2004; Rotem et al., 2010). In these cells, antibiotics failed to eradicate bacterial cells due to dormant downstream pathways, though antibiotics still bound to their molecular targets. It has long been proposed that multiple mechanisms contribute to bacterial persistence (Lewis, 2010; Allison et al., 2011b), and previous studies have identified many dormant pathways and genes related to persistence. These include global regulators (Hansen et al., 2008) that lead to slower cell growth, toxin genes in TA modules (Balaban et al., 2004; Maisonneuve et al., 2011) that induce a dormant state, oxidative stress response pathways (Vega et al., 2012; Wu et al., 2012), SOS response pathways (Theodore et al., 2013), and metabolism pathways (Spoering et al., 2006; Li and Zhang 2007). However, dormancy alone cannot explain persistence (Orman and Brynildsen, 2013b). We used transcriptome sequencing to identify upregulation of an additional group of efflux genes that contribute to persister formation and further established that cells with upregulated efflux genes showed elevated persistence while efflux knockout mutants showed attenuated persistence. Together, this suggests efflux pumps play a critical role in bacterial drug tolerance.

As demonstrated by our results, in addition to a “passive defense” via dormancy, bacterial persisters employ an “active defense” to pump antibiotics out and reduce intracellular drug concentration through enhanced efflux activity. Our findings indicate that while most biological processes in persisters are slowed down, the efflux systems, on the contrary, become more active and effectively promote antibiotic persistence. It is surprising to see that active efflux and dormancy coexist in many persister cells since dormancy seems to exclude active efflux. How bacterial cells control precisely which pathways to shutdown and which to activate, and whether dormancy and active efflux are intrinsically co-regulated, remains unknown. Our observation that persisters combine two contradictory mechanisms to survive antibiotic attack highlights the advanced network regulation endowed by natural selection, and also the challenges we face in eradicating these drug tolerant cells.

EXPERIMENTAL PROCEDURES

Bacterial Strains and Plasmid Construction

Wild-type strain BW25113 and its *tolC* knockout strain (JW5503) were gifts from Yale Genetic Stock Center. The detailed information for the construction of *tolC* overexpression strain (Figure S5A), *tolC* rescued strain, 76RP strain (Figure S5B), and other strains are provided in Supplemental Experimental Procedures. Primers used are listed in Table S3.

Antibiotic Killing and Persister Isolation

Frozen stocks of *E. coli* strains were diluted by 1:1,000 into LB medium and cultured overnight. The overnight culture was diluted in fresh LB to appropriate concentration (OD_{600} = 0.2), which was then split into two flasks. To one flask carbenicillin (150 μg/ml, Sigma) was added at a desired concentration to yield persisters. To the other flask the same volume of sterile Milli-Q (which was the solvent of carbenicillin solution) was added to yield total cells. The cultures were returned to the 37°C shaker for 4 hr. Then the cells were collected by centrifuging at 4000 × *g* for 5 min and washed with M9 minimal medium with 150 μg/ml carbenicillin three times to isolate unlysed persister cells and total cells. To confirm that the persisters we analyzed were from the second killing phase, the biphasic killing curves of each strain were shown in Figure S6.

Fluorescent Microscopy

All the imaging work was performed on an inverted microscope (Nikon Eclipse *Ti*). The illumination was provided by different solid state lasers (Coherent), at 488nm for BOCILLIN and FlAsH, and 532nm for propidium iodide, respectively. The fluorescent signal of cells was collected by an EMCCD camera (Photometrics Evolve 512). The appropriate filter sets were selected for each

fluorophore according to its spectrum. Image analysis was done by ImageJ software (Fiji). Detailed information is described in Supplemental Experimental Procedures.

Flow Cytometry Analysis and Cell Sorting

All samples were measured on a BD FACSAria III flow cytometer. FlAsH labeling 76RP strain or chromosomal *geneX-egfp* translational fusion strain in the stationary phase was resuspended in sterile 1XPBS, which was also used as sheath fluid in flow cytometer. Microorganisms were identified by FSC (forward scatter) and SSC (side scatter) parameters. Cells were sorted into three groups based on their fluorescence intensity (488-nm excitation with 530/30-nm-band-pass filter) using a 70 μm nozzle. Approximately 100,000 cells were collected in each group. The results were analyzed by FlowJo V10 software (Treestar, Inc.).

Antibiotic Sensitivity Assay

The overnight cultures of *E. coli* strains were diluted by 1:20 into fresh LB with antibiotic carbenicillin (final concentration of 0 μg/ml, 20 μg/ml, 40 μg/ml, 80 μg/ml, and 160 μg/ml, respectively). Then the culture was returned to the 37°C shaker for another 4 hr. Samples were withdrawn and appropriately diluted in LB medium and spotted on an LB agar plate for overnight culture at 37°C. Colony counting was performed the next day. For the inhibitor assay, PAβN (sigma) or NMP (sigma) at a final concentration of 100 μM was added into medium at the same time points with antibiotics.

ACCESSION NUMBERS

Sequencing data have been deposited at the NCBI sequence read archive under accession number SRA: SRP072433 (http://www.ncbi.nlm.nih.gov/sra/SRP072433).

SUPPLEMENTAL INFORMATION

Supplemental Information includes six figures, three tables, three movies, and Supplemental Experimental Procedures and can be found with this article online at http://dx.doi.org/10.1016/j.molcel.2016.03.035.

AUTHOR CONTRIBUTION

Y.P., Z.Z., and Y.L. designed the study; carried out experiments; and analyzed the data; J.Z., Y. Zhao, and Y. Zhu performed mutagenesis experiments; Q.M., H.G., Y.S., Y.K., and M.A.B.B. analyzed the sequencing data and performed related bioinformatics analyses; F.B. and X.S.X. designed and supervised the study; Y.P., Z.Z., Y.L., and F.B. wrote the manuscript with contributions from H.C. and M.A.B.B.

ACKNOWLEDGMENTS

We thank Y. Huang and L. Zhao from BIOPIC of Peking University for help in microfluidic fabrication; Y. Zhang, M. Yang, and P. Zhu from BIOPIC for processing sequencing data; A. Streets from BIOPIC for valuable discussions. We also thank National Center for Protein Sciences Beijing (Peking University) and Z. Fu for assistance with FACS. This work was financially supported by Bill & Melinda Gates Foundation to X.S. Xie, Peking University 985 funding, the National Natural Science Foundation of China (No. 31370847, No. 31327901), and the Recruitment Program of Global Youth Experts to F. Bai.

Received: September 27, 2015
Revised: December 28, 2015
Accepted: March 30, 2016
Published: April 21, 2016

REFERENCES

Adams, K.N., Takaki, K., Connolly, L.E., Wiedenhoft, H., Winglee, K., Humbert, O., Edelstein, P.H., Cosma, C.L., and Ramakrishnan, L. (2011). Drug tolerance in replicating mycobacteria mediated by a macrophage-induced efflux mechanism. Cell *145*, 39–53.

Allison, K.R., Brynildsen, M.P., and Collins, J.J. (2011a). Metabolite-enabled eradication of bacterial persisters by aminoglycosides. Nature *473*, 216–220.

Allison, K.R., Brynildsen, M.P., and Collins, J.J. (2011b). Heterogeneous bacterial persisters and engineering approaches to eliminate them. Curr. Opin. Microbiol. *14*, 593–598.

Amato, S.M., Orman, M.A., and Brynildsen, M.P. (2013). Metabolic control of persister formation in Escherichia coli. Mol. Cell *50*, 475–487.

Balaban, N.Q., Merrin, J., Chait, R., Kowalik, L., and Leibler, S. (2004). Bacterial persistence as a phenotypic switch. Science *305*, 1622–1625.

Beaumont, H.J., Gallie, J., Kost, C., Ferguson, G.C., and Rainey, P.B. (2009). Experimental evolution of bet hedging. Nature *462*, 90–93.

Bigger, J.W. (1944). Treatment of staphylococcal infections with penicillin - By intermittent sterilisation. Lancet *2*, 497–500.

Boucher, R.C. (2001). Pathogenesis of cystic fibrosis airways disease. Trans. Am. Clin. Climatol. Assoc. *112*, 99–107.

Chao, M.C., and Rubin, E.J. (2010). Letting sleeping dos lie: does dormancy play a role in tuberculosis? Annu. Rev. Microbiol. *64*, 293–311.

Delcour, A.H. (2009). Outer membrane permeability and antibiotic resistance. Biochim. Biophys. Acta *1794*, 808–816.

Dörr, T., Vulić, M., and Lewis, K. (2010). Ciprofloxacin causes persister formation by inducing the TisB toxin in Escherichia coli. PLoS Biol. *8*, e1000317.

Du, D., Wang, Z., James, N.R., Voss, J.E., Klimont, E., Ohene-Agyei, T., Venter, H., Chiu, W., and Luisi, B.F. (2014). Structure of the AcrAB-TolC multidrug efflux pump. Nature *509*, 512–515.

Elowitz, M.B., Levine, A.J., Siggia, E.D., and Swain, P.S. (2002). Stochastic gene expression in a single cell. Science *297*, 1183–1186.

Fauvart, M., De Groote, V.N., and Michiels, J. (2011). Role of persister cells in chronic infections: clinical relevance and perspectives on anti-persister therapies. J. Med. Microbiol. *60*, 699–709.

Griffin, B.A., Adams, S.R., and Tsien, R.Y. (1998). Specific covalent labeling of recombinant protein molecules inside live cells. Science *281*, 269–272.

Hansen, S., Lewis, K., and Vulić, M. (2008). Role of global regulators and nucleotide metabolism in antibiotic tolerance in Escherichia coli. Antimicrob. Agents Chemother. *52*, 2718–2726.

Hirakawa, H., Inazumi, Y., Masaki, T., Hirata, T., and Yamaguchi, A. (2005). Indole induces the expression of multidrug exporter genes in Escherichia coli. Mol. Microbiol. *55*, 1113–1126.

Huang, S. (2009). Non-genetic heterogeneity of cells in development: more than just noise. Development *136*, 3853–3862.

Jaffe, A., Chabbert, Y.A., and Semonin, O. (1982). Role of porin proteins OmpF and OmpC in the permeation of beta-lactams. Antimicrob. Agents Chemother. *22*, 942–948.

Keren, I., Shah, D., Spoering, A., Kaldalu, N., and Lewis, K. (2004). Specialized persister cells and the mechanism of multidrug tolerance in Escherichia coli. J. Bacteriol. *186*, 8172–8180.

LaFleur, M.D., Kumamoto, C.A., and Lewis, K. (2006). Candida albicans biofilms produce antifungal-tolerant persister cells. Antimicrob. Agents Chemother. *50*, 3839–3846.

Lee, H.H., Molla, M.N., Cantor, C.R., and Collins, J.J. (2010). Bacterial charity work leads to population-wide resistance. Nature *467*, 82–85.

Lewis, K. (2007). Persister cells, dormancy and infectious disease. Nat. Rev. Microbiol. *5*, 48–56.

Lewis, K. (2010). Persister cells. Annu. Rev. Microbiol. *64*, 357–372.

Li, Y., and Zhang, Y. (2007). PhoU is a persistence switch involved in persister formation and tolerance to multiple antibiotics and stresses in Escherichia coli. Antimicrob. Agents Chemother. *51*, 2092–2099.

Lidstrom, M.E., and Konopka, M.C. (2010). The role of physiological heterogeneity in microbial population behavior. Nat. Chem. Biol. *6*, 705–712.

CellPress

Lomovskaya, O., Warren, M.S., Lee, A., Galazzo, J., Fronko, R., Lee, M., Blais, J., Cho, D., Chamberland, S., Renau, T., et al. (2001). Identification and characterization of inhibitors of multidrug resistance efflux pumps in Pseudomonas aeruginosa: novel agents for combination therapy. Antimicrob. Agents Chemother. *45*, 105–116.

Losick, R., and Desplan, C. (2008). Stochasticity and cell fate. Science *320*, 65–68.

Maisonneuve, E., Shakespeare, L.J., Jørgensen, M.G., and Gerdes, K. (2011). Bacterial persistence by RNA endonucleases. Proc. Natl. Acad. Sci. USA *108*, 13206–13211.

Maisonneuve, E., Castro-Camargo, M., and Gerdes, K. (2013). (p)ppGpp controls bacterial persistence by stochastic induction of toxin-antitoxin activity. Cell *154*, 1140–1150.

Misra, R., and Reeves, P.R. (1987). Role of micF in the tolC-mediated regulation of OmpF, a major outer membrane protein of Escherichia coli K-12. J. Bacteriol. *169*, 4722–4730.

Morona, R., and Reeves, P. (1982). The tolC locus of Escherichia coli affects the expression of three major outer membrane proteins. J. Bacteriol. *150*, 1016–1023.

Mulcahy, L.R., Burns, J.L., Lory, S., and Lewis, K. (2010). Emergence of Pseudomonas aeruginosa strains producing high levels of persister cells in patients with cystic fibrosis. J. Bacteriol. *192*, 6191–6199.

Orman, M.A., and Brynildsen, M.P. (2013a). Establishment of a method to rapidly assay bacterial persister metabolism. Antimicrob. Agents Chemother. *57*, 4398–4409.

Orman, M.A., and Brynildsen, M.P. (2013b). Dormancy is not necessary or sufficient for bacterial persistence. Antimicrob. Agents Chemother. *57*, 3230–3239.

Raj, A., and van Oudenaarden, A. (2008). Nature, nurture, or chance: stochastic gene expression and its consequences. Cell *135*, 216–226.

Rotem, E., Loinger, A., Ronin, I., Levin-Reisman, I., Gabay, C., Shoresh, N., Biham, O., and Balaban, N.Q. (2010). Regulation of phenotypic variability by a threshold-based mechanism underlies bacterial persistence. Proc. Natl. Acad. Sci. USA *107*, 12541–12546.

Shah, D., Zhang, Z., Khodursky, A., Kaldalu, N., Kurg, K., and Lewis, K. (2006). Persisters: a distinct physiological state of E. coli. BMC Microbiol. *6*, 53.

Spoering, A.L., Vulić, M., and Lewis, K. (2006). GlpD and PlsB participate in persister cell formation in Escherichia coli. J. Bacteriol. *188*, 5136–5144.

Sun, J., Deng, Z., and Yan, A. (2014). Bacterial multidrug efflux pumps: mechanisms, physiology and pharmacological exploitations. Biochem. Biophys. Res. Commun. *453*, 254–267.

Taniguchi, Y., Choi, P.J., Li, G.W., Chen, H., Babu, M., Hearn, J., Emili, A., and Xie, X.S. (2010). Quantifying E. coli proteome and transcriptome with single-molecule sensitivity in single cells. Science *329*, 533–538.

Theodore, A., Lewis, K., and Vulić, M. (2013). Tolerance of Escherichia coli to fluoroquinolone antibiotics depends on specific components of the SOS response pathway. Genetics *195*, 1265–1276.

Vázquez-Laslop, N., Lee, H., and Neyfakh, A.A. (2006). Increased persistence in Escherichia coli caused by controlled expression of toxins or other unrelated proteins. J. Bacteriol. *188*, 3494–3497.

Veening, J.W., Smits, W.K., and Kuipers, O.P. (2008). Bistability, epigenetics, and bet-hedging in bacteria. Annu. Rev. Microbiol. *62*, 193–210.

Vega, N.M., Allison, K.R., Khalil, A.S., and Collins, J.J. (2012). Signaling-mediated bacterial persister formation. Nat. Chem. Biol. *8*, 431–433.

Webber, M.A., and Piddock, L.J. (2003). The importance of efflux pumps in bacterial antibiotic resistance. J. Antimicrob. Chemother. *51*, 9–11.

Wu, Y., Vulić, M., Keren, I., and Lewis, K. (2012). Role of oxidative stress in persister tolerance. Antimicrob. Agents Chemother. *56*, 4922–4926.

Zechini, B., and Versace, I. (2009). Inhibitors of multidrug resistant efflux systems in bacteria. Recent Pat Antiinfect Drug Discov *4*, 37–50.

Ziervogel, B.K., and Roux, B. (2013). The binding of antibiotics in OmpF porin. Structure *21*, 76–87.

Molecular Cell, Volume *62*

Supplemental Information

Enhanced Efflux Activity Facilitates

Drug Tolerance in Dormant Bacterial Cells

Yingying Pu, Zhilun Zhao, Yingxing Li, Jin Zou, Qi Ma, Yanna Zhao, Yuehua Ke, Yun Zhu, Huiyi Chen, Matthew A.B. Baker, Hao Ge, Yujie Sun, Xiaoliang Sunney Xie, and Fan Bai

Supplemental Figures and Legends

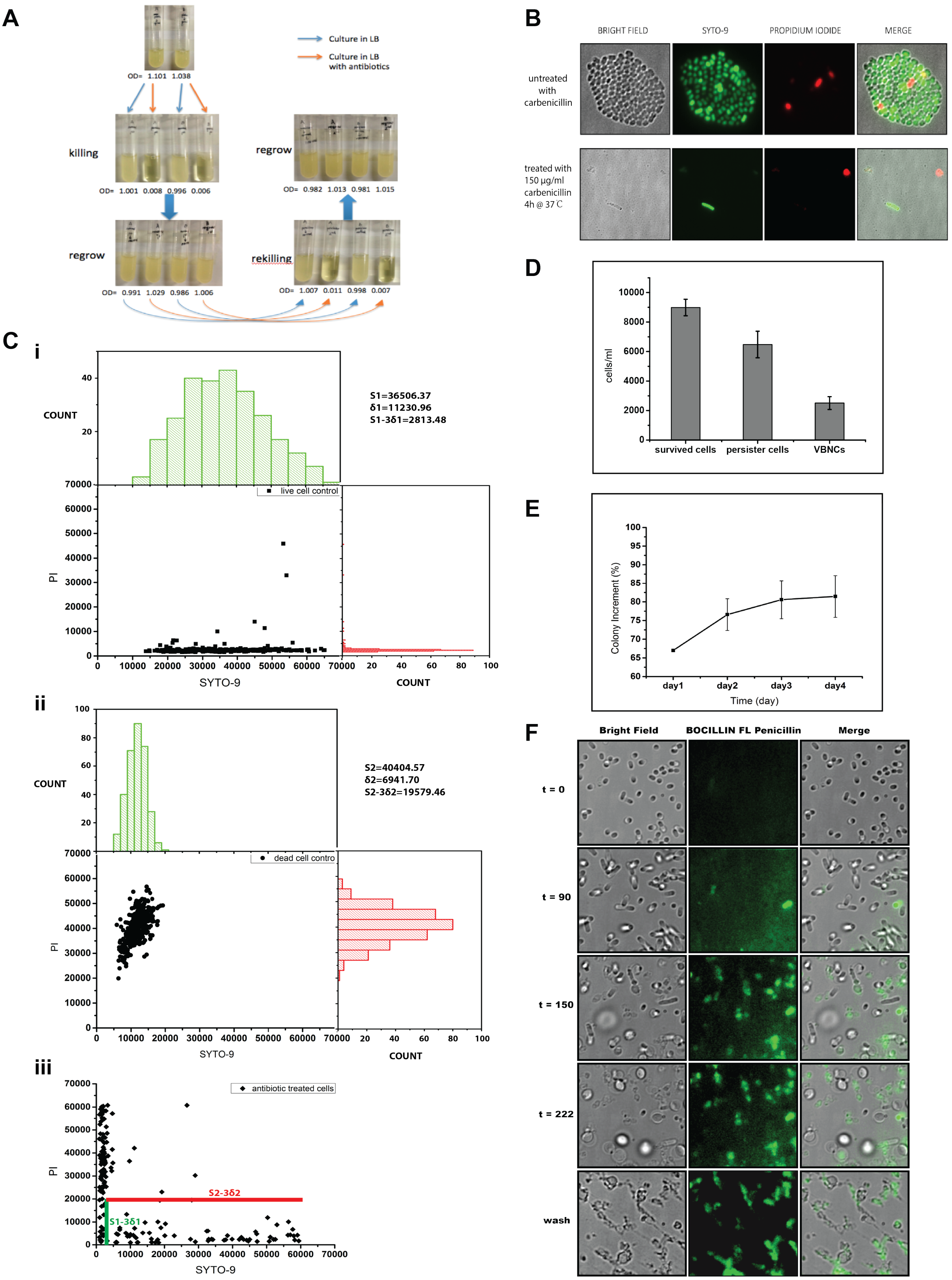

Figure S1. Related to Figure 1

(A) Drug tolerance and regrowth assay. In order to verify that what we have studied is persistence rather than resistance, we tested the drug tolerance and regrowth properties of the cells. The overnight culture of BW25113 strain was diluted 1:20 into fresh LB, which was then split into two tubes. For one tube 150 μg/ml carbenicillin (Sigma) was added and the other without antibiotic was set as control, and the culture was returned to the 37°C shaker for 4 hours to get drug tolerant cells. These cells were collected by centrifugation and re-suspended in fresh LB to resume growth. The killing and regrowth procedures were repeated again on the regrown cultures to ensure that persistence was transient and reversible. Two biological replicates were tested in this assay. **(B-D)** Persister cells are the main cell type in the survival population after carbenicillin treatment. Firstly we obtained the biphasic killing curves to make sure the survival cells we isolated were from the second killing phase (Figure S6). Then the remaining cells were collected with gentle centrifugation (4000 g, 3min). To quantify the number of live cells in the remaining population, cells were washed three times with staining buffer and stained with LIVE/DEAD BacLight Bacterial Viability Kits (Invitrogen). The washing and staining processes were all done in the presence of the same concentration of antibiotics used in the killing process to ensure the persistent nature of these cells. After staining, the remaining cells were divided equally into two tubes. One tube was used for plating and colony-counting assay to enumerate the number of persister cells on the next day. **(B)** The other tube was examined under a microscope to enumerate the total number of survived cells after antibiotic treatment. For this purpose, we evenly smeared a certain volume of cells on a 14mm*24mm rectangular gel pad (4% w/v of low melting agar) and waited for semi-dryness. Then the gel pad was covered by a coverslip and observed under a microscope. We randomly counted the number of cells in 20 image fields (0.493 mm * 0.493 mm/image field) with an automatic program. A live cell was defined as SYTO-9 positive and propidium iodide negative with intact cell shape. **(C)** Total survival cells quantification process. According to a previous protocol (Orman and Brynildsen, 2013), we used SYTO-9/PI staining to identify live cells from the remaining population after carbenicillin treatment. i, Red(PI) vs. Green (SYTO-9) fluorescence distribution of the live cell control (exponentially growing cells stained with SYTO-9 and PI). Fluorescence distribution of SYTO-9 of the live cell control was fitted with a Gaussian distribution (mean: S1; standard deviation: δ1); ii, Red (PI) vs. Green (SYTO-9) fluorescence distribution of the dead cell control (70% ethanol treated cells stained with SYTO-9 and PI). Fluorescence distribution of PI of the dead cell control was fitted with a Gaussian distribution (mean: S2; standard deviation: δ2); iii, Identification of total survival cells in the remaining population after four hours of 150ug/ml carbenicillin treatment. A live cell is defined as SYTO-9 signal above (S1- 3δ1, green line), PI signal below (S2- 3δ2, red line) and with intact cell shape. The population below (S1- 3δ1, green line) and below (S2- 3δ2, red line) is assumed to be cell capsids without nucleic acid. **(D)** By the method described above survived cells were counted as ~ 9000 cells/ml. Meanwhile, persister cells counted from colony counting assay equaled ~ 6000 cells/ml. If we assume the total survival cells consist of persister cells and viable but non-culturable cells (VBNCs), then the number of VBNCs can be estimated as ~3000 cells/ml. **(E)** The number of persisters with respect to incubation time, suggesting that VBNCs can switch to persister cells under suitable growth conditions. **(F)** Separate bright field and fluorescent images of the merged images shown in Figure 1D and Movie S1. From t = 0 to t = 222 is the killing process with a combination of 150 μg/ml carbenicillin and 20 μg/ml BOCILLIN FL Penicillin. The fresh medium without antibiotic was injected at t = 228 to allow persister cells to regrow (t = time in min). The bars indicate mean of at least three independent experiments; error bar indicates SD.

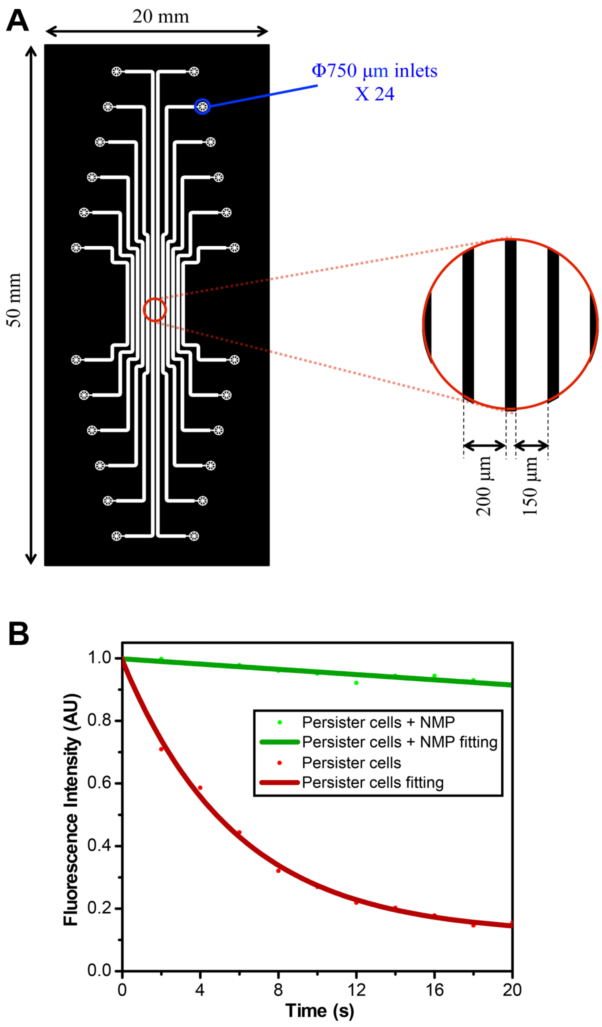

Figure S2. Related to Figure 2

(A) Microfluidic device design. 12 independent microfluidic channels were fabricated as an integrated pattern within a 20mm × 50mm area. The circles at the ends of channels are the inlet/outlet of the channel and holes with Φ0.75mm are punched through PDMS replicates at the same locations. The width of channel is 150 µm and the height is approximately 20 µm. **(B)** Intracellular fluorescent intensity decay after removing antibiotic in the medium is well fit by a single exponential function. The fast decay rate of fluorescent intensity in a persister cell (red) was inhibited by 1-(1-Naphthylmethyl) piperazine (NMP, 100µM) (green). Our results demonstrate that when treating with efflux inhibitor, the fluorescence stabilizes in the persisters, indicating that diffusion was not responsible for the loss of fluorescence in that sample.

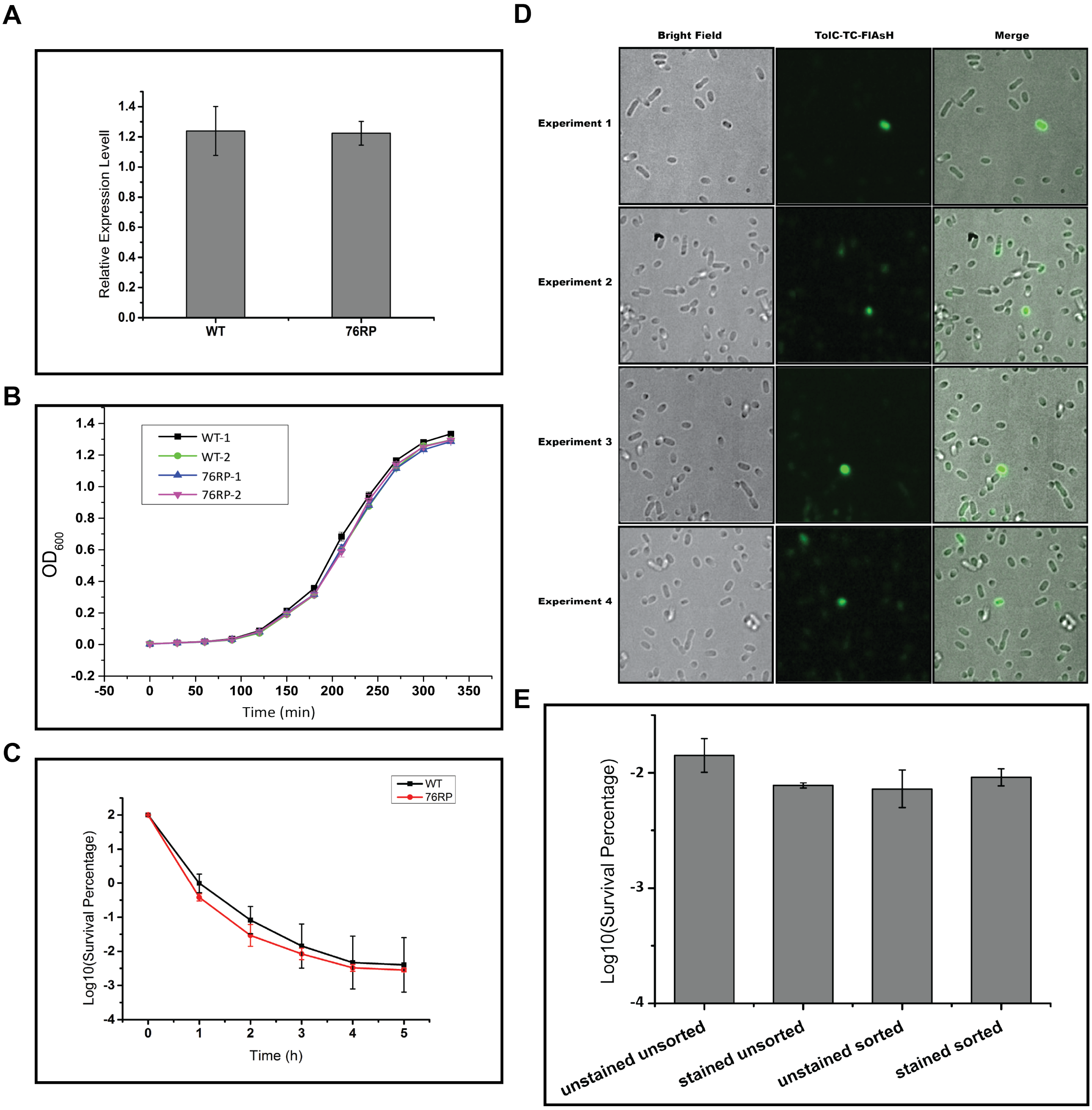

Figure S3. Related to Figure 4

(A-C) Comparison of 76RP strain and wild type strain in **(A)** *tolC* expression level measured by RT-qPCR. **(B)** growth curves. **(C)** killing curves under antibiotic treatment. **(D)** Separate bright field and fluorescent images of the merged images shown in Figure 4B and Movie S3. **(E)** Survival rates of stationary-phase cells of 76RP strain after carbenicillin treatment are not affected either by FlAsH staining or cell sorting. The bars indicate mean of at least three independent experiments; error bar indicates SD.

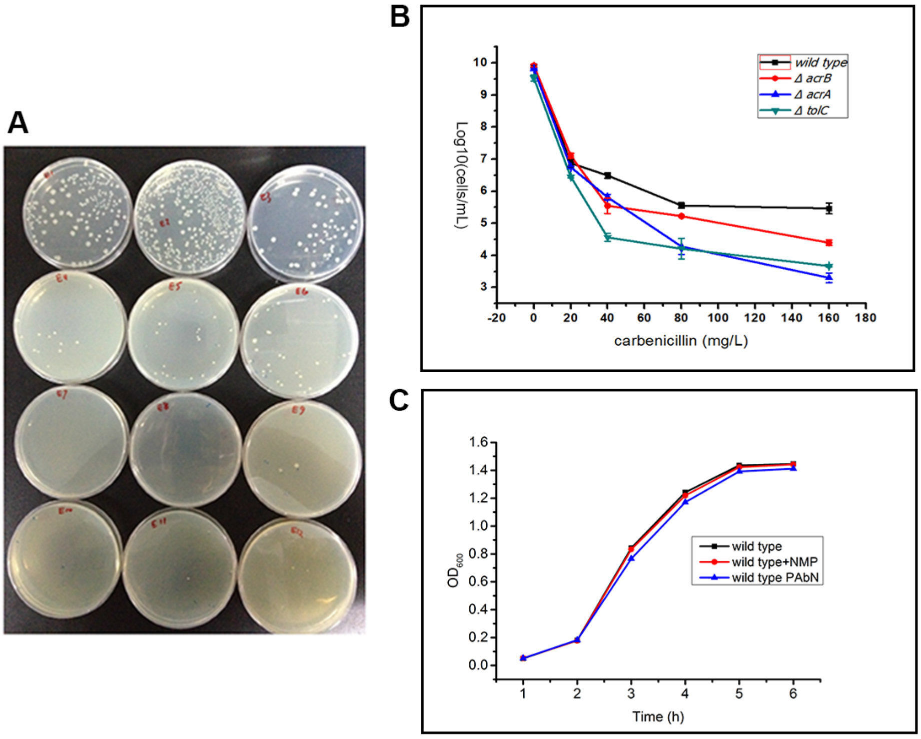

Figure S4. Related to Figure 5

(A-B) Correlation between bacterial persister formation frequency and gene expression of efflux pump systems. **(A)** The antibiotic susceptible measurement of *E. coli* wild type, *ΔacrA, ΔacrB* and *ΔtolC* strains under 160µg/ml carbenicillin treatment. The collected persister cells from each strain were diluted in the same volume of fresh LB and plated on LB agar for overnight culture. The colony counting was performed the next day. Three biological replicates were prepared for each strain. **(B)** Persister formation frequency of the four strains under antibiotic treatment of gradient concentrations. **(C)** Growth rate of wild type strain with or without efflux inhibitors NMP (100µM) and PAβN (100µM). Our results demonstrate that the pump inhibitors alone do not influence cell growth. The bars indicate mean of at least three independent experiments; error bar indicates SD.

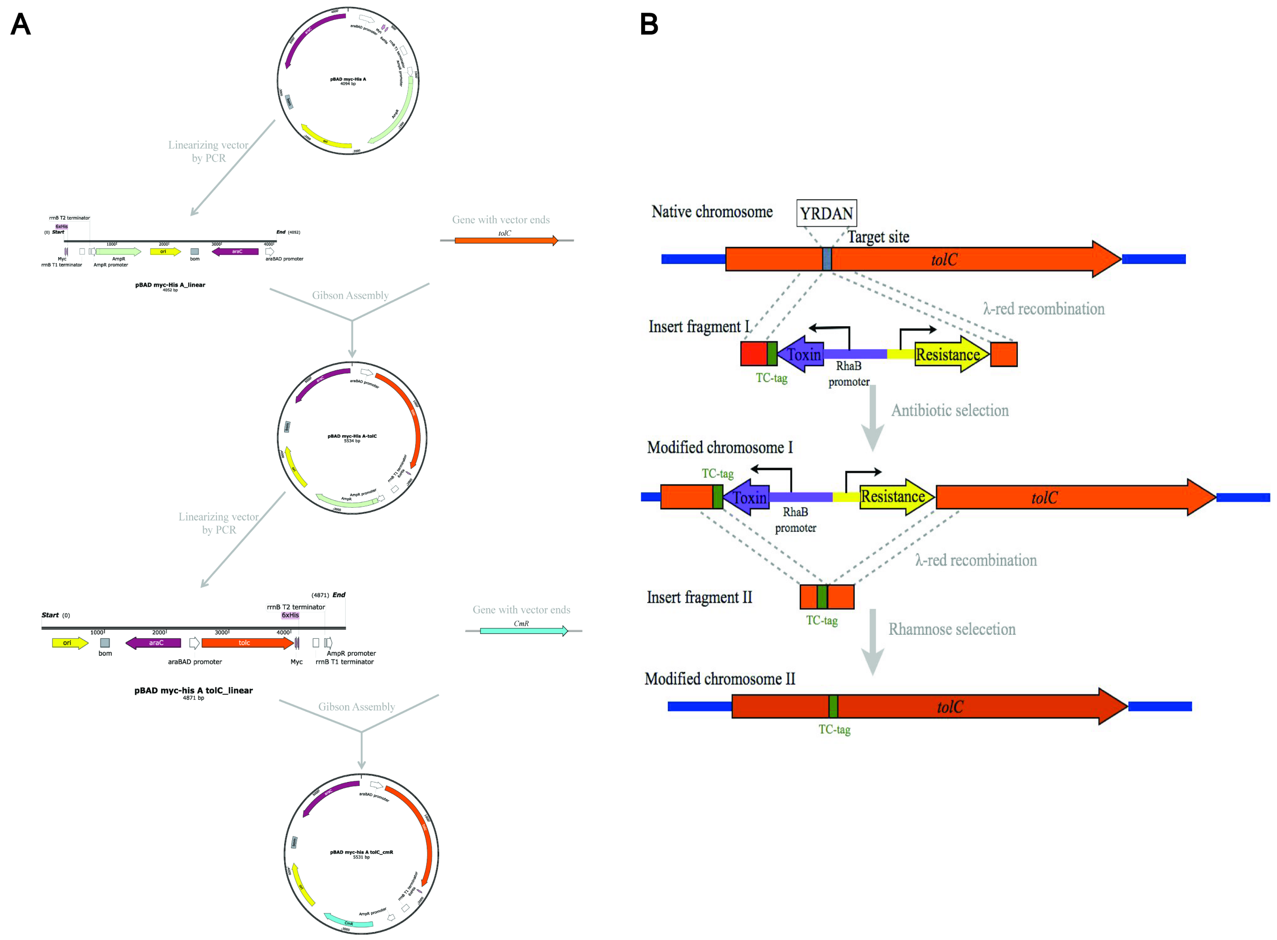

Figure S5. Construction of Strains Used in our Study, Related to Experimental Procedure

(A) Scheme of pBAD::myc-his_*tolC*_CmR construction. The PCR product of *tolC* with vector overlapping sequence was amplified from BW25113 and inserted into pBAD vector by *in vitro* Gibson Assembly method. The ampicillin resistant gene was replaced with chloramphenicol resistance gene by the same method. **(B)** Scheme of construction of TC tagged *tolC* 76RP strain. 76th~81th amino acids (YRDAN) of TolC protein, part of the linker between TolC S1 and S2 α-helixes, were firstly replaced with TC-Toxin-CmR cassette by λ-red recombination and then selected with chloramphenicol. The Toxin-CmR sequence was knocked out by another round of λ-red recombination with fusion fragment *tolC*-TC and selected with Rhamnose that would activate toxin expression.

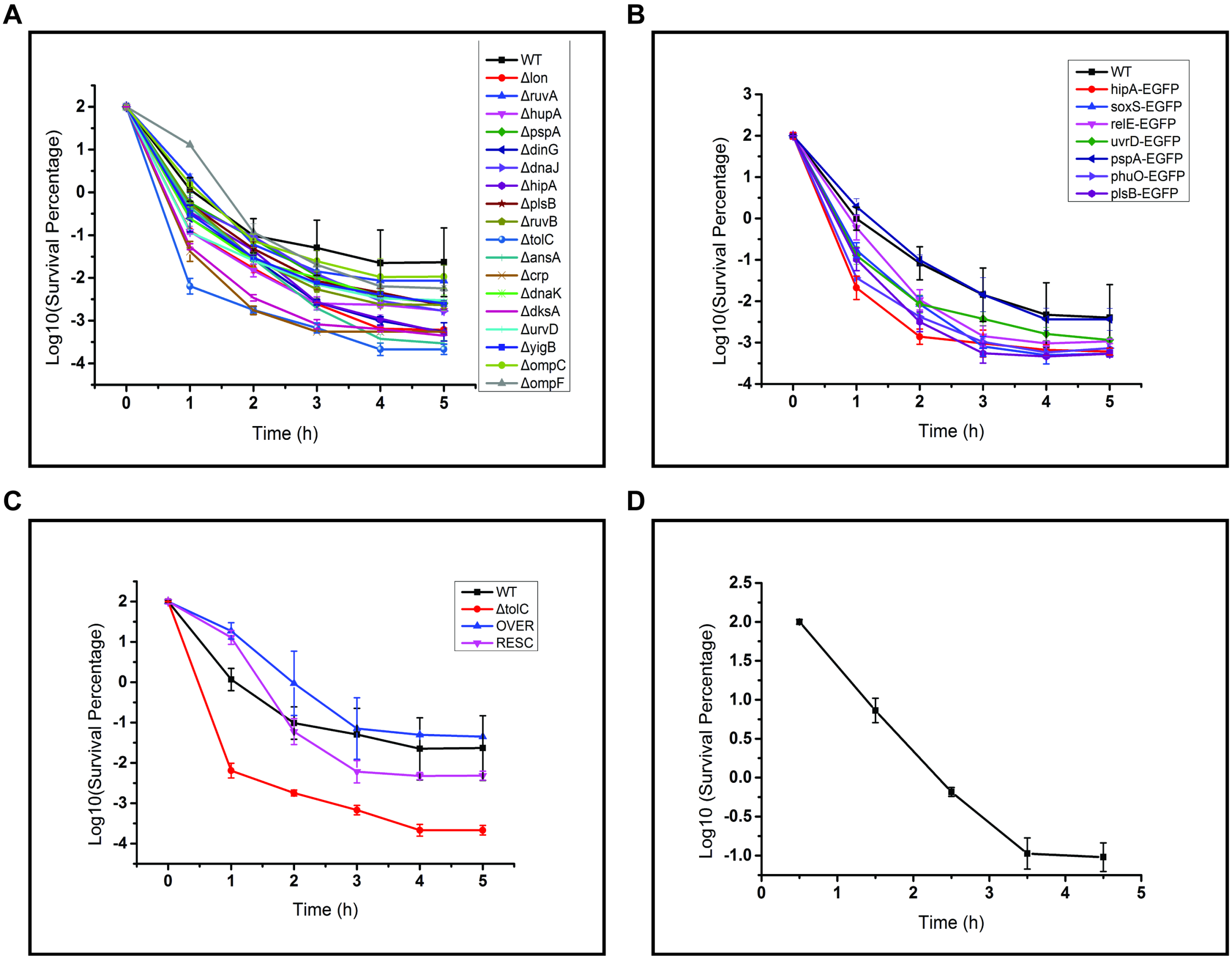

Figure S6. Biphasic Killing Curves of Strains Used in our Study, Related to Experimental Procedure

(A) Biphasic killing curves of all the knockout strains used in Figure 6C, confirming that the persisters we analyzed were from the second killing phase. (To note, *ΔompC* and *ΔompF* strains have lower survival rate than wild-type, although they show less antibiotic accumulation. This might because deletion of *ompC* or *ompF* influences other pathways that are critical to bacterial survival. Since the expression levels of *ompC* and *ompF* were not significantly different between persisters and total cells, their role in persister formation is not the main focus of this work.) **(B)** Biphasic killing curves of all the EGFP labeled strains used in Figure 6B, confirming that the persisters we analyzed were from the second killing phase. **(C)** Biphasic killing curves of *tolC* knockout strain, *tolC* overexpression strain and *tolC* rescued strain used in Figure 5A, 5B, 5C and 5D, confirming that the persisters we analyzed were from the second killing phase. **(D)** Biphasic killing curves of cells in the temperature control chamber on the microscope used in our time-lapse movies. This confirmed that the persisters we analyzed were from the second killing phase before we removed antibiotic and watched their re-growth. The carbenicillin concentration was 150μg/mL in determining the surviving curves. The bars indicate mean of at least three independent experiments; error bar indicates SD.

Supplemental Movies and Legends

Movie S1. Time-lapse Microscopy Showing the Persister Cells Accumulating Less Cellular Antibiotics, Related to Figure 1.
Total cells from stationary phase of BW25113 were treated with 150 µg/ml of carbenicillin and 20 µg/ml BOCILLIN in medium (90% (v/v) M9 + 10% (v/v) LB medium + 0.15% (w/v) methylcellulose) for 222 min. The bright field and fluorescent images were acquired every 6 min automatically. Then fresh medium (90% (v/v) M9 + 10% (v/v) LB medium + 5% (w/v) methylcellulose) was injected to remove antibiotic at t = 228 min, which would allow survived cells to resume growth. Then the bright field images were acquired every 6 min automatically. This whole process was performed at 37°C.

Movie S2. Bacteria Efflux Rate Measurement by Single-cell Level Time-lapse Microscopy, Related to Figure 2.
Untreated total cells and persisters from wild type *E. coli* stained with BOCILLIN were immobilized in a poly-L-lysine coated PDMS microfluidic device. The imaging was begun as soon as the antibiotic containing medium was washed away and replaced by fresh M9 minimal medium (t = 0s) and the imaging process lasted for 2 min. Imaging conditions: exposure time = 50ms; interval = 2s; light intensity (λ = 488nm, I = 20mW/cm^2). Propidium iodide was used in order to distinguish dead cells from persisters.

Movie S3. Time-lapse Microscopy Showing that Persister Cells Expressing a High Level of TolC Protein, Related to Figure 4.
Total cells from stationary phase of 76RP stained with FlAsH were treated with 150 µg/ml of carbenicillin in medium (90% (v/v) M9 + 10% (v/v) LB medium + 0.15% (w/v) methylcellulose) for 210 min. Then fresh medium (90% (v/v) M9 + 10% (v/v) LB medium + 5% (w/v) methylcellulose) was injected to remove antibiotics at t = 210 min, which would allow survived cells to resume growth. The first image is the merge of bright field and fluorescent channels showing the level of TolC expression at t = 0 min. Then the bright field images were acquired every 5 min automatically. The latent time before regrowth varies from cell to cell. The whole process was performed at 37°C. Propidium iodide was used in order to distinguish dead cells from live cells.

Supplemental Tables and Legends

Table S1. RNA-seq and Differential Expression Profile Analysis, Related to Figure 3 and 6.
We compared transcriptome profiles of persister and total cells of wild type *E.coli* to determine changes in gene expression. Overnight culture was diluted 1:15 into 200ml LB or M9 medium and split into two flasks. One flask was then treated with 150 μg/ml carbencillin to get persister cells and the other was set as untreated control to get total cells. After 4 hours culture at 37°C, RNA was isolated and processed to transcriptome sequencing. The resulting sequencing data were further normalized to get gene expression RPKM value which allow the analysis of changes in gene expression between persister and total cells. We chose significantly changed genes by selecting those with log2 (persister/total) >1 and p value < 0.005. Two data sets were merged to obtain a common set of genes up- or down-regulated in two independent biological replicates. As showed in Table S1, 298 genes were up-regulated and 33 genes were down-regulated in their expression in persisters in both experiments.

Table S2. Whole Genome Sequencing of Persister and Untreated Total Cells, Related to Figure 3.
Genetic mutations in upstream regulators could also result in increased expression of efflux-associated genes, which has been well documented in bacterial antibiotic resistance (Poole, 2002; Wiercinska et al., 2015; Wren et al., 2015). In order to further confirm that there were no mutations occurring on efflux genes and their regulatory pathways, we performed whole genome sequencing on both total cells and persisters. As shown in this table, no mutation was found in efflux genes or in the related regulatory pathway in all three biological replicates of persisters. Moreover, no mutation was common to all three repeats, indicating mutations between persisters and total cells are random.

Table S3. Primer Pairs Used in this Study, Related to Experimental Procedure.

Supplemental Experimental Procedures

Bacterial strains and plasmid construction

pBAD::*tolC* plasmid was transformed by electroporation into BW25113 and JW5503 to generate the *tolC* overexpression strain and rescue strain, respectively. The *tolC* PCR product was amplified from BW25113 and inserted into pBAD/Myc-His A vector at Nco I and Hind III sites by the *in vitro* Gibson Assembly method. The ampicillin resistant gene was replaced with chloramphenicol (CAM) resistance cassette by the same method (Figure S5A). Luria-Bertani (LB) broth and LB agar media were used for culturing.

76RP strain was obtained by replacing TolC protein 76th~80th amino acids (YRDAN) that are part of the linker between TolC S1 and S2 α-helix with TC-Tag (CCPGCC) (Figure S5B). TolC expression level (Figure S3A), growth curve (Figure S3B) and killing curve (Figure S3C) showed the replacement did not compromise the functionality of TolC protein. Strains containing chromosomal *geneX-egfp* translational fusion or single *geneX* knockout mutants were constructed by λRed-mediated gene replacement (Datsenko and Wanner, 2000).

Fluorescent Microscopy

For intracellular fluorescent antibiotic accumulation measurement, the LB-cultured total and persister cells were collected, washed three times with M9 minimal medium and incubated with fluorescent antibiotic BOCILLIN for 30 min at 37°C with gentle shaking. Additional experiments proved that the staining time between 30~60 min would not significantly affect the result. The cells were then used directly for epifluorescence microscopy or total internal reflection fluorescence microscopy at the interface between the coverslip and the M9 minimal growth medium containing methyl-cellulose and BOCILLIN. For efflux rate measurement, we used PDMS microfluidics coated with poly-L-lysine for imaging. After being injected into channels, the cells were incubated for about 10 minutes at room temperature for stable resting. We started time-lapse epifluorescence imaging as soon as the medium containing antibiotics was washed away by new M9 minimal medium. For FlAsH staining, after being harvested and washed as described above, the bacteria were re-suspended in FlAsH-EDT_2 (Invitrogen) diluted to 20μM into M9 minimal, and were incubated at RT for 90 min. Then the bacteria were washed twice with BAL wash buffer supplied by Invitrogen. Propidium iodide from Invitrogen LIVE/DEAD staining kit was then used to re-suspend the bacteria in order to distinguish dead cells from surviving cells. The camera exposure time and gain was adjusted to prevent over-saturated image acquisition.

Image processing

Image analysis was done by ImageJ software (Fiji). Cell contours were constructed from bright field images. The background image and cell auto-fluorescence was subtracted from the fluorescent image. The integrated fluorescence intensity of the entire cell area was measured for each cell and normalized by cell area. The extracted data from time-lapse images was analyzed in Matlab (R2011a, Mathworks) using custom data processing script. For efflux rate assay, the cell intensity traces were fitted by single exponential function; the parameters were statistically analyzed and verified by *Student's t*-test.

Microfabrication

The microfluidic pattern (Figure S2A) was designed on AutoCAD 2004 (Autodesk Inc.) and output into a photomask film using a commercial photoploting service with a resolution of 20,000dpi. Photolithography was used to produce pattern on test-grade silicon wafer (University wafer). The pattern was developed by exposing UV-light to the wafer which was coated with a UV-curable exposy (SU8-2025, Micro-Chem) of 25 μm thick. Softlithography was used to produce microfluidic platform. Poly-dimethylsiloxane (PDMS), a low-cast, optically transparent silicon elastomer, was molded on the fabricated wafer by curing at 80°C for 20 minutes. Holes of Φ750 μm were punched through the inlet/outlet positions of the replicated PDMS sheet. The PDMS sheet was bonded to a coverslip (0.17 mm thick, 48 x 60 mm, FISHER) by treatment with an oxygen plasma cleaner.

RNA isolation and RNA-seq

The cells were lysed by lysozyme (400μg/ml, Ready-Lyse™ Lysozyme Solution, Epicenter) with addition of RNase inhibitor (Invitrogen) for 10min at room temperature. Total RNA was then extracted by using RNeasy Mini Kit (Qiagen). Ribosomal RNA was depleted by RiboMinus Transcriptome Isolation Kit (Invitrogen) to get mRNA that was then cleaned up using RNeasy Mini Spin columns (Qiagen). The resulting transcriptome RNA was used for RNA-seq library construction using NEBNext mRNA Library Prep Reagent set for Illumina (NEB) according to manufacturer's protocol. Briefly, mRNA was fragmented to desired length and reverse transcripted into first strand cDNA. The single strand cDNA was used for double strand DNA synthesis followed by end repair, dA-tailing, adaptor ligation and PCR amplification. Before sequencing, the library was examined by length determination and quantitative PCR certification. These constructed libraries were then sequenced by Illunima Hiseq 2000 platform by paired-end chemistry.

DNA isolation and genome sequencing

The cells were lysed by lysozyme (400μg/ml, Ready-Lyse™ Lysozyme Solution, Epicenter) for 15 min at room temperature. DNA was then extracted by using DNeasy Blood & Tissue Kit (Qiagen). The genome sequencing library was constructed by using NEBNext DNA Library Prep Master Mix for Illumina (NEB, E6040). Briefly, genome DNA was fragmented into desired length, followed by end repair, dA-tailing, adaptor ligation and PCR amplification. Before sequencing, the library was examined by length determination and quantitative PCR certification. The constructed DNA libraries were then sequenced by Illunima Hiseq 2000 platform by paired-end chemistry.

Sequencing data analysis

DNA-seq reads were aligned to *Escherichia coli* reference NC_012971.2 (http://www.ncbi.nlm.nih.gov/assembly/GCF_000022665.1/) using the Burrows–Wheeler Aligner. Sorting of aligned reads, duplicate removal and detection of variations were done with Samtools 0.1.18 . The functional effect of variants was annotated with SNPEFF 3.0 . SNVs/INDELs were called for variations that presented in persister cells but not in the matched total cells. Differential gene expression analysis of RNA-seq was done with TopHat 2.0.12 and Cufflinks 2.2.1.

Reverse transcription and Quantitative PCR

The total RNA was extracted as described above. Reverse transcription was performed to yield cDNA by using SuperScript® III Reverse Transcriptase (Invitrogen) with random primers following standard protocol. Target gene expression level was measured by quantitative PCR with *mreB* as reference gene by using Brilliant II SYBR Green QPCR Master Kit (Agilent Technologies) on 7500 Real Time PCR System (Applied Biosystem).

Supplemental References

Datsenko, K.A., and Wanner, B.L. (2000). One-step inactivation of chromosomal genes in Escherichia coli K-12 using PCR products. Proc. Natl. Acad. Sci. USA *97*, 6640–6645.

Poole, K. (2002). Outer membranes and efflux: the path to multidrug resistance in Gram-negative bacteria. Current pharmaceutical biotechnology 3, 77-98.

Wiercinska, O., Chojecka, A., Kanclerski, K., Rohm-Rodowald, E., and Jakimiak, B. (2015). Significance of efflux pumps in multidrug resistance of Gram-negative bacteria. Medycyna doswiadczalna i mikrobiologia 67, 55-62.

Wren, M.S., Ganguli, K., Paridington, P.E., Dimitrijevic, M., McMahon, B.H., Schweizer, H., and Gupta, G. (2015). 58 Multi-drug resistance efflux pumps in bacteria: how they work? Journal of biomolecular structure & dynamics 33 Suppl 1, 39.
